# Preclinical therapies to prevent or treat fracture non-union: A systematic review

**DOI:** 10.1371/journal.pone.0201077

**Published:** 2018-08-01

**Authors:** Philippa M. Bennett, Sarah K. Stewart, Janine Dretzke, Danai Bem, Jowan G. Penn-Barwell

**Affiliations:** 1 Institute of Naval Medicine, Crescent Road, Alverstoke, Hampshire, United Kingdom; 2 Royal Centre for Defence Medicine, Queen Elizabeth Hospital, Edgbaston, Birmingham, United Kingdom; 3 Institute of Applied Health Research, College of Medical and Dental Sciences, University of Birmingham, Edgbaston, Birmingham, United Kingdom; University of Mississippi Medical Center, UNITED STATES

## Abstract

**Background:**

Non-union affects up to 10% of fractures and is associated with substantial morbidity. There is currently no single effective therapy for the treatment or prevention of non-union. Potential treatments are currently selected for clinical trials based on results from limited animal studies, with no attempt to compare results between therapies to determine which have the greatest potential to treat non-union.

**Aim:**

The aim of this systematic review was to define the range of therapies under investigation at the preclinical stage for the prevention or treatment of fracture non-union. Additionally, through meta-analysis, it aimed to identify the most promising therapies for progression to clinical investigation.

**Methods:**

MEDLINE and Embase were searched from 1^St^ January 2004 to 10^th^ April 2017 for controlled trials evaluating an intervention to prevent or treat fracture non-union. Data regarding the model used, study intervention and outcome measures were extracted, and risk of bias assessed.

**Results:**

Of 5,171 records identified, 197 papers describing 204 therapies were included. Of these, the majority were only evaluated once (179/204, 88%), with chitosan tested most commonly (6/204, 3%). Substantial variation existed in model design, length of survival and duration of treatment, with results poorly reported. These factors, as well as a lack of consistently used objective outcome measures, precluded meta-analysis.

**Conclusion:**

This review highlights the variability and poor methodological reporting of current non-union research. The authors call for a consensus on the standardisation of animal models investigating non-union, and suggest journals apply stringent criteria when considering animal work for publication.

## Introduction

Fracture non-union can be defined as occurring when the normal healing processes of bone cease to the extent that solid healing cannot occur without further intervention[1]. The condition is estimated to affect 5–10% of fractures[2, 3], with wide variation depending on anatomical location[4]. The negative effect on quality of life associated with non-union has been demonstrated as being greater than that of diabetes mellitus, stroke and acquired immunodeficiency syndrome[5], with substantial financial consequences[6].

The failure of a fracture to unite is multifactorial and the result of both predisposing and contributing factors[1, 7]. There is no consensus or accepted guidelines for the treatment of non-union, but most current management strategies involve hospital admission and revision surgery, frequently using bone graft or synthetic substitutes, with varied and unpredictable results. In order to either primarily prevent non-union, increase the likelihood of success of revision surgery, or potentially offer an alternative to surgery, researchers continue to evaluation novel therapies in this field.

Preclinical studies are defined as those using animals to determine if a treatment is likely to be effective, before progression to testing in humans [8].

It is currently not clear on what basis researchers select potential therapies for translation into clinical studies. It is likely that positive results from a single, or a small number, of animal studies are used to justify progression to clinical trial. However, it is problematic to rely on the positive effects of a therapy in a single animal study to justify direct translation to clinical testing due to the likely existence of bias and methodological weakness. There is no evidence that researchers in this field have compared different preclinical studies in an attempt to determine which therapies are the most promising and therefore should be prioritised for translation into clinical studies.

Systematic reviews summarise the literature for a defined research question; when combined with a meta-analysis of results they are considered to represent the highest level in the hierarchy of evidence[9]. Despite this, meta-analyses are reliant upon the quality of data in the original studies included, and can risk propagating any errors included in the original research. The methodology for systematic reviews of preclinical research is still evolving, but it is recognised that the technique has the potential to clarify the existing evidence base and potentially increase the precision of effect estimates through meta-analysis[10, 11]. To date there has not been a systematic review or meta-analysis of preclinical studies aiming to prevent or treat fracture non-union.

The aim of this systematic review was firstly to establish the range of therapies under investigation at the preclinical stage for the prevention or treatment of fracture non-union. Secondly, by conducting a meta-analysis of results of methodologically similar studies, it aimed to systematically and objectively identify the most promising therapies for progression to clinical investigation.

## Materials and methods

### Search strategy and inclusion criteria

Full methodological details can be found in the previously published protocol[12]. The protocol was registered with Collaborative Approach to Meta-Analysis and Review of Animal Data from Experimental Studies (CAMARADES)[13]. A summary of the methods is reported below. Reporting of the full systematic review was in accordance with the Preferred Reporting Items for Systematic Reviews and Meta-Analyses (PRISMA) guidelines[14], (S1 Table).

MEDLINE and Embase were searched via Ovid from 1^st^ January 2004 to 10^th^ April 2017 (see S2 Table for full search strategy). The citation lists of included studies were searched for additional studies. In a deviation from the methodology published in the study protocol, due to the large volume of studies retrieved from the primary searches, no further additional sources were searched.

Two reviewers (PMB/SKS) independently screened titles and abstracts. Where eligibility for inclusion could not be determined from the abstract the full manuscript was obtained and reviewed for clarification. Any disagreements were resolved through discussion with a third reviewer (JPB). Controlled trials evaluating an intervention to prevent or treat non-union and measuring bone formation were eligible for inclusion; the focus of this review was to examine preclinical therapies with clinical potential and so treatments which had already been evaluated in a clinical study were excluded. Full inclusion and exclusion criteria were listed in the previously published protocol and are summarised in Table 1. Relevant preclinical studies evaluating therapies that had subsequently progressed to clinical trial were excluded, unless the therapy was combined with a novel therapy.

**Table 1:** Summary of study inclusion and exclusion criteria.

**Inclusion Criteria**	
Types of studies	Controlled trials
	Unpublished and published works
Types of participants	Mammalian model testing an intervention to treat or prevent fracture non-union
	Induced co-morbidities
Intervention	Interventions aim to: Prevent non-union Treat non-union Promote or accelerate healing of a bony defect Treat or ameliorate delayed union
	Administered after formation of a bony defect
	Established interventions in a novel vehicle
Comparator	Control group described receiving: No treatment Current standard of care Alternative treatment
Outcome measures	Quantifiable measure of bone formation through radiological and/or histological means
**Exclusion Criteria**	
Types of studies	Review articles
Types of participants	Clinical trials
Intervention	Any intervention that has subsequently progressed to clinical trial

**Fig 1:**
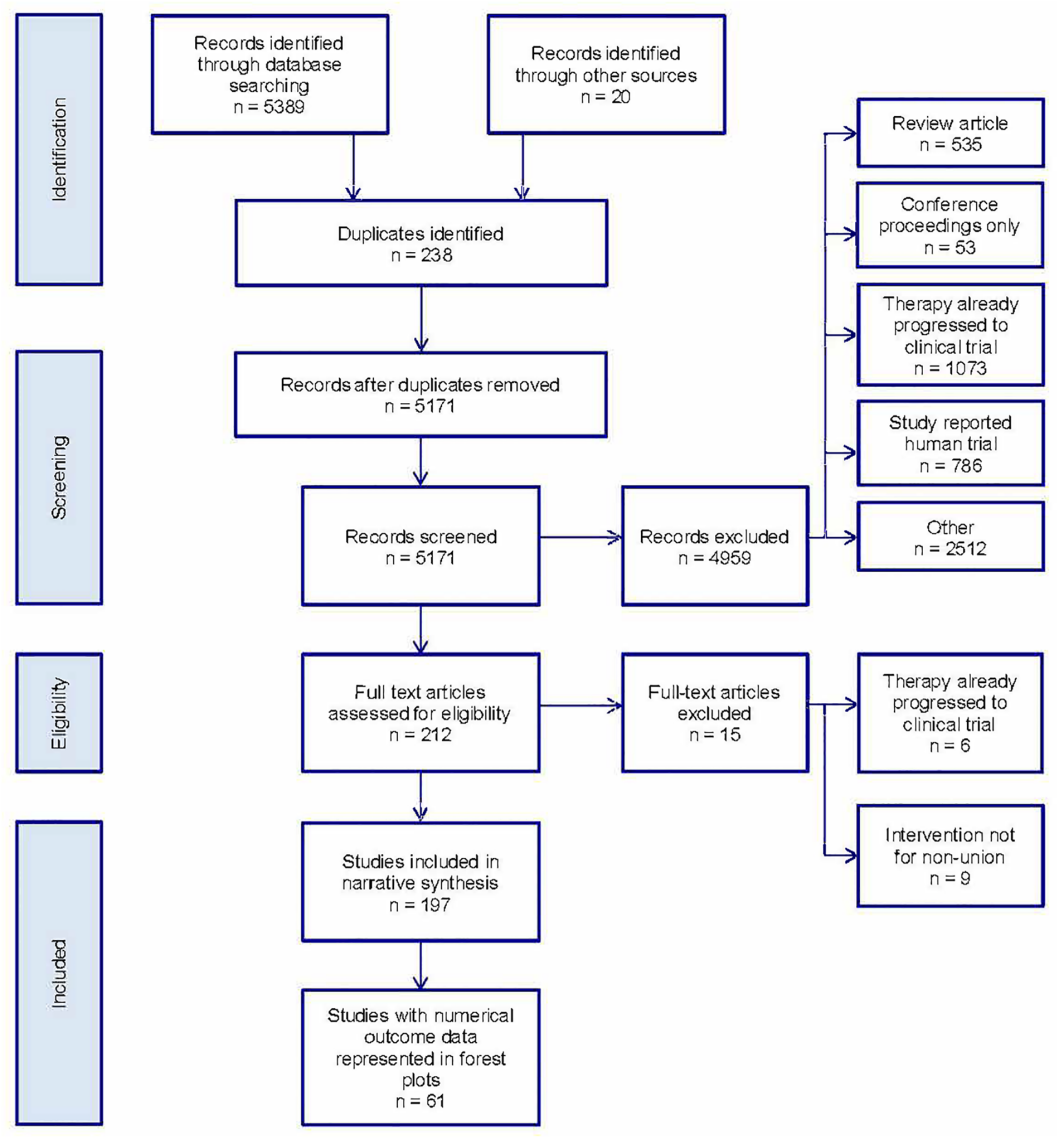
PRISMA flow diagram for study inclusion/exclusion. Preferred Reporting Items for Systematic Review and Meta-Analyses (PRISMA) flow diagram detailing numbers of studies excluded and reasons at each stage of the review process.

After duplicates were removed, 5,171 records were identified in the literature search as shown in the PRISMA flow diagram (Fig 1). After inclusion/exclusion criteria were applied 197 studies were included in the systematic review. The commonest single reason for study exclusion (1,073 studies, 21%) was that the article described a therapy that had already progressed to clinical trial.

### Data extraction and risk of bias assessment

Data relating to the model, defect location and method of creation, length of survival, number of animals included, outcome measures (radiological or histological) were extracted from manuscripts.

Where incomplete data was provided in the manuscript authors were contacted for clarification: of the 64 authors contacted, only 9 replied with the required information (14%). Numerical data extraction from papers presenting results in graphical format only was performed using ImageJ v.2.0 software (National Institute of Health, Bethesda, MD) using a standardised method[15, 16].

The Systematic Review Centre for Laboratory Animal Experimentation’s (SYRCLE) risk of bias tool was used to assess risk of bias across all studies[17]. The SYRCLE tool assesses ten domains across six types of bias: selection bias (sequence generation, baseline characteristics, allocation concealment), performance bias (random housing, blinding), detection bias (random outcome assessment, blinding), attrition bias (incomplete outcome data), reporting bias (selecting outcome reporting) and other sources of bias. Risk of bias assessment was performed by one author (PMB or SKS). Each domain was given a rating of high risk, low risk or unclear where information was incomplete or not reported. These ratings were based on the signalling questions designed to assist judgement, as detailed in the SYRCLE tool[17].

### Analysis

Where studies reported sufficient data (numbers in intervention and control group, mean and standard deviation), results for the most consistently reported measures (bone formation (%), bone volume (mm^3^) or bone density (mg/cm^3^)) were represented in forest plots for illustrative purposes. Results for the remaining studies were tabulated. Where several time-points were reported, only the longest follow-up was considered.

Therapies were grouped into the following nine categories:

- Animal derivatives
- Plant extracts
- Minerals/elements/chemicals
- Pharmaceuticals
- Cells/tissues
- Vibration/motion
- Light/lasers
- Gases
- Human proteins/hormones

If a therapy related to more than one category, it was included in both it pertained to (e.g. mesenchymal stem cells with insulin-like growth factor-1 was recorded in both the ‘cells/tissues’ and ‘human proteins/hormones’ categories.) Combination therapies using both an established therapy already in clinical trial with a novel preclinical therapy were again recorded in both categories to which they pertained.

## Results

### The spectrum of potential treatments

The 197 included studies evaluated a total of 204 different interventions (Table 2). The objective of approximately half of all studies was to promote or accelerate healing of a bony defect (103/197, 52%) or treat non-union (93/103, 47%), with further information available in S3 Table. The majority of therapies (179/204 (88%)) were only evaluated once, while five interventions (chitosan [18–23], adipose stromal cells [24–27], erythropoietin [28–31], vascular endothelial growth factor [32–35] and SDF-1 [36–38]) were investigated by multiple studies (Table 3). Chitosan as a single therapy was evaluated by six studies: four of these found significantly greater bone formation in the intervention group compared to control [18, 20–22], with further detail in Table 3.

**Table 2:** Number of evaluations under investigation by category*.

Group	Number of evaluations included in tables	Number of evaluations included in forest plots	Total
Animal derivatives	27	5	**32**
Plant extracts	23	13	**36**
Minerals / elements / chemicals	25	7	**32**
Pharmaceuticals	16	13	**29**
Cells / tissues	32	18	**50**
Vibration / motion	2	5	**7**
Light / lasers	3	0	**3**
Gases	3	5	**8**
Human proteins / hormones	59	41	**100**
**Total**	**190**	**107**	**297**

*Combination therapies are duplicated in **all** groups they pertain to, e.g. mesenchymal stem cells + vascular endothelial growth factor will be counted in “cells / tissues” and “human proteins / hormones”.

Single therapies tested in multiple concentrations are counted more than once, e.g. Ngueguim 2012 evaluates two plant based therapies: both therapies are evaluated at three different concentrations, thereby contributing 6 evaluations.

A total of 197 studies were included, investigating a total of 204 distinct therapies.

Total number of studies included in tables = 136, total number of studies included in forest plots = 61.

**Table 3:** Most frequently evaluated therapies across all studies (n = 197).

Therapy	Number of studies evaluating therapy	Direction of effect
Chitosan	6	Four studies [18, 20–22] favoured intervention over control. One study [19] favoured control over intervention. One study [23] showed no difference between intervention and control.
Adipose stromal cells	4	Two studies [25, 27] favoured intervention over control. Two studies [24,26] showed no difference between intervention and control.
Erythropoietin	4	Four studies [28–31] showed no difference between intervention and control
Vascular endothelial growth factor	4	Two studies [32, 35] favoured intervention over control. Two studies [33, 34] showed no difference between intervention and control.
SDF-1	3	Two studies [36, 38] favoured intervention over control. One study [37] showed no difference between intervention and control.
Therapies tested twice	40	
Therapies tested once	179	

### Risk of bias

**Fig 2:**
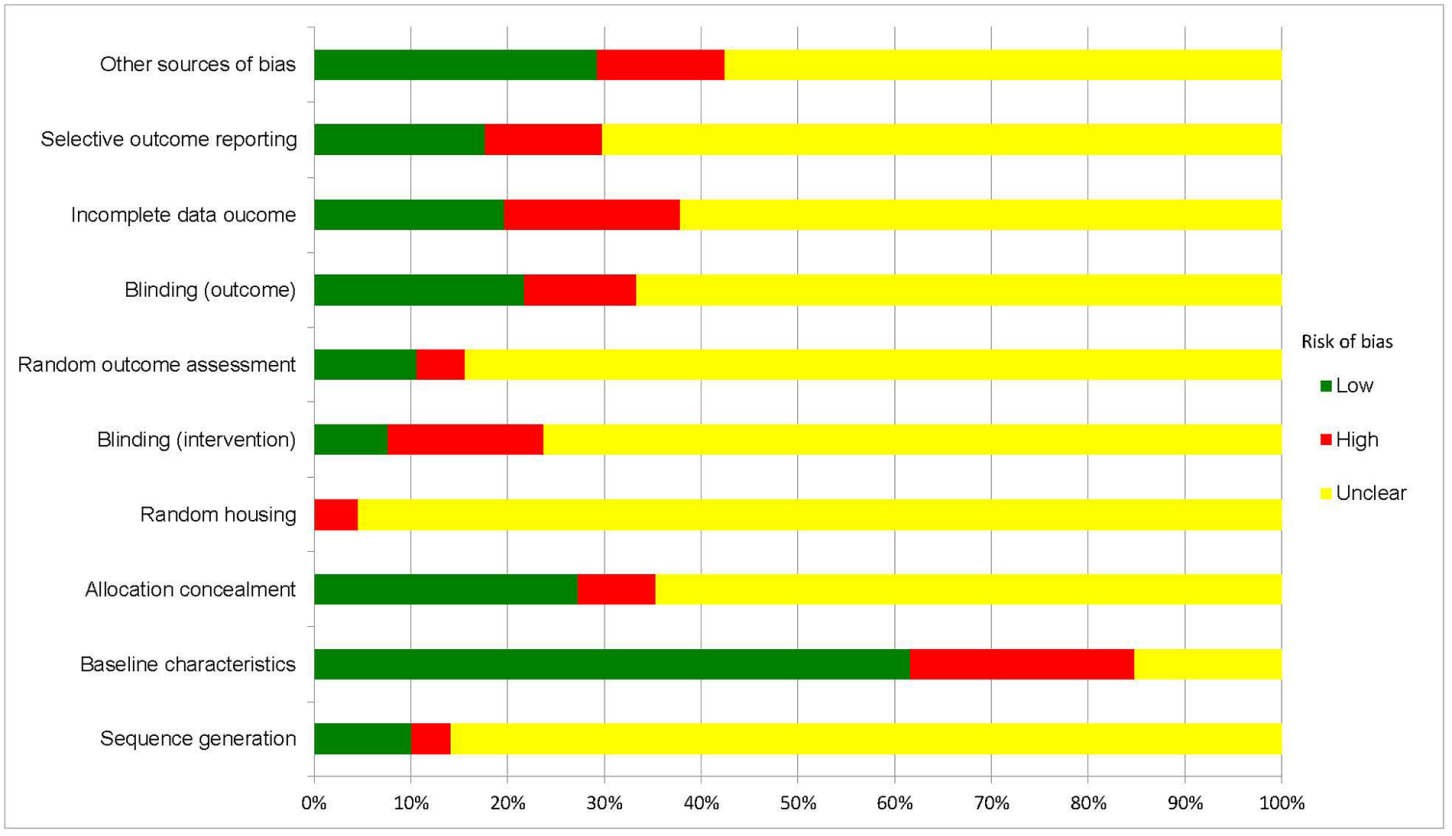
Risk of bias analysis. Bias assessed as per the Systematic Review Centre for Laboratory Animal Experimentation’s (SYRCLE) tool for all 197 studies included.

Details necessary to assess risk of bias were vastly underreported, particularly with regard to random housing, random outcome assessment (randomisation), sequence generation, blinding of outcome assessment and selective outcome reporting (Fig 2). Between 4 and 23% of studies were judged to be at high risk of bias for a given criterion. No study reported details for all ten domains of the SYRCLE tool.

**Fig 3:**
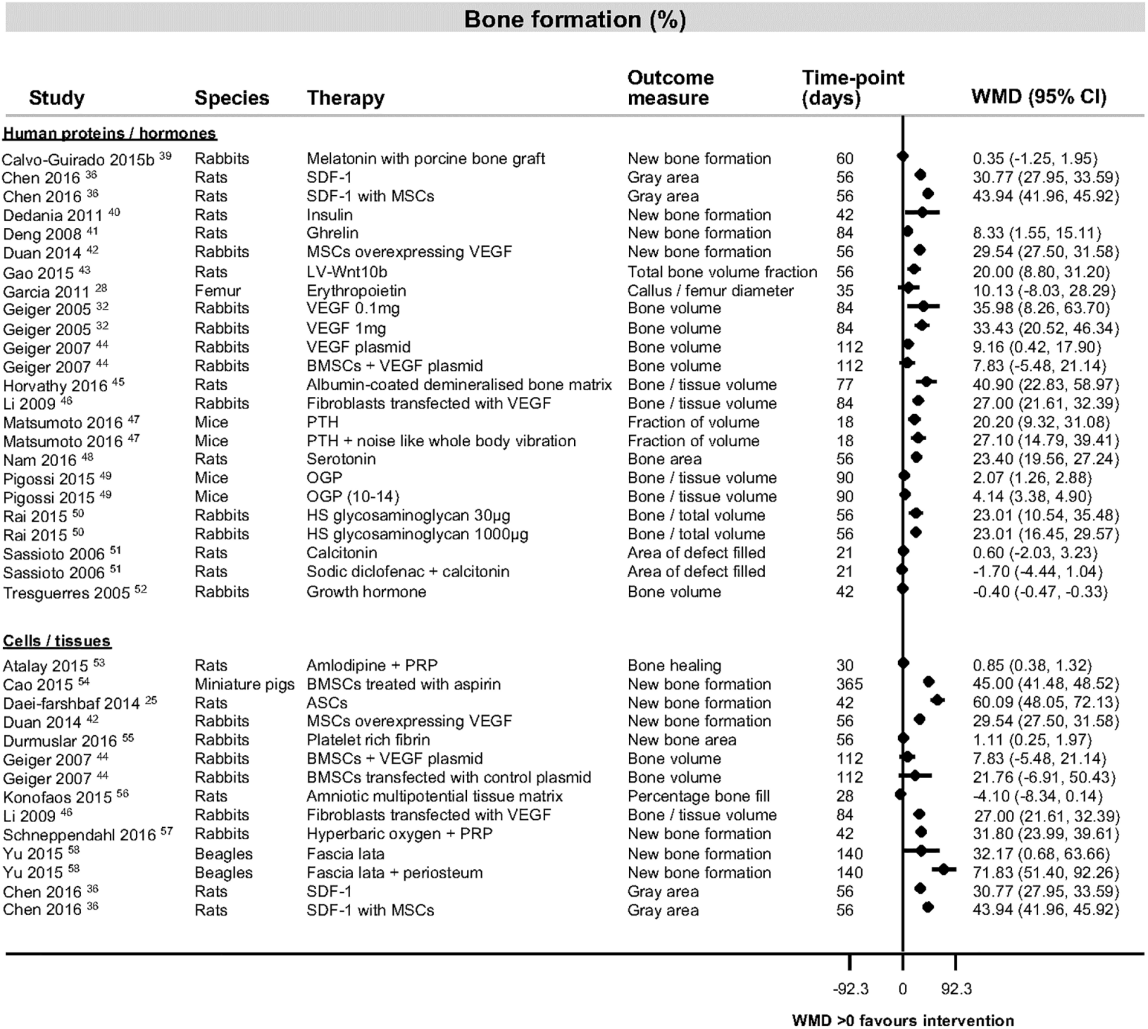
Bone formation data for studies looking at interventions of human proteins and hormones or cells and tissues. Forest plot illustrating mean difference in percentage of bone formation as measured by different histological or radiological measures. Abbreviations: ASCs, adipose tissue stem cells; BMSCs, bone marrow stromal cells; CI, confidence interval; HS, heparan sulphate; LV-Wnt10b, lentivirus vector encoding Wnt10b gene; MSCs, mesenchymal stem cells; OGP, osteogenic growth peptide; PRP, platelet rich plasma; PTH, parathyroid hormone; SDF-1, stromal cell derived factor 1; VEGF, vascular endothelial growth factor; WMD, weighted mean difference.

**Fig 4:**
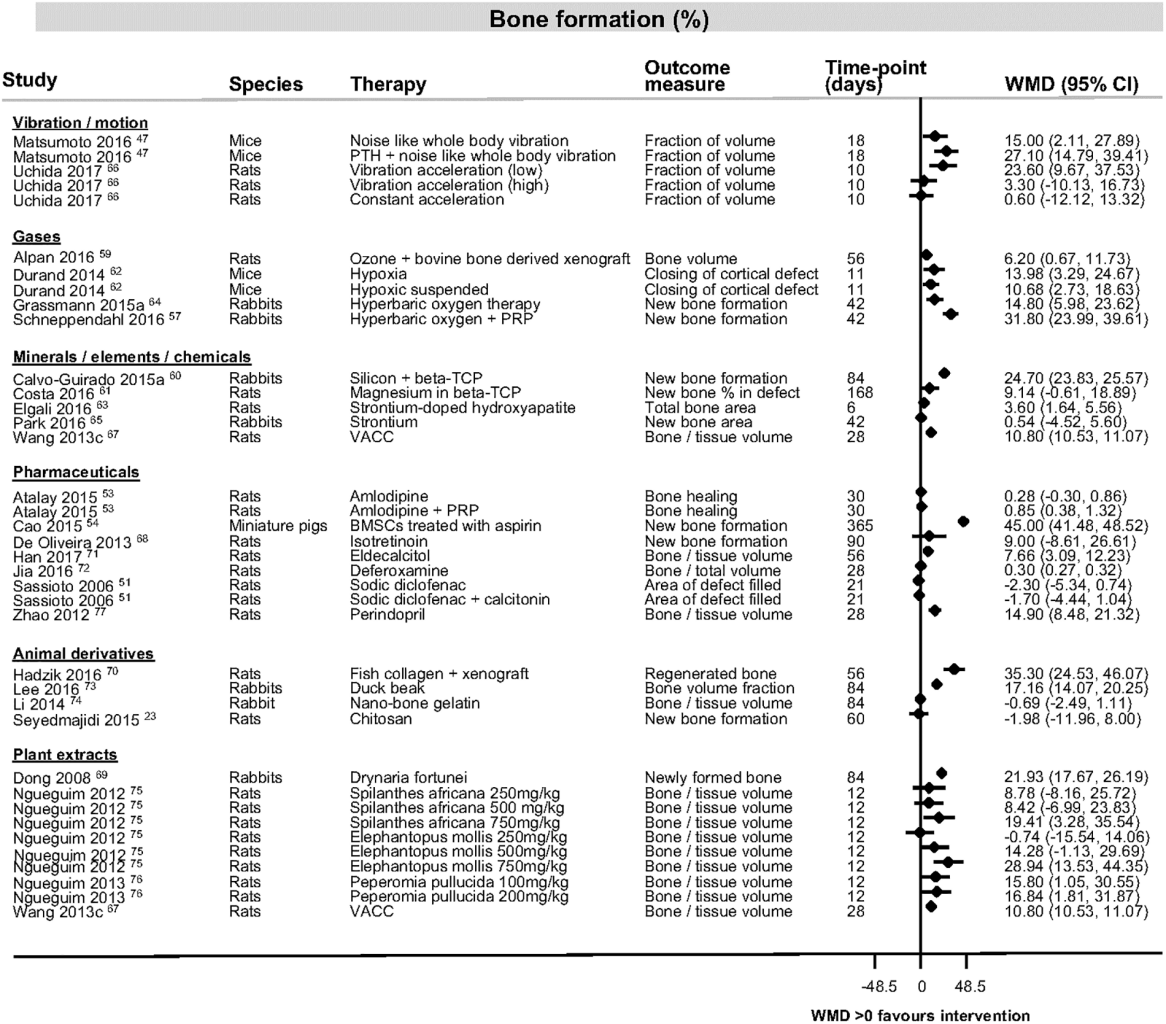
Bone formation data for studies looking at interventions of vibration and motion, gases, minerals, elements and chemicals, pharmaceuticals, animal derivatives or plant extracts. Forest plot illustrating mean difference in percentage of bone formation as measured by different histological or radiological measures. Abbreviations: BMSCs, bone marrow stromal cells; CI, confidence interval; PRP, platelet rich plasma; PTH, parathyroid hormone; VACC, vanadium absorbed by Coprinus comatus; WMD, weighted mean difference.

**Fig 5:**
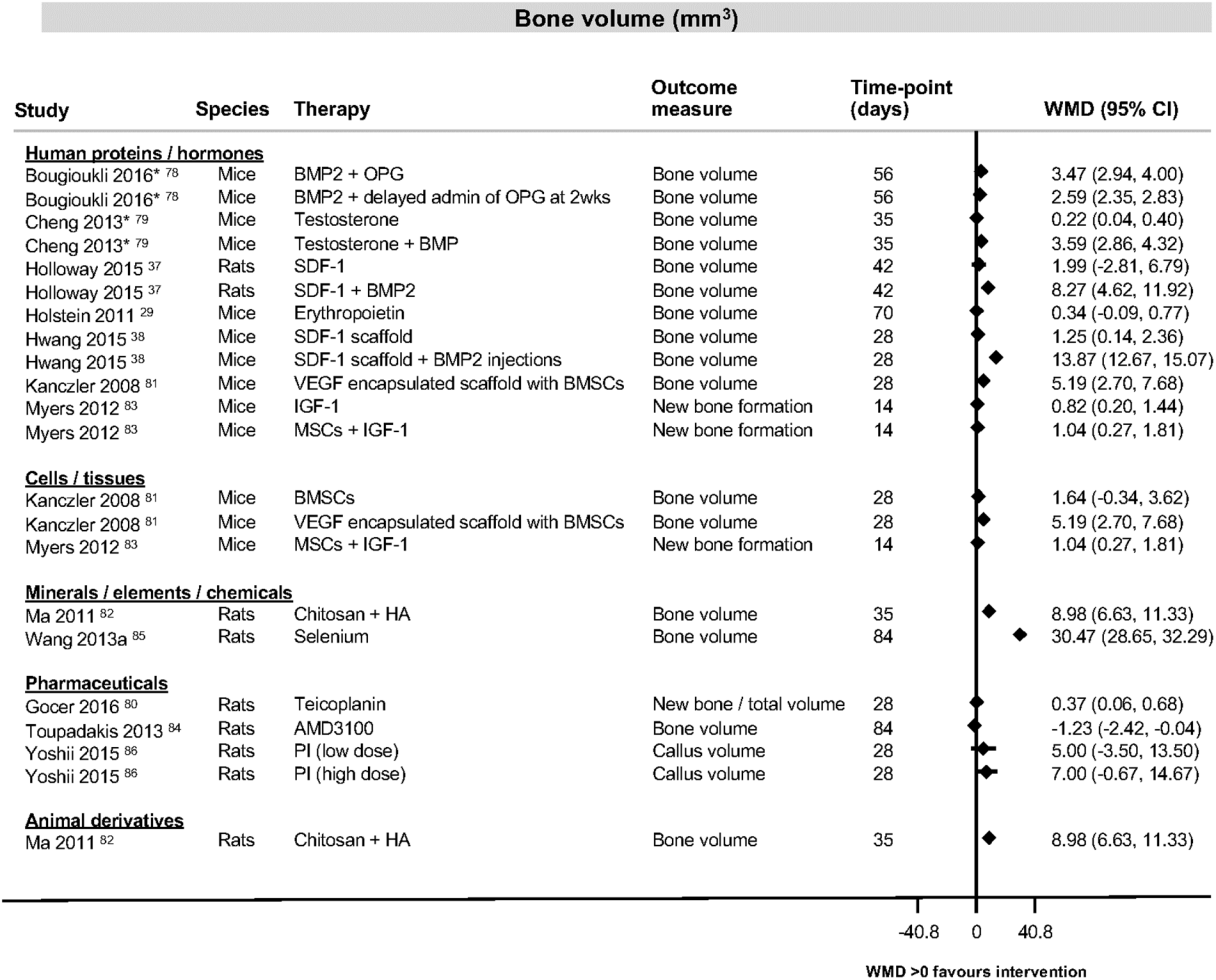
Bone volume data for studies looking at interventions of human proteins and hormones, cells and tissues, minerals, elements and chemicals, pharmaceuticals or animal derivatives. Forest plot illustrating mean difference in cubic millimetre (mm^3^) of bone volume as measured by different histological or radiological measures. *Since none of the control groups healed, the increase in bone volume was set as 0 and the standard deviation as 0.0000001 in order to be able to illustrate those results in a forest plot using STATA. Abbreviations: BMP2, bone morphogenetic protein 2; BMSCs, bone marrow stromal cells; CI, confidence interval; HA, hyaluronic acid; IGF-1, insulin growth factor-1; MSCs, mesenchymal stem cells; OPG, osteoprotegerin; PI, proteasome inhibitor; SDF-1, stromal cell derived factor 1; VEGF, vascular endothelial growth factor; wks, weeks; WMD, weighted mean difference.

**Fig 6:**
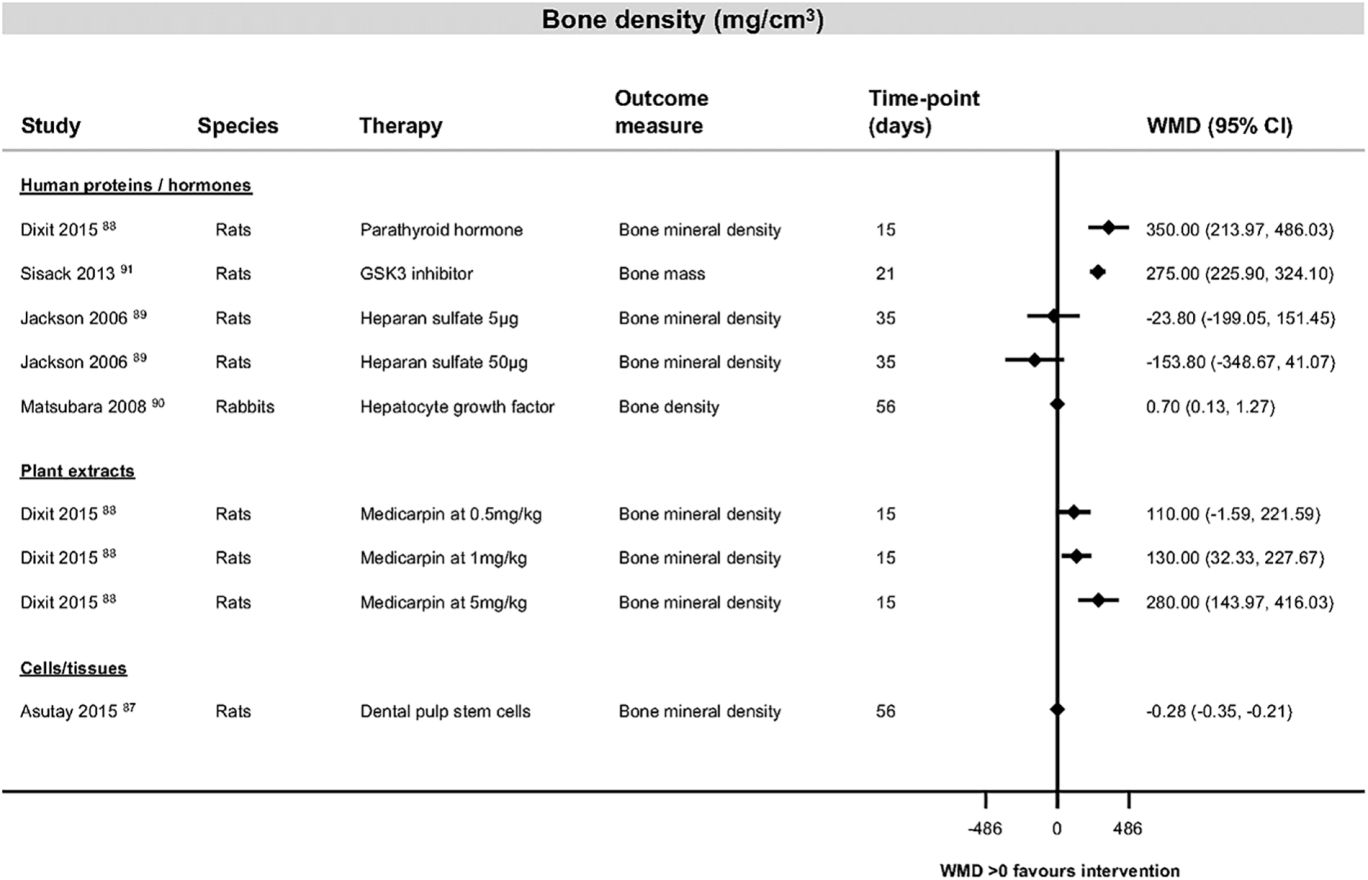
Bone density data for studies looking at interventions of human proteins and hormones, cells and tissues or plant extracts. Forest plot illustrating mean difference in milligrams per cubic centimetre (mg/cm^3^) of bone density as measured by different histological or radiological measures. Abbreviations: CI, confidence interval; GSK3, glycogen synthase kinase 3; WMD, weighted mean difference.

The most consistently reported outcome measure was percentage bone formation in the category of human proteins and hormones (Fig 3 [25, 28, 32, 36, 39–58]). Study findings across all categories for bone formation, bone volume and bone density are shown in Fig 4, [23, 47, 51, 53, 54, 57, 59–77], Fig 5 [29, 37, 38, 78–86] and Fig 6 [87–91]). Table 4 ([92–105]) shows the findings for the pharmaceutical therapies that could not be represented in forest plots, with findings for the remaining categories available as supporting information (S4, S5, S6, S7, S8, S9, S10 and S11 Tables).

**Table 4:** Defect repair data for studies evaluating therapies based on pharmaceuticals (16 therapies, 14 studies).

Study	Therapy	Species	Maximum length of survival (days)	Outcome	Overall effect
Alic 2016 [92]	Cilostazol	Rats	21	No difference between groups at end of 21 days	=
Baht 2017 [93]	Nefopam	Mice	21	Treatment with Nefopam resulted in fracture calluses that contained higher proportions of bone and lower proportions of fibrous tissue	→
Bernick 2014 [94]	Lithium	Rats	28	Fracture healing was maximised with low dose, later onset and longer treatment duration of lithium, resulting in significantly greater yield torque in the therapeutic group	↑
Cai 2015 [95]	Lithium	Rabbits	84	New bone area for lithium containing mesoporous bioglass markedly higher than that for lithium containing bioglass at 56 and 84 days	→
Cakmak 2015 [96]	Pentoxyfylline	Rats	56	No bone growth in control or systemic pentoxyfylline only groups	=
Cakmak 2015 [96]	Pentoxyfylline + iliac crest autograft	Rats	56	Radiological bone union was observed in the iliac crest autograft and systemic pentoxyfylline group compared to no new bone growth in the control group	→
Del Rosario 2015 [97]	Simvastatin	Rats	56	No significant difference between groups	=
Donneys 2013 [98]	Deferoxamine	Rats	40	Greater union rate in treatment group than in irradiated group, but both less than control group	↓
Fan 2017 [99]	Phenamil	Rats	86	Incomplete mandibular restoration was observed in the defect treated with phenamil alone	?
Fan 2017 [99]	Phenamil + BMP	Rats	86	Addition of BMP to phenamil synergistically augmented bone healing, resulting in almost complete bone healing	→
Ishack 2017 [100]	Dipyridamole	Mice	56	Significant increase in percentage of bone regenerated in dipyridamole group compared to control group	↑
Kutan 2016 [101]	Doxycycline	Rats	28	Osteogenesis in the test group was significantly higher than that of the control group	↑
Limirio 2016 [102]	Doxycycline + alendronate	Rats	15	Statistically greater bone density in therapeutic group compared to control group at 15 days	↑
Wada 2013 [103]	Salicylic acid	Rats	84	Significantly higher new bone in defect in therapeutic group compared to control group	↑
Werkman 2006 [104]	Risedronate	Rats	28	No significant difference between therapeutic and control groups	=
Wixted 2009 [105]	Zileuton	Mice	28	Net increase in callus size relative to control	→

^↑^ indicates statistically significant effect on bone formation in trial therapy compared to control

^→^ indicates greater bone formation in trial therapy compared to control, but the effect did not reach statistical significance

^=^ indicates no difference in bone formation rates between the therapeutic or control groups

^↓^ indicates less effect on bone formation in trial therapy compared to control

^?^ indicates results are unclear, and no effect size could be determined

In total 53 human protein and hormone therapy evaluations (30 in forest plots, 23 in tables, 53/100, 53%) reported statistically significant improvements in bone healing compared to the control groups. Statistically significant improvements for the other categories were 50% animal derivatives (16/32), 53% plant extracts (19/36), 55% minerals/elements/chemicals (18/33), 38% pharmaceuticals (11/29), 54% cells/tissues (26/48), 30% vibration/motion (3/10), 100% light/lasers (3/3) and 75% gases (6/8). In total, 135 separate therapy evaluations (135/204, 66%) showed a significantly greater effect on healing of fracture non-union when compared to the control. Only a minority of interventions (9/204, 4%) resulted in significantly less effect on bone union than the comparator arm.

### Meta-analysis

Substantial heterogeneity across studies in terms of type and site of defect, method of defect creation, species, length of follow-up and method of outcome reporting precluded meta-analysis.

**Fig 7:**
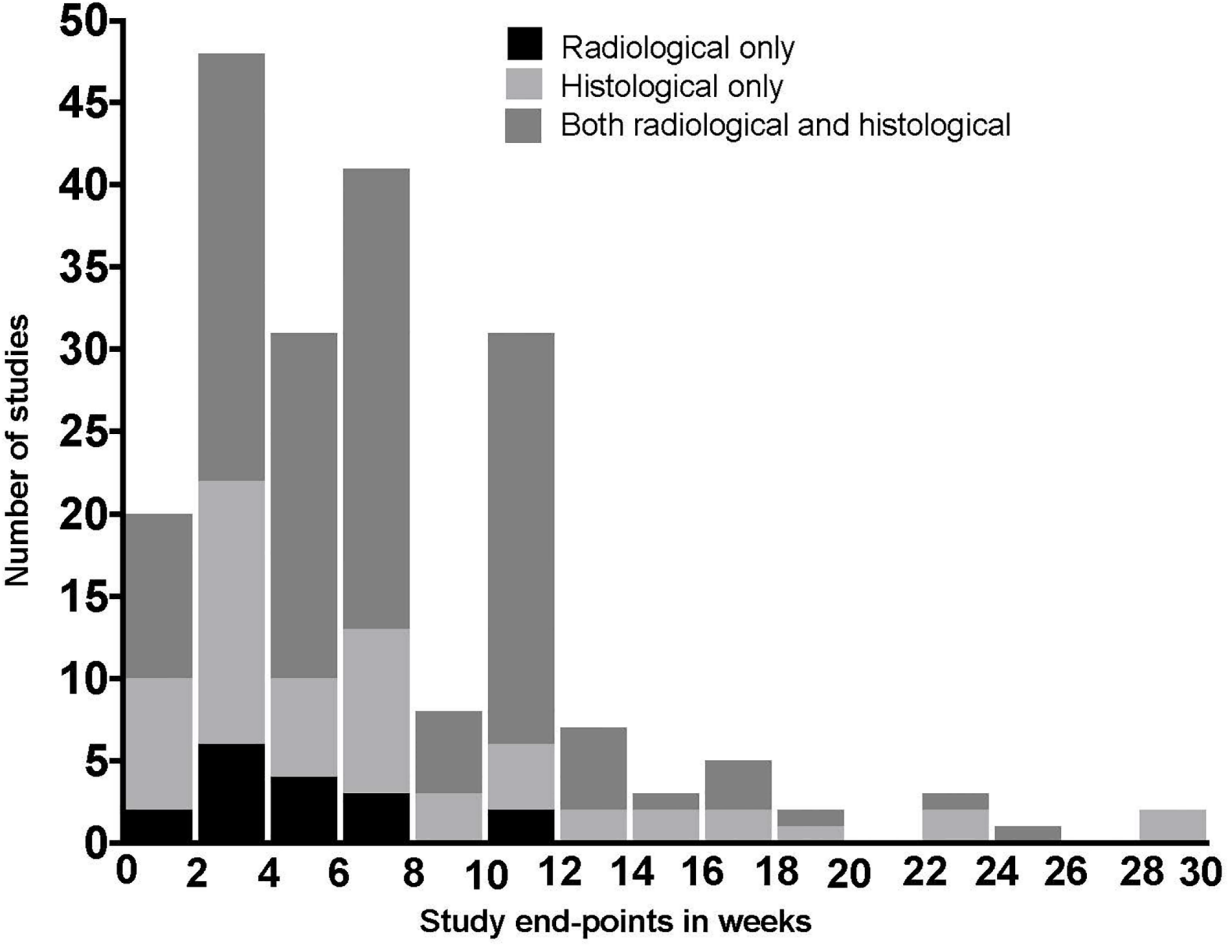
Bar graph demonstrating varied study methodology. Illustration of study-end point in weeks and outcome measure used by all 197 studies.

Rats were the most common animal model, used in 105 studies (105/197, 53%), with the calvarium being the commonest site of bony defect (71/197, 36%). Pigs, dogs, goats, rabbits and mice were also used. Further detail on animal and defect location is given in Table 5. It was not possible to determine the total number of animals used in 28 studies (28/197, 14%) with further detail in S2 Table. Studies used both radiological and histological outcome measures, with follow-up times ranging from 1–30 weeks (Fig 7).

**Table 5:** Model of non-union by species and anatomical location.

	Calvarium	Femur	Humerus	Mandible	Radius	Rib	Tibia	Ulna	Zygomatic arch	Total
Pigs	2									**2**
Dogs					1		1			**2**
Goats							1			**1**
Rabbits	17	8	1	2	14		10	4	1	**57**
Rats	42	40		6	1	1	14	1		**105**
Mice	10	12					8			**30**
**Total**	**71**	**60**	**1**	**8**	**16**	**1**	**34**	**5**	**1**	**197**

Regarding the defect, the majority of studies (75/197, 38%) did not report how the defect was created. A bur was used in 51 studies (51/197, 26%), with other methods including drills (14%), saws (12%), three-point bending (5%), drop weights or pendulums (3%), and being cut with scissors (3%). The defect was explicitly stated as being critical in 75 studies (75/197, 38%) and non-critical in 2 (2/197, 1%), with the remainder of studies (155/197, 79%) not providing this detail. Ten studies (6%) cauterised or stripped the periosteum surrounding the osteotomy.

Only one third of studies (61/197, 31%) included sufficient data to permit illustration in forest plots (without quantitative pooling), due to insufficient reporting of outcome data, or use of less commonly used outcome metrics.

## Discussion

Fracture non-union is a common complication of a common condition [1–3]. This systematic review highlights not only the range of research activity in this field but the poor quality of contemporary animal research investigating this condition. Meta-analysis was not possible due to the diverse and non-standardised nature of the preclinical research, range of outcome measures and poor reporting of results. Despite there being a large amount of data– 204 evaluations across 197 studies—it has not been possible to make a valid comparison between any two studies nor draw firm conclusions regarding relative efficacies from different interventions and therefore identify those therapies that should be prioritised in translational research.

When developing preclinical models of fracture non-union various factors need to be considered. Fundamentally these include the species of animal to be used and the anatomical location of the fracture. Additionally, the type of fracture (transverse or segmental), whether it is subsequently stabilised or not and whether or not the periosteum is stripped are all variables that will affect the union rates of the fracture model. Finally, the delivery method of the therapy under investigation, including the use of scaffolds and carriers, must also be considered. The greater the number of differences that exist between model designs, the less reliably any differences in union rates can be attributed to the therapy under investigation alone, as model variations will act as confounders.

In clinical practice the progression of a fracture to established non-union is multi-factorial, with different types of non-union existing. The majority of primary research contained within this systematic review failed to consider this variability during model development: though the stated aim was to test a therapy designed to prevent or treat non-union, very few used proven models of non-union. The poor fidelity to clinical situations further limits the utility of the preclinical findings.

This systematic review used a methodologically rigorous approach to identifying, selecting and appraising primary studies. There were however some deviations from the previously published protocol; the authors chose to use the MEDLINE version of PUBMED to allow easier duplication of the search strategy on OVID. The decision to limit the systematic review to only these two primary databases was made due to the large volume of eligible studies included. The authors judged it unlikely that the inclusion of a small number of additional studies identified through other sources would significantly alter any conclusions, particularly given the variable and methodologically poor reporting of studies identified in the main databases. Additionally, the large number of studies meant that the risk of bias assessment was performed by one reviewer only for each study.

The studies included in this systematic review were limited by inadequate reporting of methodological details and results. Applying the risk of bias tool developed by SYRCLE showed that many risk of bias criteria were not reported and the rating of ‘unclear’ risk of bias was most common. This in turn hampers interpretation of results. It is however in line with the findings of previous studies which found poor reporting of randomisation procedures and blinding of assessors in animal studies[106], despite multiple resources for study design and reporting available to researchers[107–109]. Some omissions were extremely basic, for example 11% of studies had to be excluded from the forest plots for not stating whether their results were reported as mean with standard deviation, or standard error of the mean, with authors failing to provide clarification when contacted. The use of ± in methodological reporting without further explanation has previously been identified as a persistent problem[110, 111].

To address the problems identified by this review, the authors recommend that the orthopaedic trauma community attempt to reach a consensus on preferred animal models of bone healing similar to the standardisation of fracture classification with the OTA/AO/Muller system[112]. Once a consensus on the standardisation of species, defect and outcome measure is achieved, funding could be restricted to researchers using agreed models and study methodology[113], and journals should similarly restrict publication to studies that would allow direct comparison and insist on reporting results in detail. However, even if this were achieved, the translatability of animal research into effective clinical trials remains controversial [114–116], with even highly cited animal studies failing to translate into successful interventions in clinical trials[117].

This systematic review describes the diverse range of treatments currently under investigation at the preclinical stage for the prevention or treatment of fracture non-union. These therapies can be divided into nine broad treatment categories. Approximately 90% of interventions were only evaluated by a single study, and only five were evaluated three or more times. Reliance on a single study is problematic given the methodological limitations of the research and when considered in the context of publication bias.

Publication bias is an established problem of clinical trials, and its prevalence in animal studies is increasingly recognised[115, 118]. Failing to publish non-significant results of preclinical research limits the ability of researchers to interpret the efficacy of a therapy in the context of the wider literature. It is also unethical: subjecting animals to experiments without publishing the results effectively wastes those animals. The majority of studies included in this review (66%) reported significantly greater rates of bone healing in the therapeutic group compared to the control group. While formal assessment of publication bias was not possible it is reasonable to speculate that a bias against publication of negative or non-significant results persists.

The variability across studies meant that no two studies from the 197 included in this review were judged to be sufficiently similar across clinical and methodological parameters to allow pooling of results in a meta-analysis. Only 31% of studies presented their results in sufficient detail to be illustrated graphically in a forest plot. Not only does this preclude a rapid visual comparison of results from different studies, but it is also indicative of a lack of detail in reporting scientific findings.

Heterogeneity is expected in systematic reviews of preclinical research. Indeed, it could be argued that the aim of a systematic review in this field is to explore and demonstrate the breadth of the evidence, the variability between studies and the consistency of any findings. The generation of a precise pooled effect estimate through meta-analysis even where this is deemed feasible may be of limited value given translatability issues. Yet in this review it was mostly not possible to comment on the consistency of benefit of a particular intervention, as they were mostly only explored in one or two studies.

This systematic review has defined the considerable range of therapies currently being investigated at the preclinical phase for the treatment and prevention of fracture non-union. Though some studies report statistically significant results for some therapies, high levels of clinical and methodological heterogeneity and poor methodological quality and reporting severely hamper the ability to prioritise therapies for translation into clinical trials. If the orthopaedic trauma community were to collectively agree on a standardised animal model for investigating this question, and standards for reporting of all results regardless of findings were mandated, improved clinical treatments for fracture non-union will be developed more efficiently.

## Supporting information

S1 Table PRISMA checklist. Preferred Reporting Items for Systematic Reviews and Meta-Analyses (PRISMA) checklist for reporting of study methodology, results and discussion.
(DOC)

S2 Table Systematic review search strategies for MEDLINE and Embase. (DOCX)

S3 Table Additional study detail. (DOCX)

S4 Table Defect repair data for studies evaluating therapies based on animal derivatives (27 therapies, 18 studies). (DOCX)

S5 Table Defect repair data for studies evaluating therapies based on plant extracts (23 therapies, 23 studies). (DOCX)

S6 Table Defect repair data for studies evaluating therapies based on minerals, elements or chemicals (25 therapies, 21 studies). (DOCX)

S7 Table Defect repair data for studies evaluating therapies based on cells and tissues (32 therapies, 27 studies). (DOCX)

S8 Table Defect repair data for studies evaluating therapies based on vibration or motion (2 therapies, 2 studies). (DOCX)

S9 Table Defect repair data for studies evaluating therapies based on lights or lasers (3 therapies, 2 studies). (DOCX)

S10 Table Defect repair data for studies evaluating therapies based on gases (3 therapies, 3 studies). (DOCX)

S11 Table Defect repair data for studies evaluating therapies based on human proteins or hormones (59 therapies, 42 studies). (DOCX)
