# Supplementary material for: Preclinical therapies to prevent or treat fracture non-union: A systematic review
Source: PLoS One. 2018 Aug 1;13(8):e0201077. doi: 10.1371/journal.pone.0201077 (PMC6070249; doi:10.1371/journal.pone.0201077)
Supplement: S2 Table — (DOCX) [file pone.0201077.s002.docx]

**S2 Table:** Systematic review search strategies for MEDLINE and Embase

Database: Ovid MEDLINE(R) In-Process & Other Non-Indexed Citations and Ovid MEDLINE(R) <1946 to Present>

Search Strategy:

--------------------------------------------------------------------------------

1 exp murinae/

2 (murine or mouse or mice).mp.

3 exp sheep/

4 (sheep or ovine).mp.

5 exp rodentia/

6 (rat$ or rodent$).mp.

7 exp dog/

8 (dog$ or canine).mp.

9 exp cat/

10 (cat$ or feline).mp.

11 exp swine/

12 (pig$ or porcine or swine).mp.

13 exp rabbit/

14 rabbit$.mp.

15 exp goat/

16 (goat$ or caprine).mp.

17 exp primate/ (16888114)

18 (monkey$ or chimp$ or ape).mp.

19 1 or 2 or 3 or 4 or 5 or 6 or 7 or 8 or 9 or 10 or 11 or 12 or 13 or 14 or 15 or 16 or 17 or 18

20 model$.mp.

21 experiment$.mp.

22 trial$.mp.

23 20 or 21 or 22

24 19 and 23

25 (pre-clinical or preclinical).mp. [mp=title, abstract, original title, name of substance word, subject heading word, keyword heading word, protocol supplementary concept word, rare disease supplementary concept word, unique identifier, synonyms]

26 exp models, animal/

27 exp animal experimentation/

28 24 or 25 or 26 or 27

29 exp bone regeneration/

30 (bone regeneration or bony regeneration).mp.

31 (nonunion or non-union).mp.

32 bone union.mp.

33 (bone$ defect or bony defect).mp.

34 (bone$ repair or bony repair).mp.

35 (critical defect or critical size defect or critical-size defect).mp.

36 (non healing defect or non-healing defect).mp.

37 (segment$ defect or non-segmental defect or nonsegmental defect).mp.

38 delay$ union.mp.

39 29 or 30 or 31 or 32 or 33 or 34 or 35 or 36 or 37 or 38

40 28 and 39

41 limit 40 to yr="2004 -Current"

***************************

Database: Embase <1974 to 2017 April 10>

Search Strategy:

--------------------------------------------------------------------------------

1 (murine or mouse or mice).mp.

2 exp Murinae/

3 exp experimental mouse/ or exp mouse/

4 (sheep or ovine).mp.

5 exp sheep/

6 (rat$ or rodent$).mp.

7 exp rat model/ or exp experimental rat/ or exp rat/

8 exp rodent model/ or exp rodent/

9 (dog$ or canine$).mp.

10 exp dog/ or experimental dog/

11 exp canine model/

12 (cat$ or feline$).mp.

13 exp experimental cat/ or exp cat/

14 (pig$ or porcine or swine).mp.

15 exp swine/

16 exp rabbit model/ or exp rabbit/ or exp experimental rabbit/

17 rabbit$.mp.

18 (goat$ or caprine).mp.

19 exp experimental goat/ or exp goat/

20 exp primate/ or exp primate model/

21 (monkey$ or chimp$ or ape$ or primate$).mp.

22 1 or 2 or 3 or 4 or 5 or 6 or 7 or 8 or 9 or 10 or 11 or 12 or 13 or 14 or 15 or 16 or 17 or 18 or 19 or 20 or 21

23 model$.mp.

24 experiment$.mp.

25 trial$.mp.

26 23 or 24 or 25

27 22 and 26

28 (pre-clinical or preclinical).mp.

29 animal model.mp.

30 27 or 28 or 29

31 (non union or non-union).mp.

32 bone union.mp.

33 (bony repair or bone$ repair).mp.

34 (critical size defect or critical defect).mp.

35 (segment$ defect or non-segment$ defect).mp.

36 31 or 32 or 33 or 34 or 35

37 30 and 36

38 limit 37 to yr="2004 -Current"

***************************
