# Supplementary material for: Preclinical therapies to prevent or treat fracture non-union: A systematic review
Source: PLoS One. 2018 Aug 1;13(8):e0201077. doi: 10.1371/journal.pone.0201077 (PMC6070249; doi:10.1371/journal.pone.0201077)
Supplement: S3 Table — (DOCX) [file pone.0201077.s003.docx]

**S3 Table:** Additional study detail

| **Study** | **Species** | **Anatomical location** | **Total number of animals used** | **Stated aim of study** | | | | **Additional Detail** |
| --- | --- | --- | --- | --- | --- | --- | --- | --- |
|  |  |  |  | **Prevent non-union** | **Treat non-union** | **Accelerate healing of bony defect** | **Treat delayed union** |  |
| Ackerson 2014[1] | Mice | Femur | 34 |  |  | 1 |  | S10 Table |
| Alic 2016[2] | Rats | Femur | 60 |  |  | 1 |  | Table 4 |
| Almeida 2007[3] | Rats | Mandible | 50 |  |  | 1 |  | S3 Table |
| Alpan 2016[4] | Rats | Calvarium | 56 |  |  | 1 |  | Figure 4 |
| Asutay 2015[5] | Rats | Calvarium | 15 |  |  | 1 |  | Figure 6 |
| Atalay 2015[6] | Rats | Tibia | 56 |  |  | 1 |  | Figure 3  Figure 4 |
| Ayanoglu 2015[7] | Rabbits | Tibia | 80 |  |  | 1 |  | S6 Table |
| Azavedo 2014[8] | Rabbits | Tibia | 12 |  |  | 1 |  | S3 Table  S5 Table |
| Bae 2016[9] | Mice | Calvarium | 18 |  | 1 |  |  | S5 Table |
| Bahney 2016[10] | Mice | Tibia | 8 |  | 1 |  |  | S6 Table |
| Baht 2017[11] | Mice | Tibia | ? |  |  | 1 |  | Table 4 |
| Bardsley 2017[12] | Rats | Calvarium | ? |  |  | 1 |  | S6 Table |
| Bernick 2014[13] | Rats | Femur | 84 |  |  | 1 |  | Table 4 |
| Bigham-Sadegh 2015[14] | Rabbits | Radius | 15 |  | 1 |  |  | S3 Table |
| Bigham-Sadegh 2017[15] | Rabbits | Radius | 15 |  | 1 |  |  | S3 Table |
| Bougioukli 2016[16] | Mice | Femur | 156 |  | 1 |  |  | Figure 5 |
| Bulbul 2008[17] | Rats | Tibia | 80 |  |  | 1 |  | S3 Table |
| Burim 2016[18] | Rats | Calvarium | 40 |  | 1 |  |  | S4 Table |
| Cai 2008[19] | Rabbits | Calvarium | ? |  |  | 1 |  | S5 Table |
| Cai 2015[20] | Rabbits | Femur | 18 |  |  | 1 |  | Table 4 |
| Cakmak 2015[21] | Rats | Radius | 24 |  | 1 |  |  | S6 Table  Table 4 |
| Calvo-Guirado 2015a[22] | Rabbits | Calvarium | 21 |  | 1 |  |  | Figure 4 |
| Calvo-Guirado 2015b[23] | Rabbits | Tibia | 20 |  |  | 1 |  | Figure 3 |
| Canter 2010[24] | Rats | Calvarium | 240 |  | 1 |  |  | S3 Table  S10 Table |
| Cao 2015[25] | Pigs | Calvarium | 12 |  |  | 1 |  | Figure 3  Figure 4 |
| Casarin 2014[26] | Rats | Calvarium | 30 |  | 1 |  |  | S4 Table |
| Chen 2013a[27] | Rabbits | Calvarium | 12 |  | 1 |  |  | S4 Table |
| Chen 2013b[28] | Rabbits | Ulna | 60 |  | 1 |  |  | S4 Table |
| Chen 2015[29] | Rats | Femur | 18 |  | 1 |  |  | S5 Table |
| Chen 2016[30] | Rats | Tibia | 70 |  | 1 |  |  | Figure 3 |
| Chen 2017[31] | Rabbits | Femur | 24 |  |  | 1 |  | S10 Table |
| Cheng 2013[32] | Mice | Femur | 28 |  | 1 |  |  | Figure 5 |
| Cheng 2016[33] | Rats | Tibia | 60 |  |  | 1 |  | S5 Table |
| Cheung 2010[34] | Rats | Calvarium | ? |  |  | 1 |  | S6 Table |
| Choi 2015[35] | Mice | Calvarium | ? |  | 1 |  |  | S10 Table |
| Costa 2016[36] | Rats | Calvarium | 72 |  | 1 |  |  | Figure 4 |
| Cui 2009[37] | Rabbits | Radius | 80 |  | 1 |  |  | S3 Table |
| Daei-Farshbaf 2014[38] | Rats | Calvarium | 9 |  | 1 |  |  | Figure 3 |
| De Abreu 2016[39] | Rats | Calvarium | 30 |  | 1 |  |  | S4 Table |
| De Mendonca 2006[40] | Rabbits | Zygomatic arch | 24 |  |  | 1 |  | S4 Table |
| De Oliveira 2013[41] | Rats | Calvarium | 30 |  |  | 1 |  | Figure 4 |
| Dedania 2011[42] | Rats | Femur | 29 |  |  | 1 |  | Figure 3 |
| Del Rosario 2015[43] | Rats | Calvarium | 104 |  | 1 |  |  | Table 4 |
| Deng 2008[44] | Rats | Calvarium | 44 |  | 1 |  |  | Figure 3 |
| Dereci 2016[45] | Rats | Calvarium | 30 |  | 1 |  |  | S8 Table |
| Dixit 2015[46] | Rats | Femur | 72 |  |  | 1 |  | Figure 6 |
| Dong 2008[47] | Rabbits | Calvarium | 18 |  |  | 1 |  | Figure 4 |
| Donigan 2012[48] | Rabbits | Tibia | 22 | 1 |  |  |  | S4 Table  S5 Table |
| Donneys 2013[49] | Rats | Mandible | ? |  |  | 1 |  | Table 4 |
| Dosier 2015[50] | Rats | Femur | ? |  | 1 |  |  | S10 Table |
| Duan 2014[51] | Rabbits | Radius | 48 |  |  | 1 |  | Figure 3 |
| Durand 2014[52] | Mice | Femur | 30 |  |  | 1 |  | Figure 4 |
| Durmuslar 2016[53] | Rabbits | Calvarium | 40 |  | 1 |  |  | Figure 3 |
| Elgali 2016[54] | Rats | Femur | 84 |  |  | 1 |  | Figure 4 |
| Ereno 2010[55] | Rabbits | Calvarium | 24 |  | 1 |  |  | S4 Table |
| Ezoddini-Ardakani 2012[56] | Rats | Tibia | 15 |  |  | 1 |  | S3 Table |
| Fan 2016[57] | Rats | Calvarium | 24 |  | 1 |  |  | S6 Table |
| Fan 2017[58] | Rats | Mandible | 42 |  | 1 |  |  | S10 Table  Table 4 |
| Fu 2015[59] | Rats | Calvarium | ? |  | 1 |  |  | S3 Table |
| Fukui 2015[60] | Rats | Femur | 69 |  | 1 |  |  | S6 Table  S10 Table |
| Furuta 2016[61] | Mice | Femur | 77 |  |  | 1 |  | S6 Table |
| Gao 2015[62] | Rats | Femur | 36 |  | 1 |  |  | Figure 3 |
| Garcia 2011[63] | Mice | Femur | 52 |  |  | 1 |  | Figure 3 |
| Ge 2011[64] | Rats | Calvarium | 36 |  | 1 |  |  | S5 Table |
| Geiger 2005[65] | Rabbits | Radius | 60 |  | 1 |  |  | Figure 3 |
| Geiger 2007[66] | Rabbits | Radius | 60 |  | 1 |  |  | Figure 3 |
| Giaversi 2010[67] | Rabbits | Femur | 24 |  | 1 |  |  | S4 Table |
| Glass 2011[68] | Mice | Tibia | ? |  |  | 1 |  | S10 Table |
| Gocer 2016[69] | Rats | Tibia | 20 |  |  | 1 |  | Figure 5 |
| Gordjestani 2006[70] | Rabbits | Calvarium | 30 |  |  | 1 |  | S10 Table |
| Gordjestani 2007[71] | Rabbits | Tibia | 30 |  |  | 1 |  | S10 Table |
| Grassmann 2015a[72] | Rabbits | Radius | 24 |  | 1 |  |  | S9 Table  Figure 4 |
| Grassman 2015b[73] | Rabbits | Radius | 24 |  | 1 |  |  | S6 Table |
| Gunay 2013[74] | Rats | Femur | 56 |  |  | 1 |  | S4 Table |
| Hadzik 2016[75] | Rats | Calvarium | 18 |  |  | 1 |  | Figure 4 |
| Han 2017[76] | Rats | Femur | 64 |  |  | 1 |  | Figure 4 |
| Hao 2016[77] | Rats | Femur | 36 |  | 1 |  |  | S6 Table |
| He 2008[78] | Rabbits | Mandible | 45 |  | 1 |  |  | S5 Table |
| He 2017[79] | Rabbits | Humerus | 40 |  | 1 |  |  | S6 Table  S7 Table |
| Heo 2015[80] | Mice | Calvarium | 8 |  | 1 |  |  | S6 Table  S10 Table |
| Hokugo 2016[81] | Rabbits | Calvarium | 25 |  |  | 1 |  | S5 Table |
| Holloway 2015[82] | Rats | Calvarium | 18 |  | 1 |  |  | Figure 5 |
| Holstein 2011[83] | Mice | Femur | 78 |  | 1 |  |  | Figure 5 |
| Horvathy 2016[84] | Rats | Calvarium | 8 |  | 1 |  |  | Figure 3 |
| Hosaka 2013[85] | Rats | Calvarium | ? |  | 1 |  |  | S3 Table |
| Hreha 2015[86] | Rats | Femur | 108 |  |  | 1 |  | S5 Table |
| Hu 2013[87] | Rats | Calvarium | 22 |  |  | 1 |  | S10 Table |
| Hu 2016[88] | Rats | Tibia | ? |  |  | 1 |  | S5 Table |
| Huh 2009[89] | Rats | Femur | 156 |  |  | 1 |  | S4 Table |
| Hwang 2015[90] | Mice | Calvarium | 36 |  | 1 |  |  | Figure 5 |
| Ishack 2017[91] | Mice | Calvarium | 20 |  | 1 |  |  | Table 4 |
| Issa 2012[92] | Rats | Calvarium | 70 |  | 1 |  |  | S4 Table |
| Jackson 2006[93] | Rats | Femur | 90 |  |  | 1 |  | Figure 6 |
| Jensen 2016[94] | Pigs | Calvarium | 14 |  | 1 |  |  | S6 Table |
| Jia 2014[95] | Rats | Calvarium | 105 |  | 1 |  |  | S10 Table |
| Jia 2016[96] | Rats | Femur | 105 |  | 1 |  |  | Figure 4 |
| Kamolratanakul 2011[97] | Mice | Calvarium | 36 |  | 1 |  |  | S10 Table |
| Kanczler 2008[98] | Mice | Femur | 16 |  | 1 |  |  | Figure 5 |
| Kanda 2015[99] | Rats | Calvarium | 20 |  | 1 |  |  | S3 Table |
| Katsumata 2015[100] | Mice | Calvarium | ? |  |  | 1 |  | S3 Table |
| Khedgikar 2017[101] | Rats | Femur | 32 |  |  | 1 |  | S4 Table |
| Kim 2015a[102] | Rabbits | Calvarium | 9 |  |  | 1 |  | S6 Table |
| Kim 2015b[103] | Rabbits | Calvarium | 9 |  |  | 1 |  | S6 Table |
| Kim 2017a[104] | Rabbits | Calvarium | ? |  |  | 1 |  | S6 Table |
| Kim 2017b[105] | Rabbits | Calvarium | 6 |  |  | 1 |  | S3 Table |
| Koga 2014[106] | Rats | Femur | 120 |  |  | 1 |  | S9 Table |
| Konofaos 2015[107] | Rats | Calvarium | 10 |  | 1 |  |  | Figure 3 |
| Kramer 2008[108] | Rats | Calvarium | 40 |  | 1 |  |  | S10 Table |
| Kutan 2016[109] | Rats | Tibia | 20 |  |  | 1 |  | Table 4 |
| Lattanzi 2008[110] | Rats | Mandible | 36 |  | 1 |  |  | S6 Table |
| Lau 2013[111] | Mice | Tibia | ? |  |  | 1 |  | S10 Table |
| Lee 2015[112] | Mice | Calvarium | ? |  | 1 |  |  | S10 Table |
| Lee 2016[113] | Rabbits | Radius | 48 |  | 1 |  |  | Figure 4 |
| Levy 2016[114] | Mice | Femur | 21 |  | 1 |  |  | S6 Table |
| Li 2009[115] | Rabbits | Tibia | 23 |  | 1 |  |  | Figure 3 |
| Li 2011[116] | Rats | Femur | 24 |  | 1 |  |  | S10 Table |
| Li 2014[117] | Rabbits | Ulna | 45 |  |  | 1 |  | Figure 4 |
| Li 2016[118] | Goats | Tibia | 24 |  |  | 1 |  | S3 Table  S10 Table |
| Li 2017[119] | Mice | Calvarium | ? |  | 1 |  |  | S4 Table |
| Limirio 2016[120] | Rats | Femur | 30 |  |  | 1 |  | Table 4 |
| Lin 2007[121] | Rats | Calvarium | 48 |  | 1 |  |  | S6 Table |
| Lipinsky 2015[122] | Rabbits | Tibia | 14 |  |  | 1 |  | S10 Table |
| Liu 2007[123] | Rats | Tibia | 20 |  |  | 1 |  | S10 Table |
| Liu 2015[124] | Rabbits | Femur | 18 |  |  | 1 |  | S10 Table |
| Lu 2005[125] | Rabbits | Tibia | 72 |  |  | 1 |  | S5 Table |
| Ma 2011[126] | Rats | Calvarium | 18 |  | 1 |  |  | Figure 5 |
| Matsubara 2008[127] | Rabbits | Tibia | 50 |  |  | 1 |  | Figure 6 |
| Matsumoto 2016[128] | Mice | Tibia | 48 |  |  | 1 |  | Figure 3  Figure 4 |
| Medeiros 2013[129] | Rats | Calvarium | 42 |  | 1 |  |  | S4 Table |
| Meimandi Parizi 2015[130] | Rabbits | Radius | 30 |  | 1 |  |  | S3 Table |
| Merolli 2010[131] | Rabbits | Femur | 13 |  | 1 |  |  | S4 Table |
| Montoya 2014[132] | Rats | Calvarium | ? |  | 1 |  |  | S10 Table |
| Morishita 2010[133] | Rats | Femur | 70 |  | 1 |  |  | S10 Table |
| Morse 2016[134] | Rats | Femur | 262 |  |  | 1 |  | S5 Table |
| Myers 2012[135] | Mice | Tibia | 40 |  |  | 1 |  | Figure 5 |
| Nacer 2015[136] | Rats | Femur | 24 |  |  | 1 |  | S5 Table |
| Nam 2016[137] | Rats | Calvarium | 21 |  | 1 |  |  | Figure 3 |
| Nascimento 2010[138] | Rats | Femur | 60 |  |  | 1 |  | S8 Table  S10 Table |
| Ngueguim 2012[139] | Rats | Femur | 24 |  |  | 1 |  | Figure 4 |
| Ngueguim 2013[140] | Rats | Femur | 18 |  |  | 1 |  | Figure 4 |
| Ogilvie 2012[141] | Mice | Tibia | ? |  | 1 |  |  | S10 Table |
| Omlor 2016[142] | Rabbits | Radius | 19 |  | 1 |  |  | S10 Table |
| Ortolan 2014[143] | Rats | Calvarium | 60 |  | 1 |  |  | S5 Table |
| Oryan 2014[144] | Rabbits | Radius | 20 |  | 1 |  |  | S5 Table |
| Park 2008[145] | Rats | Calvarium | 75 |  | 1 |  |  | S3 Table  S5 Table  S6 Table |
| Park 2013[146] | Rats | Femur | 113 |  |  | 1 |  | S10 Table |
| Park 2016[147] | Rabbits | Calvarium | 80 |  |  | 1 |  | Figure 4 |
| Patel 2008[148] | Rats | Calvarium | ? |  | 1 |  |  | S10 Table |
| Peled 2007[149] | Rats | Tibia | ? |  |  | 1 |  | S6 Table |
| Pigossi 2015[150] | Mice | Calvarium | 264 |  | 1 |  |  | Figure 3 |
| Puhar 2016[151] | Rabbits | Calvarium | 2 |  | 1 |  |  | S7 Table |
| Rai 2015[152] | Rabbits | Ulna | 27 |  | 1 |  |  | Figure 3 |
| Rocha 2015[153] | Rats | Femur | 40 |  |  | 1 |  | S9 Table |
| Rozen 2007[154] | Rats | Tibia | ? |  |  | 1 |  | S10 Table |
| Saghiri 2015[155] | Rabbits | Mandible | 90 |  |  | 1 |  | S5 Table |
| Santo 2015[156] | Rats | Ulna | 24 |  | 1 |  |  | S6 Table |
| Sassioto 2006[157] | Rats | Femur | 48 |  |  | 1 |  | Figure 3  Figure 4 |
| Schneppendahl 2016[158] | Rabbits | Radius | 48 |  | 1 |  |  | Figure 3  Figure 4 |
| Serrano 2013[159] | Rats | Calvarium | ? |  | 1 |  |  | S6 Table |
| Servin-Trujillo 2011[160] | Dogs | Tibia | 18 |  |  | 1 |  | S10 Table |
| Seyedmajidi 2015[161] | Rats | Tibia | 32 |  |  | 1 |  | Figure 4 |
| Silva 2015[162] | Rats | Mandible | 42 |  |  | 1 |  | S4 Table |
| Sinha 2009[163] | Rabbits | Ulna | 40 |  |  | 1 |  | S10 Table |
| Sisack 2013[164] | Rats | Femur | 64 |  |  | 1 |  | Figure 6 |
| Song 2011[165] | Rats | Calvarium | 30 |  | 1 |  |  | S6 Table  S10 Table |
| Tang 2017[166] | Rats | Femur | 96 |  | 1 |  |  | S10 Table |
| Tolli 2011[167] | Rats | Femur | 109 |  | 1 |  |  | S3 Table |
| Toupadakis 2013[168] | Rats | Femur | 43 |  |  | 1 |  | Figure 5 |
| Trejo 2010[169] | Rabbits | Femur | ? |  |  | 1 |  | S10 Table |
| Tresguerres 2005[170] | Rabbits | Tibia | 32 |  |  | 1 |  | Figure 3 |
| Uchida 2017[171] | Rats | Rib | 25 |  |  | 1 |  | Figure 4 |
| Ugras 2013[172] | Rats | Tibia | 61 |  |  | 1 |  | S10 Table |
| Wada 2013[173] | Rats | Mandible | 56 |  | 1 |  |  | Table 4 |
| Wan 2014[174] | Mice | Femur | ? |  |  | 1 |  | S10 Table |
| Wang 2013a[175] | Rats | Calvarium | 18 |  |  | 1 |  | Figure 5 |
| Wang 2013b[176] | Rabbits | Femur | 21 |  |  | 1 |  | S4 Table |
| Wang 2013c[177] | Rats | Femur | 45 |  | 1 |  |  | Figure 4 |
| Wang 2014a[178] | Rabbits | Femur | 40 |  |  | 1 |  | S4 Table |
| Wang 2014b[179] | Rats | Femur | 24 |  |  | 1 |  | S4 Table |
| Werkman 2006[180] | Rats | Tibia | 84 |  |  | 1 |  | S5 Table  Table 4 |
| Wixted 2009[181] | Mice | Femur | ? |  |  | 1 |  | S10 Table  Table 4 |
| Wong 2006[182] | Rabbits | Calvarium | 14 |  |  | 1 |  | S4 Table |
| Wong 2008a[183] | Rabbits | Calvarium | 9 |  |  | 1 |  | S4 Table |
| Wong 2008b[184] | Rabbits | Calvarium | 2 |  |  | 1 |  | S4 Table |
| Wong 2013[185] | Rats | Femur | 48 |  |  | 1 |  | S5 Table |
| Woo 2015[186] | Rats | Calvarium | 12 |  | 1 |  |  | S5 Table |
| Woodruff 2007[187] | Rats | Calvarium | ? |  | 1 |  |  | S10 Table |
| Xu 2011[188] | Rats | Femur | 20 |  |  | 1 |  | S10 Table |
| Yoneme 2015[189] | Mice | Tibia | ? |  |  | 1 |  | S3 Table |
| Yoshii 2015[190] | Rats | Femur | 60 |  |  | 1 |  | Figure 5 |
| Young 2009[191] | Rats | Calvarium | 48 |  | 1 |  |  | S10 Table |
| Yu 2015[192] | Dogs | Radius | 20 |  |  | 1 |  | Figure 3 |
| Zanchetta 2012[193] | Rats | Calvarium | 20 |  | 1 |  |  | S6 Table |
| Zhang 2013[194] | Mice | Femur | ? |  |  | 1 |  | S6 Table |
| Zhang 2016[195] | Rats | Femur | 27 |  | 1 |  |  | S6 Table |
| Zhao 2007[196] | Rabbits | Radius | 30 |  |  | 1 |  | S10 Table |
| Zhao 2012[197] | Rats | Tibia | 86 |  |  | 1 |  | Figure 4 |
| **Total** |  |  |  | **1** | **93** | **103** | **0** |  |

? Study did not make clear the total number of animals used

1. Ackerson RM, Shum LC, Berry AR, Bucknell AL, King KB. In vivo model to measure bone repair efficacy of nanoparticle-based drug delivery. Orthopedics. 2014;37(8):e707-11. PubMed PMID: 25102506.

2. Alic T, Cirakli A, Sahin Y, Tomak Y. Effects of cilostazol on fracture healing: an experimental study. Acta Orthop Traumatol Turc. 2016;50(1):103-10. doi: 10.3944/AOTT.2016.15.0211. PubMed PMID: 26854057.

3. Almeida JD, Arisawa EA, da Rocha RF, Carvalho YR. Effect of calcitonin on bone regeneration in male rats: a histomorphometric analysis. International Journal of Oral & Maxillofacial Surgery. 2007;36(5):435-40. PubMed PMID: 17275259.

4. Alpan AL, Toker H, Ozer H. Ozone Therapy Enhances Osseous Healing in Rats With Diabetes With Calvarial Defects: A Morphometric and Immunohistochemical Study. Journal of Periodontology 87(8):982-9, 2016 Aug. PubMed PMID: 26991488.

5. Asutay F, Polat S, Gul M, Subasi C, Kahraman SA, Karaoz E. The effects of dental pulp stem cells on bone regeneration in rat calvarial defect model: micro-computed tomography and histomorphometric analysis. Arch Oral Biol. 2015;60(12):1729-35. doi: 10.1016/j.archoralbio.2015.09.002. PubMed PMID: 26433189.

6. Atalay Y, Bozkurt MF, Gonul Y, Cakmak O, Agacayak KS, Kose I, et al. The effects of amlodipine and platelet rich plasma on bone healing in rats. Drug Des Devel Ther. 2015;9:1973-81. doi: 10.2147/DDDT.S80778. PubMed PMID: 25897207; PubMed Central PMCID: PMCPMC4396585.

7. Ayanoglu S, Esenyel CZ, Adanir O, Dedeoglu S, Imren Y, Esen T. Effects of hyaluronic acid (Hyalonect) on callus formation in rabbits. Acta Orthop Traumatol Turc. 2015;49(3):319-25. doi: 10.3944/AOTT.2015.14.0231. PubMed PMID: 26200413.

8. Azevedo AS, Sa MJ, Fook MV, Neto PI, Sousa OB, Azevedo SS, et al. Use of chitosan and beta-tricalcium phosphate, alone and in combination, for bone healing in rabbits. Journal of Materials Science-Materials in Medicine. 2014;25(2):481-6. PubMed PMID: 24243224.

9. Bae WJ, Auh QS, Kim GT, Moon JH, Kim EC. Effects of sodium tri- and hexameta-phosphate in vitro osteoblastic differentiation in Periodontal Ligament and Osteoblasts, and in vivo bone regeneration. Differentiation. 2016;92(5):257-69. doi: 10.1016/j.diff.2016.04.004. PubMed PMID: 27160630.

10. Bahney CS, Jacobs L, Tamai R, Hu D, Luan TF, Wang M, et al. Promoting Endochondral Bone Repair Using Human Osteoarthritic Articular Chondrocytes. Tissue Engineering - Part A 22 (5-6) (pp 427-435), 2016 Date of Publication: 01 Mar 2016. PubMed PMID: 609233860.

11. Baht GS, Nadesan P, Silkstone D, Alman BA. Pharmacologically targeting beta-catenin for NF1 associated deficiencies in fracture repair. Bone 98 (pp 31-36), 2017 Date of Publication: 01 May 2017. PubMed PMID: 614644474.

12. Bardsley K, Kwarciak A, Freeman C, Brook I, Hatton P, Crawford A. Repair of bone defects in vivo using tissue engineered hypertrophic cartilage grafts produced from nasal chondrocytes. Biomaterials 112:313-323, 2017 Jan. PubMed PMID: 27770634.

13. Bernick J, Wang Y, Sigal IA, Alman BA, Whyne CM, Nam D. Parameters for lithium treatment are critical in its enhancement of fracture-healing in rodents. Journal of Bone & Joint Surgery - American Volume. 2014;96(23):1990-8. PubMed PMID: 25471914.

14. Bigham-Sadegh A, Karimi I, Shadkhast M, Mahdavi MH. Hydroxyapatite and demineralized calf fetal growth plate effects on bone healing in rabbit model. Journal of Orthopaedics & Traumatology 16(2):141-9, 2015 Jun. PubMed PMID: 25308902.

15. Bigham-Sadegh A, Mohamadnia AR, Shahbazkia HR, Khalilifard S. Role of Coral, demineralized calf fetal growth plate, and a combination of the two in healing of bone defects in Rabbits. Trauma Monthly 22 (2) (no pagination), 2017 Article Number: e28275 Date of Publication: March 2017. 2017. PubMed PMID: 615138815.

16. Bougioukli S, Jain A, Sugiyama O, Tinsley BA, Tang AH, Tan MH, et al. Combination therapy with BMP-2 and a systemic RANKL inhibitor enhances bone healing in a mouse critical-sized femoral defect. Bone. 2016;84 (pp 93-103), 2016. Date of Publication:March 01. PubMed PMID: 607521888.

17. Bulbul M, Esenyel CZ, Esenyel M, Ayanoglu S, Bilgic B, Gulmez T. Effects of calcitonin on the biomechanics, histopathology, and radiography of callus formation in rats. Journal of Orthopaedic Science 13 (2) (pp 136-144), 2008 Date of Publication: March 2008. 2008. PubMed PMID: 2008172919.

18. Burim RA, Sendyk DI, Hernandes LS, de Souza DF, Correa L, Deboni MC. Repair of Critical Calvarias Defects With Systemic Epimedium sagittatum Extract. Journal of Craniofacial Surgery 27(3):799-804, 2016 May. PubMed PMID: 26982112.

19. Cai M, Liu X, Shao J, Qi J, Wang J, Zhu Y, et al. OIC-A006 promotes osteogenesis in vitro and in vivo. Pharmazie. 2008;63(10):751-6. PubMed PMID: 18972839.

20. Cai Y, Guo L, Shen H, An X, Jiang H, Ji F, et al. Degradability, bioactivity, and osteogenesis of biocomposite scaffolds of lithium-containing mesoporous bioglass and mPEG-PLGA-b-PLL copolymer. International Journal of Nanomedicine 10:4125-36, 2015. PubMed PMID: 26150718.

21. Cakmak G, Sahin MS, OzdemIr BH, KaradenIz E. Effect of pentoxifylline on healing of segmental bone defects and angiogenesis. Acta orthopaedica et traumatologica turcica 49 (6) (pp 676-682), 2015 Date of Publication: 2015. PubMed PMID: 611671810.

22. Calvo-Guirado JL, Garces M, Delgado-Ruiz RA, Ramirez Fernandez MP, Ferres-Amat E, Romanos GE. Biphasic beta-TCP mixed with silicon increases bone formation in critical site defects in rabbit calvaria. Clinical Oral Implants Research 26(8):891-7, 2015 Aug. PubMed PMID: 24863557.

23. Calvo-Guirado JL, Gomez-Moreno G, Mate-Sanchez JE, Lopez-Mari L, Delgado-Ruiz R, Romanos GE. New bone formation in bone defects after melatonin and porcine bone grafts: experimental study in rabbits. Clinical Oral Implants Research 26(4):399-406, 2015 Apr. PubMed PMID: 24602080.

24. Canter HI, Vargel I, Korkusuz P, Oner F, Gungorduk DB, Cil B, et al. Effect of use of slow release of bone morphogenetic protein-2 and transforming growth factor-Beta-2 in a chitosan gel matrix on cranial bone graft survival in experimental cranial critical size defect model. Annals of Plastic Surgery. 2010;64(3):342-50. PubMed PMID: 20179488.

25. Cao Y, Xiong J, Mei S, Wang F, Zhao Z, Wang S, et al. Aspirin promotes bone marrow mesenchymal stem cell-based calvarial bone regeneration in mini swine. Stem Cell Research and Therapy 6 (1) (no pagination), 2015 Article Number: 210 Date of Publication: 31 Oct 2015. PubMed PMID: 606719389.

26. Casarin RC, Casati MZ, Pimentel SP, Cirano FR, Algayer M, Pires PR, et al. Resveratrol improves bone repair by modulation of bone morphogenetic proteins and osteopontin gene expression in rats. International Journal of Oral & Maxillofacial Surgery. 2014;43(7):900-6. PubMed PMID: 24530035.

27. Chen KY, Lin KC, Chen YS, Yao CH. A novel porous gelatin composite containing naringin for bone repair. Evidence-Based Complementary & Alternative Medicine: eCAM. 2013;2013:283941. PubMed PMID: 23431335.

28. Chen SH, Lei M, Xie XH, Zheng LZ, Yao D, Wang XL, et al. PLGA/TCP composite scaffold incorporating bioactive phytomolecule icaritin for enhancement of bone defect repair in rabbits. Acta Biomaterialia. 2013;9(5):6711-22. PubMed PMID: 23376238.

29. Chen X, Zhao Y, Geng S, Miron RJ, Zhang Q, Wu C, et al. In vivo experimental study on bone regeneration in critical bone defects using PIB nanogels/boron-containing mesoporous bioactive glass composite scaffold. International Journal of Nanomedicine 10:839-46, 2015. PubMed PMID: 25653525.

30. Chen G, Fang T, Qi Y, Yin X, Di T, Feng G, et al. Combined use of mesenchymal stromal cell sheet transplantation and local injection of SDF-1 for bone repair in a rat nonunion model. Cell Transplantation 25 (10) (pp 1801-1817), 2016 Date of Publication: 2016. PubMed PMID: 612827072.

31. Chen J, Liu W, Zhao J, Sun C, Chen J, Hu K, et al. Gelatin microspheres containing calcitonin gene-related peptide or substance P repair bone defects in osteoporotic rabbits. Biotechnology Letters 39(3):465-472, 2017 Mar. PubMed PMID: 27909823.

32. Cheng BH, Chu TMG, Chang C, Kang HY, Huang KE. Testosterone Delivered with a Scaffold Is as Effective as Bone Morphologic Protein-2 in Promoting the Repair of Critical-Size Segmental Defect of Femoral Bone in Mice. PLoS ONE 8 (8) , 2013 Article Number: e70234 Date of Publication: 05 Aug 2013. 2013. PubMed PMID: 2013494431.

33. Cheng H, Xiong W, Fang Z, Guan H, Wu W, Li Y, et al. Strontium (Sr) and silver (Ag) loaded nanotubular structures with combined osteoinductive and antimicrobial activities. Acta Biomaterialia 31:388-400, 2016 Feb. PubMed PMID: 26612413.

34. Cheung WK, Working DM, Galuppo LD, Leach JK. Osteogenic comparison of expanded and uncultured adipose stromal cells. Cytotherapy. 2010;12(4):554-62. PubMed PMID: 20370353.

35. Choi H, Jeong BC, Hur SW, Kim JW, Lee KB, Koh JT. The angiopoietin-1 variant COMP-Ang1 enhances BMP2-induced bone regeneration with recruiting pericytes in critical sized calvarial defects. PLoS ONE 10 (10) (no pagination), 2015 Article Number: e0140502 Date of Publication: 14 Oct 2015. PubMed PMID: 607111800.

36. Costa NM, Yassuda DH, Sader MS, Fernandes GV, Soares GD, Granjeiro JM. Osteogenic effect of tricalcium phosphate substituted by magnesium associated with Genderm membrane in rat calvarial defect model. Materials Science & Engineering C, Materials for Biological Applications 61:63-71, 2016 Apr 01. PubMed PMID: 26838825.

37. Cui X, Zhao D, Zhang B, Gao Y. Osteogenesis mechanism of chitosan-coated calcium sulfate pellets on the restoration of segmental bone defects. Journal of Craniofacial Surgery. 2009;20(5):1445-50. PubMed PMID: 19816276.

38. Daei-farshbaf N, Ardeshirylajimi A, Seyedjafari E, Piryaei A, Fadaei Fathabady F, Hedayati M, et al. Bioceramic-collagen scaffolds loaded with human adipose-tissue derived stem cells for bone tissue engineering. Molecular Biology Reports 41 (2) (pp 741-749), 2014 Date of Publication: February 2014. 2014. PubMed PMID: 2014277648.

39. De Abreu TC, De Lima RP, De Souza VSB, Junior OC, De Albuquerque AV, Aguiar JLA, et al. The biopolymer sugarcane as filling material of critical defects in rats. Acta Cirurgica Brasileira 31 (1) (pp 53-58), 2016 Date of Publication: January 2016. PubMed PMID: 608139565.

40. De Mendonca JCG, De Rossi R, Inouye CM, Bazan DRP, Monteiro JCC, De Mendonca JP. Morphology of autogenous bone graft and castor oil polyurethane in the infraorbital rim of rabbits: A comparative study. Acta Cirurgica Brasileira 21 (5) (pp 341-347), 2006 Date of Publication: September/October 2006. 2006. PubMed PMID: 2006499857.

41. de Oliveira HT, Bergoli RD, Hirsch WD, Chagas OL, Jr., Heitz C, Silva DN. Isotretinoin effect on the repair of bone defects - a study in rat calvaria. Journal of Cranio-Maxillo-Facial Surgery. 2013;41(7):581-5. PubMed PMID: 23273647.

42. Dedania J, Borzio R, Paglia D, Breitbart EA, Mitchell A, Vaidya S, et al. Role of local insulin augmentation upon allograft incorporation in a rat femoral defect model. Journal of Orthopaedic Research. 2011;29(1):92-9. PubMed PMID: 20661933.

43. Del Rosario C, Rodriguez-Evora M, Reyes R, Simoes S, Concheiro A, Evora C, et al. Bone critical defect repair with poloxamine-cyclodextrin supramolecular gels. International Journal of Pharmaceutics 495 (1) (pp 463-473), 2015 Date of Publication: 10 Nov 2015. PubMed PMID: 606064012.

44. Deng F, Ling J, Ma J, Liu C, Zhang W. Stimulation of intramembranous bone repair in rats by ghrelin. Experimental Physiology 93 (7) (pp 872-879), 2008 Date of Publication: July 2008. 2008. PubMed PMID: 2008313191.

45. Dereci O, Sindel A, Serap Toru H, Yuce E, Ay S, Tozoglu S. The Comparison of the Efficacy of Blue Light-Emitting Diode Light and 980-nm Low-Level Laser Light on Bone Regeneration. Journal of Craniofacial Surgery 27(8):2185-2189, 2016 Nov. PubMed PMID: 28005786.

46. Dixit M, Raghuvanshi A, Gupta CP, Kureel J, Mansoori MN, Shukla P, et al. Medicarpin, a Natural Pterocarpan, Heals Cortical Bone Defect by Activation of Notch and Wnt Canonical Signaling Pathways. PLoS ONE 10 (12) (no pagination), 2015 Article Number: e0144541 Date of Publication: 01 Dec 2015. PubMed PMID: 607918196.

47. Dong GC, Chen HM, Yao CH. A novel bone substitute composite composed of tricalcium phosphate, gelatin and drynaria fortunei herbal extract. Journal of Biomedical Materials Research. 2008;Part A. 84(1):167-77. PubMed PMID: 17607749.

48. Donigan JA, Fredericks DC, Nepola JV, Smucker JD. The effect of transdermal nicotine on fracture healing in a rabbit model. Journal of Orthopaedic Trauma. 2012;26(12):724-7. PubMed PMID: 22955337.

49. Donneys A, Weiss DM, Deshpande SS, Ahsan S, Tchanque-Fossuo CN, Sarhaddi D, et al. Localized deferoxamine injection augments vascularity and improves bony union in pathologic fracture healing after radiotherapy. Bone. 2013;52(1):318-25. PubMed PMID: 23085084.

50. Dosier CR, Uhrig BA, Willett NJ, Krishnan L, Li MT, Stevens HY, et al. Effect of cell origin and timing of delivery for stem cell-based bone tissue engineering using biologically functionalized hydrogels. Tissue engineering. 2015;Part A.. 21(1-2):156-65. PubMed PMID: 25010532.

51. Duan C, Liu J, Yuan Z, Meng G, Yang X, Jia S, et al. Adenovirus-mediated transfer of VEGF into marrow stromal cells combined with PLGA/TCP scaffold increases vascularization and promotes bone repair in vivo. Archives of Medical Science 10 (1) (pp 174-181), 2014 Date of Publication: February 2014. 2014. PubMed PMID: 2014171520.

52. Durand M, Collombet JM, Frasca S, Begot L, Lataillade JJ, Le Bousse-Kerdiles MC, et al. In vivo hypobaric hypoxia performed during the remodeling process accelerates bone healing in mice. Stem Cells Translational Medicine. 2014;3(8):958-68. PubMed PMID: 24944208.

53. Durmuslar MC, Balli U, Ongoz Dede F, Bozkurt Dogan S, Misir AF, Baris E, et al. Evaluation of the effects of platelet-rich fibrin on bone regeneration in diabetic rabbits. Journal of Cranio-Maxillo-Facial Surgery 44(2):126-33, 2016 Feb. PubMed PMID: 26732635.

54. Elgali I, Turri A, Xia W, Norlindh B, Johansson A, Dahlin C, et al. Guided bone regeneration using resorbable membrane and different bone substitutes: Early histological and molecular events. Acta Biomaterialia 29:409-23, 2016 Jan. PubMed PMID: 26441123.

55. Ereno C, Guimaraes SAC, Pasetto S, Herculano RD, Silva CP, Graeff CFO, et al. Latex use as an occlusive membrane for guided bone regeneration. Journal of Biomedical Materials Research - Part A 95 (3 A) (pp 932-939), 2010 Date of Publication: 01 Dec 2010. 2010. PubMed PMID: 2010622042.

56. Ezoddini-Ardakani F, Navabazam A, Fatehi F, Danesh-Ardekani M, Khadem S, Rouhi G. Histologic evaluation of chitosan as an accelerator of bone regeneration in microdrilled rat tibias. Dental Research Journal. 2012;9(6):694-9. PubMed PMID: 23559943.

57. Fan D, Liu S, Jiang S, Li Z, Mo X, Ruan H, et al. The use of SHP-2 gene transduced bone marrow mesenchymal stem cells to promote osteogenic differentiation and bone defect repair in rat. Journal of Biomedical Materials Research - Part A (no pagination), 2016 Date of Publication: 2016. PubMed PMID: 609701094.

58. Fan J, Guo M, Im CS, Pi-Anfruns J, Cui ZK, Kim S, et al. Enhanced Mandibular Bone Repair by Combined Treatment of Bone Morphogenetic Protein 2 and Small-Molecule Phenamil. Tissue Engineering - Part A 23 (5-6) (pp 195-207), 2017 Date of Publication: March 2017. PubMed PMID: 614720791.

59. Fu YT, Sheu SY, Chen YS, Chen KY, Yao CH. Porous gelatin/tricalcium phosphate/genipin composites containing lumbrokinase for bone repair. Bone. 2015;78 (pp 15-22), 2015. Date of Publication:September 01. PubMed PMID: 604246391.

60. Fukui T, Mifune Y, Matsumoto T, Shoji T, Kawakami Y, Kawamoto A, et al. Superior Potential of CD34-Positive Cells Compared to Total Mononuclear Cells for Healing of Nonunion Following Bone Fracture. Cell Transplantation 24(7):1379-93, 2015. PubMed PMID: 24800622.

61. Furuta T, Miyaki S, Ishitobi H, Ogura T, Kato Y, Kamei N, et al. Mesenchymal stem cell-derived exosomes promote fracture healing in a mouse model. Stem Cells Translational Medicine 5 (12) (pp 1620-1630), 2016 Date of Publication: 01 Dec 2016. PubMed PMID: 613345525.

62. Gao F, Zhang C, Chai Y, Li X. Lentivirus-mediated Wnt10b overexpression enhances fracture healing in a rat atrophic non-union model. Biotechnology letters 37 (3) (pp 733-739), 2015 Date of Publication: 01 Mar 2015. PubMed PMID: 607473326.

63. Garcia P, Speidel V, Scheuer C, Laschke MW, Holstein JH, Histing T, et al. Low dose erythropoietin stimulates bone healing in mice. Journal of Orthopaedic Research 29 (2) (pp 165-172), 2011 Date of Publication: February 2011. 2011. PubMed PMID: 2011016850.

64. Ge Y, Feng H, Wang L. Application of a novel resorbable membrane in the treatment of calvarial defects in rats. Journal of Biomaterials Science, Polymer Edition 22 (18) (pp 2417-2429), 2011 Date of Publication: 2011. 2011. PubMed PMID: 2011616926.

65. Geiger F, Bertram H, Berger I, Lorenz H, Wall O, Eckhardt C, et al. Vascular endothelial growth factor gene-activated matrix (VEGF 165-GAM) enhances osteogenesis and angiogenesis in large segmental bone defects. Journal of Bone and Mineral Research 20 (11) (pp 2028-2035), 2005 Date of Publication: November 2005. 2005. PubMed PMID: 2005486975.

66. Geiger F, Lorenz H, Xu W, Szalay K, Kasten P, Claes L, et al. VEGF producing bone marrow stromal cells (BMSC) enhance vascularization and resorption of a natural coral bone substitute. Bone. 2007;41(4):516-22. PubMed PMID: 17693148.

67. Giavaresi G, Fini M, Salvage J, Nicoli Aldini N, Giardino R, Ambrosio L, et al. Bone regeneration potential of a soybean-based filler: experimental study in a rabbit cancellous bone defects. Journal of Materials Science-Materials in Medicine. 2010;21(2):615-26. PubMed PMID: 19771493.

68. Glass GE, Chan JK, Freidin A, Feldmann M, Horwood NJ, Nanchahal J. TNF-alpha promotes fracture repair by augmenting the recruitment and differentiation of muscle-derived stromal cells. Proceedings of the National Academy of Sciences of the United States of America. 2011;108(4):1585-90. PubMed PMID: 21209334.

69. Gocer H, Onger ME, Kuyubasi N, Cirakli A, Kir MC. The effect of teicoplanin on fracture healing: an experimental study. Eklem hastaliklari ve cerrahisi = Joint Diseases & Related Surgery 27(1):16-21, 2016. PubMed PMID: 26874630.

70. Gordjestani M, Dermaut L, De Ridder L, De Waele P, De Leersnijder W, Bosman F. Osteopontin and bone metabolism in healing cranial defects in rabbits. International Journal of Oral and Maxillofacial Surgery 35 (12) (pp 1127-1132), 2006 Date of Publication: December 2006. 2006. PubMed PMID: 2006551786.

71. Gordjestani M, Dermaut L, De Ridder L, De Waele P. Osteopontin and bone repair in rabbit tibial defect. European Journal of Orthopaedic Surgery and Traumatology 17 (2) (pp 139-145), 2007 Date of Publication: March 2007. 2007. PubMed PMID: 2007117353.

72. Grassmann JP, Schneppendahl J, Hakimi AR, Herten M, Betsch M, Logters TT, et al. Hyperbaric oxygen therapy improves angiogenesis and bone formation in critical sized diaphyseal defects. Journal of Orthopaedic Research. 2015;33(4):513-20. PubMed PMID: 25640997.

73. Grassmann JP, Schneppendahl J, Sager M, Hakimi AR, Herten M, Loegters TT, et al. The effect of bone marrow concentrate and hyperbaric oxygen therapy on bone repair. Journal of Materials Science-Materials in Medicine. 2015;26(1):5331. PubMed PMID: 25577213.

74. Gunay M, Amanvermez R, Keles G. Ankaferd Blood Stopper: Does it have a role in fracture healing? Turkish Journal of Medical Sciences 43 (5) (pp 733-738), 2013 Date of Publication: 2013. 2013. PubMed PMID: 2013536190.

75. Hadzik J, Kubasiewicz-Ross P, Kunert-Keil C, Jurczyszyn K, Nawrot-Hadzik I, Dominiak M, et al. A silver carp skin derived collagen in bone defect treatment-A histological study in a rat model. Annals of Anatomy 208:123-128, 2016 Nov. PubMed PMID: 27507153.

76. Han X, Du J, Liu D, Liu H, Amizuka N, Li M. Histochemical examination of systemic administration of eldecalcitol combined with guided bone regeneration for bone defect restoration in rats. Journal of Molecular Histology 48(1):41-51, 2017 Feb. PubMed PMID: 27882438.

77. Hao C, Wang Y, Shao L, Liu J, Chen L, Zhao Z. Local Injection of Bone Mesenchymal Stem Cells and Fibrin Glue Promotes the Repair of Bone Atrophic Nonunion In Vivo. Advances in Therapy 33(5):824-33, 2016 May. PubMed PMID: 27098172.

78. He H, Yan W, Chen G, Lu Z. Acceleration of de novo bone formation with a novel bioabsorbable film: a histomorphometric study in vivo. Journal of Oral Pathology & Medicine. 2008;37(6):378-82. PubMed PMID: 18355176.

79. He S, Zhao W, Zhang L, Mi L, Du G, Sun C, et al. Low-frequency vibration treatment of bone marrow stromal cells induces bone repair in vivo. Iranian Journal of Basic Medical Sciences 20 (1) (pp 23-28), 2017 Date of Publication: Janaury 2017. PubMed PMID: 613969184.

80. Heo SC, Shin WC, Lee MJ, Kim BR, Jang IH, Choi EJ, et al. Periostin accelerates bone healing mediated by human mesenchymal stem cell-embedded hydroxyapatite/tricalcium phosphate scaffold. PLoS ONE 10 (3) (no pagination), 2015 Article Number: e0116698 Date of Publication: 16 Mar 2015. PubMed PMID: 603024238.

81. Hokugo A, Sorice S, Parhami F, Yalom A, Li A, Zuk P, et al. A novel oxysterol promotes bone regeneration in rabbit cranial bone defects. Journal Of Tissue Engineering & Regenerative Medicine 10(7):591-9, 2016 Jul. PubMed PMID: 23997014.

82. Holloway JL, Ma H, Rai R, Hankenson KD, Burdick JA. Synergistic Effects of SDF-1alpha and BMP-2 Delivery from Proteolytically Degradable Hyaluronic Acid Hydrogels for Bone Repair. Macromolecular Bioscience 15 (9) (pp 1218-1223), 2015 Date of Publication: 01 Sep 2015. PubMed PMID: 604786143.

83. Holstein JH, Orth M, Scheuer C, Tami A, Becker SC, Garcia P, et al. Erythropoietin stimulates bone formation, cell proliferation, and angiogenesis in a femoral segmental defect model in mice. Bone. 2011;49(5):1037-45. PubMed PMID: 21851867.

84. Horvathy DB, Vacz G, Szabo T, Szigyarto IC, Toro I, Vamos B, et al. Serum albumin coating of demineralized bone matrix results in stronger new bone formation. Journal of Biomedical Materials Research Part B, Applied Biomaterials 104(1):126-32, 2016 Jan. PubMed PMID: 25677203.

85. Hosaka YZ, Iwai Y, Tamura J, Uehara M. Diamond squid (Thysanoteuthis rhombus)-derived chondroitin sulfate stimulates bone healing within a rat calvarial defect. Marine Drugs. 2013;11(12):5024-35. PubMed PMID: 24335526.

86. Hreha J, Wey A, Cunningham C, Krell ES, Brietbart EA, Paglia DN, et al. Local manganese chloride treatment accelerates fracture healing in a rat model. Journal of Orthopaedic Research 33(1):122-30, 2015 Jan. PubMed PMID: 25231276.

87. Hu X, Zhang P, Xu Z, Chen H, Xie X. GPNMB enhances bone regeneration by promoting angiogenesis and osteogenesis: potential role for tissue engineering bone. Journal of Cellular Biochemistry. 2013;114(12):2729-37. PubMed PMID: 23794283.

88. Hu JX, Ran JB, Chen S, Jiang P, Shen XY, Tong H. Carboxylated Agarose (CA)-Silk Fibroin (SF) Dual Confluent Matrices Containing Oriented Hydroxyapatite (HA) Crystals: Biomimetic Organic/Inorganic Composites for Tibia Repair. Biomacromolecules 17 (7) (pp 2437-2447), 2016 Date of Publication: 11 Jul 2016. PubMed PMID: 611180645.

89. Huh JE, Kwon NH, Baek YH, Lee JD, Choi DY, Jingushi S, et al. Formononetin promotes early fracture healing through stimulating angiogenesis by up-regulating VEGFR-2/Flk-1 in a rat fracture model. International Immunopharmacology. 2009;9(12):1357-65. PubMed PMID: 19695348.

90. Hwang HD, Lee JT, Koh JT, Jung HM, Lee HJ, Kwon TG. Sequential Treatment with SDF-1 and BMP-2 Potentiates Bone Formation in Calvarial Defects. Tissue engineering Part A 21(13-14):2125-35, 2015 Jul. PubMed PMID: 25919507.

91. Ishack S, Mediero A, Wilder T, Ricci JL, Cronstein BN. Bone regeneration in critical bone defects using three-dimensionally printed beta-tricalcium phosphate/hydroxyapatite scaffolds is enhanced by coating scaffolds with either dipyridamole or BMP-2. Journal of Biomedical Materials Research - Part B Applied Biomaterials 105 (2) (pp 366-375), 2017 Date of Publication: 01 Feb 2017. PubMed PMID: 607198112.

92. Issa JP, Defino HL, Pereira YC, Netto JC, Sebald W, Bentley MV, et al. Bone repair investigation using rhBMP-2 and angiogenic protein extracted from latex. Microscopy Research & Technique. 2012;75(2):145-52. PubMed PMID: 21761496.

93. Jackson RA, McDonald MM, Nurcombe V, Little DG, Cool SM. The use of heparan sulfate to augment fracture repair in a rat fracture model. Journal of Orthopaedic Research. 2006;24(4):636-44. PubMed PMID: 16514633.

94. Jensen J, Tvedesoe C, Rolfing JH, Foldager CB, Lysdahl H, Kraft DC, et al. Dental pulp-derived stromal cells exhibit a higher osteogenic potency than bone marrow-derived stromal cells in vitro and in a porcine critical-size bone defect model. Sicotj 2:16, 2016 Apr 20. PubMed PMID: 27163105.

95. Jia S, Yang X, Song W, Wang L, Fang K, Hu Z, et al. Incorporation of osteogenic and angiogenic small interfering RNAs into chitosan sponge for bone tissue engineering. International Journal of Nanomedicine. 2014;9:5307-16. PubMed PMID: 25429217.

96. Jia P, Chen H, Kang H, Qi J, Zhao P, Jiang M, et al. Deferoxamine released from poly(lactic-co-glycolic acid) promotes healing of osteoporotic bone defect via enhanced angiogenesis and osteogenesis. Journal of Biomedical Materials Research Part A 104(10):2515-27, 2016 Oct. PubMed PMID: 27227768.

97. Kamolratanakul P, Hayata T, Ezura Y, Kawamata A, Hayashi C, Yamamoto Y, et al. Nanogel-based scaffold delivery of prostaglandin E(2) receptor-specific agonist in combination with a low dose of growth factor heals critical-size bone defects in mice. Arthritis & Rheumatism. 2011;63(4):1021-33. PubMed PMID: 21190246.

98. Kanczler JM, Ginty PJ, Barry JJA, Clarke NMP, Howdle SM, Shakesheff KM, et al. The effect of mesenchymal populations and vascular endothelial growth factor delivered from biodegradable polymer scaffolds on bone formation. Biomaterials 29 (12) (pp 1892-1900), 2008 Date of Publication: April 2008. 2008. PubMed PMID: 2008096449.

99. Kanda N, Anada T, Handa T, Kobayashi K, Ezoe Y, Takahashi T, et al. Orthotopic Osteogenecity Enhanced by a Porous Gelatin Sponge in a Critical-Sized Rat Calvaria Defect. Macromolecular Bioscience 15 (12) (pp 1647-1655), 2015 Date of Publication: 01 Dec 2015. PubMed PMID: 605297923.

100. Katsumata Y, Kajiya H, Okabe K, Fukushima T, Ikebe T. A salmon DNA scaffold promotes osteogenesis through activation of sodium-dependent phosphate cotransporters. Biochemical & Biophysical Research Communications 468(4):622-8, 2015 Dec 25. PubMed PMID: 26551467.

101. Khedgikar V, Kushwaha P, Ahmad N, Gautam J, Kumar P, Maurya R, et al. Ethanolic extract of Dalbergia sissoo promotes rapid regeneration of cortical bone in drill-hole defect model of rat. Biomedicine & Pharmacotherapy 86:16-22, 2017 Feb. PubMed PMID: 27936389.

102. Kim BS, Lee J. Enhanced bone healing by improved fibrin-clot formation via fibrinogen adsorption on biphasic calcium phosphate granules. Clinical Oral Implants Research 26(10):1203-10, 2015 Oct. PubMed PMID: 24888232.

103. Kim BS, Kim HJ, Choi JG, You HK, Lee J. The effects of fibrinogen concentration on fibrin/atelocollagen composite gel: an in vitro and in vivo study in rabbit calvarial bone defect. Clinical Oral Implants Research 26(11):1302-8, 2015 Nov. PubMed PMID: 25039258.

104. Kim BS, Yang SS, Lee J. Precoating of biphasic calcium phosphate bone substitute with atelocollagen enhances bone regeneration through stimulation of osteoclast activation and angiogenesis. Journal of Biomedical Materials Research Part A 105(5):1446-1456, 2017 May. PubMed PMID: 28177580.

105. Kim BS, Yang SS, Yoon JH, Lee J. Enhanced bone regeneration by silicon-substituted hydroxyapatite derived from cuttlefish bone. Clinical Oral Implants Research 28(1):49-56, 2017 Jan. PubMed PMID: 26073102.

106. Koga T, Niikura T, Lee SY, Okumachi E, Ueha T, Iwakura T, et al. Topical cutaneous CO2 application by means of a novel hydrogel accelerates fracture repair in rats. Journal of Bone & Joint Surgery - American Volume. 2014;96(24):2077-84. PubMed PMID: 25520342.

107. Konofaos P, Petersen D, Jennings JA, Smith RA, Doty H, Reves BT, et al. Evaluation of Amniotic Multipotential Tissue Matrix to Augment Healing of Demineralized Bone Matrix in an Animal Calvarial Model. Journal of Craniofacial Surgery 26(4):1408-12, 2015 Jun. PubMed PMID: 26080207.

108. Kramer FJ, Meyer M, Morgan D, Forssmann WG, Standker L, Schliephake H, et al. Tissue inhibitor of metalloproteinases II (TIMP-2) is an osteoanabolic factor in vitro and in vivo. European Journal of Medical Research 13 (6) (pp 292-298), 2008 Date of Publication: 24 Jun 2008. 2008. PubMed PMID: 2008340676.

109. Kutan E, Duygu-Capar G, Ozcakir-Tomruk C, Dilek OC, Ozen F, Erdogan O, et al. Efficacy of doxycycline release collagen membrane on surgically created and contaminated defects in rat tibiae: A histopathological and microbiological study. Archives of Oral Biology 63:15-21, 2016 Mar. PubMed PMID: 26658367.

110. Lattanzi W, Parrilla C, Fetoni A, Logroscino G, Straface G, Pecorini G, et al. Ex vivo-transduced autologous skin fibroblasts expressing human Lim mineralization protein-3 efficiently form new bone in animal models. Gene Therapy. 2008;15(19):1330-43. PubMed PMID: 18633445.

111. Lau KH, Kothari V, Das A, Zhang XB, Baylink DJ. Cellular and molecular mechanisms of accelerated fracture healing by COX2 gene therapy: studies in a mouse model of multiple fractures. Bone. 2013;53(2):369-81. PubMed PMID: 23314071.

112. Lee JH, Lee YJ, Cho HJ, Kim DW, Shin H. The incorporation of bFGF mediated by heparin into PCL/gelatin composite fiber meshes for guided bone regeneration. Drug Delivery & Translational Research. 2015;5(2):146-59. PubMed PMID: 25787740.

113. Lee JY, Son SJ, Son JS, Kang SS, Choi SH. Bone-healing capacity of PCL/PLGA/duck beak scaffold in critical bone defects in a rabbit model. BioMed Research International 2016 (no pagination), 2016 Article Number: 2136215 Date of Publication: 2016. PubMed PMID: 609152043.

114. Levy S, Feduska JM, Sawant A, Gilbert SR, Hensel JA, Ponnazhagan S. Immature myeloid cells are critical for enhancing bone fracture healing through angiogenic cascade. Bone 93 (pp 113-124), 2016 Date of Publication: 01 Dec 2016. PubMed PMID: 612349583.

115. Li R, Stewart DJ, Von Schroeder HP, Mackinnon ES, Schemitsch EH. Effect of cell-based VEGF gene therapy on healing of a segmental bone defect. Journal of Orthopaedic Research 27 (1) (pp 8-14), 2009 Date of Publication: January 2009. 2009. PubMed PMID: 2009012823.

116. Li W, Zara JN, Siu RK, Lee M, Aghaloo T, Zhang X, et al. Nell-1 enhances bone regeneration in a rat critical-sized femoral segmental defect model. Plastic & Reconstructive Surgery. 2011;127(2):580-7. PubMed PMID: 21285762.

117. Li W, Zhao Z, Xiong J, Zeng Y. The modification experimental study in vivo of nano-bone gelatin. Artificial Cells, Nanomedicine, & Biotechnology. 2014;42(5):309-15. PubMed PMID: 23899020.

118. Li D, Deng L, Xie X, Yang Z, Kang P. Evaluation of the osteogenesis and angiogenesis effects of erythropoietin and the efficacy of deproteinized bovine bone/recombinant human erythropoietin scaffold on bone defect repair. Journal of Materials Science-Materials in Medicine 27(6):101, 2016 Jun. PubMed PMID: 27091043.

119. Li M, Gu Q, Chen M, Zhang C, Chen S, Zhao J. Controlled delivery of icariin on small intestine submucosa for bone tissue engineering. Materials Science & Engineering C, Materials for Biological Applications 71:260-267, 2017 Feb 01. PubMed PMID: 27987707.

120. Limirio PHJO, Rocha FS, Batista JD, Guimaraes-Henriques JC, de Melo GB, Dechichi P. The Effect of Local Delivery Doxycycline and Alendronate on Bone Repair. AAPS PharmSciTech 17 (4) (pp 872-877), 2016 Date of Publication: 01 Aug 2016. PubMed PMID: 606044038.

121. Lin Y, Wang T, Wu L, Jing W, Chen X, Li Z, et al. Ectopic and in situ bone formation of adipose tissue-derived stromal cells in biphasic calcium phosphate nanocomposite. Journal of Biomedical Materials Research. 2007;Part A. 81(4):900-10. PubMed PMID: 17236222.

122. Lipinsky PV, Sirotin IV, Skoroglyadov AV, Ivkov AV, Oettinger AP, Krynetskiy EE, et al. Effects of prostaglandin E1 on callus formation in rabbits. BMC Musculoskeletal Disorders 16:247, 2015 Sep 10. PubMed PMID: 26359236.

123. Liu ZD, Zhong JL, Xu Y, Miao J. Recombinant human fibroblastic growth factor-2 with soluble tumor necrosis factor receptor-1 facilitates fracture repair in rats with type 2 diabetes mellitus. Journal of Clinical Rehabilitative Tissue Engineering Research 11 (32) (pp 6505-6508), 2007 Date of Publication: 12 Aug 2007. 2007. PubMed PMID: 2007458058.

124. Liu P, Guo L, Huang L, Zhao D, Zhen R, Hu X, et al. Effect of semisynthetic extracellular matrix-like hydrogel containing hepatocyte growth factor on repair of femoral neck defect in rabbits. International journal of clinical and experimental medicine 8(5):7374-80, 2015. PubMed PMID: 26221278.

125. Lu B, Tu ZQ, Pei FX, Liu L. Octyl-a-cyanoacrylate adhesive in the treatment of tibial transverse fracture in rabbits. Chinese Journal of Traumatology. 2005;8(4):240-4. PubMed PMID: 16042872.

126. Ma X, Wang Y, Guo H, Wang J. Nano-hydroxyapatite/chitosan sponge-like biocomposite for repairing of rat calvarial critical-sized bone defect. Journal of Bioactive and Compatible Polymers 26 (4) (pp 335-346), 2011 Date of Publication: July 2011. 2011. PubMed PMID: 2011410088.

127. Matsubara H, Tsuchiya H, Watanabe K, Takeuchi A, Tomita K. Percutaneous nonviral delivery of hepatocyte growth factor in an osteotomy gap promotes bone repair in rabbits: A preliminary study. Clinical Orthopaedics and Related Research 466 (12) (pp 2962-2972), 2008 Date of Publication: December 2008. 2008. PubMed PMID: 2009143232.

128. Matsumoto T, Sato D, Hashimoto Y. Individual and combined effects of noise-like whole-body vibration and parathyroid hormone treatment on bone defect repair in ovariectomized mice. Proceedings of the Institution of Mechanical Engineers Part H, Journal of engineering in medicine 230 (1) (pp 30-38), 2016 Date of Publication: 01 Jan 2016. PubMed PMID: 611950248.

129. Medeiros Junior MD, Carvalho EJ, Catunda IS, Bernardino-Araujo S, Aguiar JL. Hydrogel of polysaccharide of sugarcane molasses as carrier of bone morphogenetic protein in the reconstruction of critical bone defects in rats. Acta Cirurgica Brasileira. 2013;28(4):233-8. PubMed PMID: 23568229.

130. Meimandi Parizi A, Oryan A, Haddadi S, Bigham Sadegh A. Histopathological and biomechanical evaluation of bone healing properties of DBM and DBM-G90 in a rabbit model. Acta orthopaedica et traumatologica turcica 49 (6) (pp 683-689), 2015 Date of Publication: 2015. PubMed PMID: 611671838.

131. Merolli A, Nicolais L, Ambrosio L, Santin M. A degradable soybean-based biomaterial used effectively as a bone filler in vivo in a rabbit. Biomedical Materials. 2010;5(1):15008. PubMed PMID: 20124667.

132. Montoya G, Arenas J, Romo E, Zeichner-David M, Alvarez M, Narayanan AS, et al. Human recombinant cementum attachment protein (hrPTPLa/CAP) promotes hydroxyapatite crystal formation in vitro and bone healing in vivo. Bone. 2014;69 (pp 154-164), 2014. Date of Publication:December 01. PubMed PMID: 2014850110.

133. Morishita Y, Naito M, Miyazaki M, He W, Wu G, Wei F, et al. Enhanced effects of BMP-binding peptide combined with recombinant human BMP-2 on the healing of a rodent segmental femoral defect. Journal of Orthopaedic Research. 2010;28(2):258-64. PubMed PMID: 19639633.

134. Morse A, Cheng TL, Peacock L, Mikulec K, Little DG, Schindeler A. RAP‐011 augments callus formation in closed fractures in rats. Journal of Orthopaedic Research. 2016;34(2):320-30.

135. Myers TJ, Yan Y, Granero-Molto F, Weis JA, Longobardi L, Li T, et al. Systemically delivered insulin-like growth factor-I enhances mesenchymal stem cell-dependent fracture healing. Growth Factors 30 (4) (pp 230-241), 2012 Date of Publication: August 2012. 2012. PubMed PMID: 2012533309.

136. Nacer RS, Silva BA, Poppi RR, Silva DK, Cardoso VS, Delben JR, et al. Biocompatibility and osteogenesis of the castor bean polymer doped with silica (SiO2) or barium titanate (BaTiO3) nanoparticles. Acta Cirurgica Brasileira 30(4):255-63, 2015 Apr. PubMed PMID: 25923258.

137. Nam SS, Lee JC, Kim HJ, Park JW, Lee JM, Suh JY, et al. Serotonin Inhibits Osteoblast Differentiation and Bone Regeneration in Rats. Journal of Periodontology 87(4):461-9, 2016 Apr. PubMed PMID: 26693696.

138. Nascimento SB, Cardoso CA, Ribeiro TP, Almeida JD, Albertini R, Munin E, et al. Effect of low-level laser therapy and calcitonin on bone repair in castrated rats: a densitometric study. Photomedicine and Laser Surgery. 2010;28(1):45-9. PubMed PMID: 19712023.

139. Ngueguim FT, Khan MP, Donfack JH, Siddiqui JA, Tewari D, Nagar GK, et al. Evaluation of Cameroonian plants towards experimental bone regeneration. Journal of Ethnopharmacology. 2012;141(1):331-7. PubMed PMID: 22414477.

140. Ngueguim FT, Khan MP, Donfack JH, Tewari D, Dimo T, Kamtchouing P, et al. Ethanol extract of Peperomia pellucida (Piperaceae) promotes fracture healing by an anabolic effect on osteoblasts. Journal of Ethnopharmacology. 2013;148(1):62-8. PubMed PMID: 23578859.

141. Ogilvie CM, Lu C, Marcucio R, Lee M, Thompson Z, Hu D, et al. Vascular endothelial growth factor improves bone repair in a murine nonunion model. Iowa Orthopaedic Journal. 2012;32:90-4. PubMed PMID: 23576927.

142. Omlor GW, Kleinschmidt K, Gantz S, Speicher A, Guehring T, Richter W. Increased bone formation in a rabbit long-bone defect model after single local and single systemic application of erythropoietin. Acta Orthopaedica 87 (4) (pp 425-431), 2016 Date of Publication: 03 Jul 2016. PubMed PMID: 610983853.

143. Ortolan XR, Fenner BP, Mezadri TJ, Tames DR, Correa R, de Campos Buzzi F. Osteogenic potential of a chalcone in a critical-size defect in rat calvaria bone. Journal of Cranio-Maxillo-Facial Surgery. 2014;42(5):520-4. PubMed PMID: 24041609.

144. Oryan A, Bigham-Sadegh A, Abbasi-Teshnizi F. Effects of osteogenic medium on healing of the experimental critical bone defect in a rabbit model. Bone. 2014;63:53-60. PubMed PMID: 24582803.

145. Park SS, Kim SG, Lim SC, Ong JL. Osteogenic activity of the mixture of chitosan and particulate dentin. Journal of Biomedical Materials Research. 2008;Part A. 87(3):618-23. PubMed PMID: 18186071.

146. Park AG, Paglia DN, Al-Zube L, Hreha J, Vaidya S, Breitbart E, et al. Local insulin therapy affects fracture healing in a rat model. Journal of Orthopaedic Research. 2013;31(5):776-82. PubMed PMID: 23238765.

147. Park JW, Kang DG, Hanawa T. New bone formation induced by surface strontium-modified ceramic bone graft substitute. Oral Diseases 22(1):53-61, 2016 Jan. PubMed PMID: 26458092.

148. Patel ZS, Young S, Tabata Y, Jansen JA, Wong ME, Mikos AG. Dual delivery of an angiogenic and an osteogenic growth factor for bone regeneration in a critical size defect model. Bone. 2008;43(5):931-40. PubMed PMID: 18675385.

149. Peled E, Boss J, Bejar J, Zinman C, Seliktar D. A novel poly(ethylene glycol)-fibrinogen hydrogel for tibial segmental defect repair in a rat model. Journal of Biomedical Materials Research. 2007;Part A. 80(4):874-84. PubMed PMID: 17072852.

150. Pigossi SC, de Oliveira GJ, Finoti LS, Nepomuceno R, Spolidorio LC, Rossa C, Jr., et al. Bacterial cellulose-hydroxyapatite composites with osteogenic growth peptide (OGP) or pentapeptide OGP on bone regeneration in critical-size calvarial defect model. Journal of Biomedical Materials Research Part A 103(10):3397-406, 2015 Oct. PubMed PMID: 25850694.

151. Puhar I, Ma L, Suleimenova D, Chronopoulos V, Mattheos N. The effect of local application of low-magnitude high-frequency vibration on the bone healing of rabbit calvarial defects-a pilot study. Journal of Orthopaedic Surgery 11(1):159, 2016 Dec 08. PubMed PMID: 27931261.

152. Rai B, Chatterjea A, Lim ZXH, Tan TC, Sawyer AA, Hosaka YZ, et al. Repair of segmental ulna defects using a beta-TCP implant in combination with a heparan sulfate glycosaminoglycan variant. Acta Biomaterialia 28 (pp 193-204), 2015 Date of Publication: December 2015. PubMed PMID: 606677876.

153. Rocha FS, Gomes Moura CC, Rocha Rodrigues DB, Zanetta-Barbosa D, Nakamura Hiraki KR, Dechichi P. Influence of hyperbaric oxygen on the initial stages of bone healing. Oral surgery, oral medicine, oral pathology and oral radiology 120 (5) (pp 581-587), 2015 Date of Publication: 01 Nov 2015. PubMed PMID: 612427975.

154. Rozen N, Lewinson D, Bick T, Jacob ZC, Stein H, Soudry M. Fracture repair: modulation of fracture-callus and mechanical properties by sequential application of IL-6 following PTH 1-34 or PTH 28-48. Bone. 2007;41(3):437-45. PubMed PMID: 17599848.

155. Saghiri MA, Orangi J, Tanideh N, Asatourian A, Janghorban K, Garcia-Godoy F, et al. Repair of bone defect by nano-modified white mineral trioxide aggregates in rabbit: A histopathological study. Medicina Oral, Patologia Oral y Cirugia Bucal 20(5):e525-31, 2015 Sep 01. PubMed PMID: 26034924.

156. Santo VE, Ratanavaraporn J, Sato K, Gomes ME, Mano JF, Reis RL, et al. Cell engineering by the internalization of bioinstructive micelles for enhanced bone regeneration. Nanomedicine. 2015;10(11):1707-21.

157. Sassioto MC, Inouye CM, Aydos RD, Figueiredo AS. Bone repair in rats treated with sodic diclofenac and calcitonin. Acta Cirurgica Brasileira. 2006;21 Suppl 4:40-4. PubMed PMID: 17293965.

158. Schneppendahl J, Jungbluth P, Sager M, Benga L, Herten M, Scholz A, et al. Synergistic effects of HBO and PRP improve bone regeneration with autologous bone grafting. Injury 47 (12) (pp 2718-2725), 2016 Date of Publication: 01 Dec 2016. PubMed PMID: 613649381.

159. Serrano J, Romo E, Bermudez M, Narayanan AS, Zeichner-David M, Santos L, et al. Bone regeneration in rat cranium critical-size defects induced by Cementum Protein 1 (CEMP1). PLoS ONE [Electronic Resource]. 2013;8(11):e78807. PubMed PMID: 24265720.

160. Servin-Trujillo MA, Reyes-Esparza JA, Garrido-Farina G, Flores-Gazca E, Osuna-Martinez U, Rodriguez-Fragoso L. Use of a graft of demineralized bone matrix along with TGF-beta1 leads to an early bone repair in dogs. Journal of Veterinary Medical Science. 2011;73(9):1151-61. PubMed PMID: 21566397.

161. Seyedmajidi M, Rabiee S, Haghanifar S, Seyedmajidi S, Jorsaraei SGA, Alaghehmand H, et al. Histopathological, histomorphometrical, and radiographical evaluation of injectable glass-ceramic-chitosan nanocomposite in bone reconstruction of rat. International Journal of Biomaterials 2015 , 2015 Article Number: 719574 Date of Publication: 2015. 2015. PubMed PMID: 2015804875.

162. Silva PF, Brito MV, Pontes FS, Ramos SR, Mendes LC, Oliveira LC. Copaiba oil effect on experimental jaw defect in Wistar rats. Acta Cirurgica Brasileira. 2015;30(2):120-6. PubMed PMID: 25714691.

163. Sinha S, Goel SC. Effect of amino acids lysine and arginine on fracture healing in rabbits: A radiological and histomorphological analysis. Indian Journal of Orthopaedics. 2009;43(4):328-34. PubMed PMID: 19838381.

164. Sisask G, Marsell R, Sundgren-Andersson A, Larsson S, Nilsson O, Ljunggren O, et al. Rats treated with AZD2858, a GSK3 inhibitor, heal fractures rapidly without endochondral bone formation. Bone 54 (1) (pp 126-132), 2013 Date of Publication: May 2013. 2013. PubMed PMID: 2013131892.

165. Song K, Rao NJ, Chen ML, Huang ZJ, Cao YG. Enhanced bone regeneration with sequential delivery of basic fibroblast growth factor and sonic hedgehog. Injury. 2011;42(8):796-802. PubMed PMID: 21367413.

166. Tang Q, Chen LL, Wei F, Sun WL, Lei LH, Ding PH, et al. Effect of 15-Deoxy-DELTA<sup>12,14</sup>-prostaglandin J<sub>2</sub>Nanocapsules on Inflammation and Bone Regeneration in a Rat Bone Defect Model. Chinese Medical Journal 130(3):347-356, 2017 5th Feb. PubMed PMID: 28139520.

167. Tolli H, Kujala S, Jamsa T, Jalovaara P. Reindeer bone extract can heal the critical-size rat femur defect. International Orthopaedics. 2011;35(4):615-22. PubMed PMID: 20454894.

168. Toupadakis CA, Granick JL, Sagy M, Wong A, Ghassemi E, Chung DJ, et al. Mobilization of endogenous stem cell populations enhances fracture healing in a murine femoral fracture model. Cytotherapy. 2013;15(9):1136-47. PubMed PMID: 23831362.

169. Trejo CG, Lozano D, Manzano M, Doadrio JC, Salinas AJ, Dapia S, et al. The osteoinductive properties of mesoporous silicate coated with osteostatin in a rabbit femur cavity defect model. Biomaterials. 2010;31(33):8564-73. PubMed PMID: 20727584.

170. Tresguerres IF, Alobera MA, Baca R, Tresguerres JA. Histologic, morphometric, and densitometric study of peri-implant bone in rabbits with local administration of growth hormone. International Journal of Oral & Maxillofacial Implants. 2005;20(2):193-202. PubMed PMID: 15839112.

171. Uchida R, Nakata K, Kawano F, Yonetani Y, Ogasawara I, Nakai N, et al. Vibration acceleration promotes bone formation in rodent models. PLoS ONE 12 (3) (no pagination), 2017 Article Number: e0172614 Date of Publication: March 2017. PubMed PMID: 614670062.

172. Ugras A, Guzel E, Korkusuz P, Kaya I, Dikici F, Demirbas E, et al. Glucosamine-sulfate on fracture healing. Ulusal Travma ve Acil Cerrahi Dergisi 19 (1) (pp 8-12), 2013 Date of Publication: 2013. 2013. PubMed PMID: 2013171007.

173. Wada K, Yu W, Elazizi M, Barakat S, Ouimet MA, Rosario-Melendez R, et al. Locally delivered salicylic acid from a poly(anhydride-ester): impact on diabetic bone regeneration. Journal of Controlled Release. 2013;171(1):33-7. PubMed PMID: 23827476.

174. Wan L, Zhang F, He Q, Tsang WP, Lu L, Li Q, et al. EPO promotes bone repair through enhanced cartilaginous callus formation and angiogenesis.[Erratum appears in PLoS One. 2014;9(10):e111830]. PLoS ONE [Electronic Resource]. 2014;9(7):e102010. PubMed PMID: 25003898.

175. Wang G, Wang J, Fu Y, Bai L, He M, Li B, et al. Systemic treatment with vanadium absorbed by Coprinus comatus promotes femoral fracture healing in streptozotocin-diabetic rats. Biological Trace Element Research. 2013;151(3):424-33. PubMed PMID: 23271683.

176. Wang XL, Xie XH, Zhang G, Chen SH, Yao D, He K, et al. Exogenous phytoestrogenic molecule icaritin incorporated into a porous scaffold for enhancing bone defect repair. Journal of Orthopaedic Research. 2013;31(1):164-72. PubMed PMID: 22807243.

177. Wang Y, Lv P, Ma Z, Zhang J. Enhanced healing of rat calvarial critical size defect with selenium-doped lamellar biocomposites. Biological Trace Element Research 155 (1) (pp 72-81), 2013 Date of Publication: October 2013. 2013. PubMed PMID: 2013570659.

178. Wang AY, Tian Y, Yuan M, Zhang L, Chen JF, Xu WJ, et al. Effect of cervus and cucumis peptides on osteoblast activity and fracture healing in osteoporotic bone. Evidence-Based Complementary & Alternative Medicine: eCAM. 2014;2014:958908. PubMed PMID: 25525453.

179. Wang J, Tian XF, Wu SY, Meng XC, Wen GW. Accelerated healing by composites containing herb epimedium for osteoinductive regeneration. Biomedical Materials. 2014;9(3):035013. PubMed PMID: 24846988.

180. Werkman C, Senra GS, da Rocha RF, Brandao AA. Comparative therapeutic use of Risedronate and Calcarea phosphorica--allopathy versus homeopathy--in bone repair in castrated rats. Pesquisa Odontologica Brasileira = Brazilian Oral Research. 2006;20(3):196-201. PubMed PMID: 17119700.

181. Wixted JJ, Fanning PJ, Gaur T, O'Connell SL, Silva J, Mason-Savas A, et al. Enhanced fracture repair by leukotriene antagonism is characterized by increased chondrocyte proliferation and early bone formation: a novel role of the cysteinyl LT-1 receptor. Journal of Cellular Physiology. 2009;221(1):31-9. PubMed PMID: 19544365.

182. Wong RWK, Rabie ABM. Effect of naringin collagen graft on bone formation. Biomaterials 27 (9) (pp 1824-1831), 2006 Date of Publication: March 2006. 2006. PubMed PMID: 2005561760.

183. Wong RW, Rabie AB. Effect of quercetin on preosteoblasts and bone defects. The open orthopaedics journal. 2008;2:27-32. PubMed PMID: 19461927.

184. Wong RWK, Rabie ABM. Effect of Salvia miltiorrhiza extract on bone formation. Journal of Biomedical Materials Research - Part A 85 (2) (pp 506-512), 2008 Date of Publication: May 2008. 2008. PubMed PMID: 2008184935.

185. Wong E, Sangadala S, Boden SD, Yoshioka K, Hutton WC, Oliver C, et al. A novel low-molecular-weight compound enhances ectopic bone formation and fracture repair. Journal of Bone & Joint Surgery - American Volume. 2013;95(5):454-61. PubMed PMID: 23467869.

186. Woo KM, Jung HM, Oh JH, Rahman SU, Kim SM, Baek JH, et al. Synergistic effects of dimethyloxalylglycine and butyrate incorporated into alpha-calcium sulfate on bone regeneration. Biomaterials 39:1-14, 2015 Jan. PubMed PMID: 25477166.

187. Woodruff MA, Rath SN, Susanto E, Haupt LM, Hutmacher DW, Nurcombe V, et al. Sustained release and osteogenic potential of heparan sulfate-doped fibrin glue scaffolds within a rat cranial model. Journal of Molecular Histology. 2007;38(5):425-33. PubMed PMID: 17849224.

188. Xu Cz, Yang Wg, He Xf, Zhou Lt, Han Xk, Xu Xf. Vascular endothelial growth factor and nano-hydroxyapatite/collagen composite in the repair of femoral defect in rats. Journal of Clinical Rehabilitative Tissue Engineering Research 15 (38) (pp 7118-7122), 2011 Date of Publication: 2011. 2011. PubMed PMID: 2012467917.

189. Yoneme H, Hatakeyama J, Danjo A, Oida H, Yoshinari M, Aijima R, et al. Milk basic protein supplementation enhances fracture healing in mice. Nutrition 31 (2) (pp 399-405), 2015 Date of Publication: 01 Feb 2015. 2015. PubMed PMID: 2015669669.

190. Yoshii T, Nyman JS, Yuasa M, Esparza JM, Okawa A, Gutierrez GE. Local application of a proteasome inhibitor enhances fracture healing in rats. Journal of Orthopaedic Research. 2015;33(8):1197-204. PubMed PMID: 25683968.

191. Young S, Patel ZS, Kretlow JD, Murphy MB, Mountziaris PM, Baggett LS, et al. Dose effect of dual delivery of vascular endothelial growth factor and bone morphogenetic protein-2 on bone regeneration in a rat critical-size defect model. Tissue engineering. 2009;Part A.. 15(9):2347-62. PubMed PMID: 19249918.

192. Yu Z, Geng J, Gao H, Zhao X, Chen J. Evaluations of guided bone regeneration in canine radius segmental defects using autologous periosteum combined with fascia lata under stable external fixation. Journal of Orthopaedics and Traumatology 16 (2) (pp 133-140), 2015 Article Number: 321 Date of Publication: 12 Oct 2015. PubMed PMID: 600178332.

193. Zanchetta P, Lagarde N, Uguen A, Marcorelles P. Mixture of hyaluronic acid, chondroitin 6 sulphate and dermatan sulphate used to completely regenerate bone in rat critical size defect model. Journal of Cranio-Maxillo-Facial Surgery. 2012;40(8):783-7. PubMed PMID: 22464550.

194. Zhang Z, Shively JE. Acceleration of Bone Repair in NOD/SCID Mice by Human Monoosteophils, Novel LL-37-Activated Monocytes. PLoS ONE 8 (7) , 2013 Article Number: e67649 Date of Publication: 03 Jul 2013. 2013. PubMed PMID: 2013422276.

195. Zhang Y, Jing D, Buser D, Sculean A, Chandad F, Miron RJ. Bone grafting material in combination with Osteogain for bone repair: a rat histomorphometric study. Clinical Oral Investigations 20(3):589-95, 2016 Apr. PubMed PMID: 26174082.

196. Zhao DM, Yang JF, Wu SQ, Qiu LP, Liu JL, Wang HB, et al. [Effect of vascular endothelial growth factor 165 gene transfection on repair of bone defect: experiment with rabbits]. Chung-Hua i Hsueh Tsa Chih [Chinese Medical Journal]. 2007;87(25):1778-82. PubMed PMID: 17919388.

197. Zhao X, Wu ZX, Zhang Y, Gao MX, Yan YB, Cao PC, et al. Locally administrated perindopril improves healing in an ovariectomized rat tibial osteotomy model. PLoS ONE [Electronic Resource]. 2012;7(3):e33228. PubMed PMID: 22427998.
