# Supplementary material for: Preclinical therapies to prevent or treat fracture non-union: A systematic review
Source: PLoS One. 2018 Aug 1;13(8):e0201077. doi: 10.1371/journal.pone.0201077 (PMC6070249; doi:10.1371/journal.pone.0201077)
Supplement: S4 Table — (DOCX) [file pone.0201077.s004.docx]

**S4 Table:** Defect repair data for studies evaluating therapies based on animal derivatives (27 therapies, 18 studies)

| **Study** | **Therapy** | **Species** | **Maximum length of survival (days)** | **Outcome** | **Overall effect** |
| --- | --- | --- | --- | --- | --- |
| Almeida 2007[1] | Synthetic salmon calcitonin | Rats | 28 | No significant difference in bone density between control and therapeutic groups | = |
| Azavedo 2014[2] | Chitosan + beta-TCP | Rabbits | 90 | Significantly greater bone formation in therapeutic group compared to control group | ↑ |
| Azavedo 2014[2] | Chitosan | Rabbits | 90 | Significantly greater bone formation in therapeutic group compared to control group | ↑ |
| Bigham-Sadegh 2015[3] | Demineralised calf foetal growth plate (DCFGP) | Rabbits | 56 | There was no significant histopathological difference on statistical analysis | = |
| Bigham-Sadegh 2017[4] | Demineralised calf foetal growth plate (DCFGP) + coral | Rabbits | 56 | In the histopathological evaluation the defects of the animals in the coral-DCFGP, coral and DCFGP groups showed no significant differences in terms of statistical analysis | = |
| Bulbul 2008[5] | Salmon calcitonin | Rats | 70 | No statistical difference between therapeutic and control groups at 10 weeks | = |
| Canter 2010[6] | Chitosan | Rats | 98 | Less effect on defect repair when compared to autograft | ↓ |
| Canter 2010[6] | Chitosan + TGF-beta-2 | Rats | 98 | Less effect on defect repair when compared to autograft | ↓ |
| Canter 2010[6] | Chitosan + BMP-2 | Rats | 98 | No difference between autograft and chitosan/BMP group | = |
| Canter 2010[6] | Chitosan + TGF-beta-2 + BMP-2 | Rats | 98 | No difference between autograft and TGF/BMP group | = |
| Cui 2009[7] | Chitosan | Rabbits | 84 | Significantly greater new bone mineralisation in therapeutic group compared to other groups (data for blank control not presented) | ↑ |
| Ezoddini-Ardakani 2012[8] | Chitosan | Rats | 28 | Significantly greater bone healing seen in therapeutic group compared to control group at 4 weeks | ↑ |
| Fu 2015[9] | Lumbrokinase | Rats | 56 | Significantly greater bone formation in therapeutic group than in control group | ↑ |
| Hosaka 2013[10] | Squid derived chondroitin sulphate | Rats | 105 | Significantly greater bone repair in therapeutic group than control group | ↑ |
| Kanda 2015[11] | Porcine dermis | Rats | 56 | Increased bone formation with large pore sized acid extracted porcine dermis gel when compared to control | → |
| Katsumata 2015[12] | Salmon DNA | Mice | 84 | Salmon DNA implantation significantly induced bone deposition when compared to control | ↑ |
| Kim 2017b[13] | Silicon substituted cuttlefish bone hydroxyapatite (Si-CB-Hap) | Rabbits | 56 | The volume of new bone formed by the Si-CB-Hap group was significantly higher than that formed by the cuttlefish bone hydroxyapatite group | ↑ |
| Li 2016[14] | Erythropoietin on deproteinised bovine bone (DBB) scaffold | Goats | 84 | Significantly higher grey values in therapeutic group compared to DBB scaffold alone group at 12 weeks, but no difference between therapeutic group and autogenous cancellous bone graft | → |
| Meimandi Parizi 2015[15] | Glycolipoprotein extract (G90) from Eisenia foetida | Rabbits | 60 | Therapeutic group demonstrated superior osteogenic potential in the healing of the defect, though there were no significant differences | → |
| Park 2008[16] | Dentin + chitosan | Rats | 56 | Significantly greater bone formation in therapeutic group compared to control group at 8 weeks | ↑ |
| Park 2008[16] | Chitosan | Rats | 56 | Significantly greater bone formation in chitosan group compared to control group and compared to dentin group at 8 weeks | ↑ |
| Tolli 2011[17] | 2mg reindeer bone extract | Rats | 42 | No significant difference between therapeutic and untreated or collagen alone groups | = |
| Tolli 2011[17] | 5mg reindeer bone extract | Rats | 42 | No significant difference between therapeutic and untreated or collagen alone groups | = |
| Tolli 2011[17] | 15mg reindeer bone extract | Rats | 42 | Significantly greater bone healing in defect area compared to untreated and collagen alone group | ↑ |
| Tolli 2011[17] | 20mg reindeer bone extract | Rats | 42 | Significantly greater bone healing in defect area in therapeutic group compared to untreated group, and compared to collagen alone group | ↑ |
| Tollli 2011[17] | 50mg reindeer bone extract | Rats | 42 | Significantly greater bone healing in defect area compared to untreated and collagen alone group | ↑ |
| Yoneme 2015[18] | Milk basic protein | Mice | 28 | No significant difference between therapeutic and control groups | = |

↑ indicates statistically significant effect on bone formation in trial therapy compared to control

→ indicates greater bone formation in trial therapy compared to control, but the effect did not reach statistical significance

= indicates no difference in bone formation rates between the therapeutic or control groups

↓ indicates less effect on bone formation in trial therapy compared to control

1. Almeida JD, Arisawa EA, da Rocha RF, Carvalho YR. Effect of calcitonin on bone regeneration in male rats: a histomorphometric analysis. International Journal of Oral & Maxillofacial Surgery. 2007;36(5):435-40. PubMed PMID: 17275259.

2. Azevedo AS, Sa MJ, Fook MV, Neto PI, Sousa OB, Azevedo SS, et al. Use of chitosan and beta-tricalcium phosphate, alone and in combination, for bone healing in rabbits. Journal of Materials Science-Materials in Medicine. 2014;25(2):481-6. PubMed PMID: 24243224.

3. Bigham-Sadegh A, Karimi I, Shadkhast M, Mahdavi MH. Hydroxyapatite and demineralized calf fetal growth plate effects on bone healing in rabbit model. Journal of Orthopaedics & Traumatology 16(2):141-9, 2015 Jun. PubMed PMID: 25308902.

4. Bigham-Sadegh A, Mohamadnia AR, Shahbazkia HR, Khalilifard S. Role of Coral, demineralized calf fetal growth plate, and a combination of the two in healing of bone defects in Rabbits. Trauma Monthly 22 (2) (no pagination), 2017 Article Number: e28275 Date of Publication: March 2017. 2017. PubMed PMID: 615138815.

5. Bulbul M, Esenyel CZ, Esenyel M, Ayanoglu S, Bilgic B, Gulmez T. Effects of calcitonin on the biomechanics, histopathology, and radiography of callus formation in rats. Journal of Orthopaedic Science 13 (2) (pp 136-144), 2008 Date of Publication: March 2008. 2008. PubMed PMID: 2008172919.

6. Canter HI, Vargel I, Korkusuz P, Oner F, Gungorduk DB, Cil B, et al. Effect of use of slow release of bone morphogenetic protein-2 and transforming growth factor-Beta-2 in a chitosan gel matrix on cranial bone graft survival in experimental cranial critical size defect model. Annals of Plastic Surgery. 2010;64(3):342-50. PubMed PMID: 20179488.

7. Cui X, Zhao D, Zhang B, Gao Y. Osteogenesis mechanism of chitosan-coated calcium sulfate pellets on the restoration of segmental bone defects. Journal of Craniofacial Surgery. 2009;20(5):1445-50. PubMed PMID: 19816276.

8. Ezoddini-Ardakani F, Navabazam A, Fatehi F, Danesh-Ardekani M, Khadem S, Rouhi G. Histologic evaluation of chitosan as an accelerator of bone regeneration in microdrilled rat tibias. Dental Research Journal. 2012;9(6):694-9. PubMed PMID: 23559943.

9. Fu YT, Sheu SY, Chen YS, Chen KY, Yao CH. Porous gelatin/tricalcium phosphate/genipin composites containing lumbrokinase for bone repair. Bone. 2015;78 (pp 15-22), 2015. Date of Publication:September 01. PubMed PMID: 2015028038.

10. Hosaka YZ, Iwai Y, Tamura J, Uehara M. Diamond squid (Thysanoteuthis rhombus)-derived chondroitin sulfate stimulates bone healing within a rat calvarial defect. Marine Drugs. 2013;11(12):5024-35. PubMed PMID: 24335526.

11. Kanda N, Anada T, Handa T, Kobayashi K, Ezoe Y, Takahashi T, et al. Orthotopic Osteogenecity Enhanced by a Porous Gelatin Sponge in a Critical-Sized Rat Calvaria Defect. Macromolecular Bioscience 15 (12) (pp 1647-1655), 2015 Date of Publication: 01 Dec 2015. PubMed PMID: 605297923.

12. Katsumata Y, Kajiya H, Okabe K, Fukushima T, Ikebe T. A salmon DNA scaffold promotes osteogenesis through activation of sodium-dependent phosphate cotransporters. Biochemical & Biophysical Research Communications 468(4):622-8, 2015 Dec 25. PubMed PMID: 26551467.

13. Kim BS, Yang SS, Yoon JH, Lee J. Enhanced bone regeneration by silicon-substituted hydroxyapatite derived from cuttlefish bone. Clinical Oral Implants Research 28(1):49-56, 2017 Jan. PubMed PMID: 26073102.

14. Li D, Deng L, Xie X, Yang Z, Kang P. Evaluation of the osteogenesis and angiogenesis effects of erythropoietin and the efficacy of deproteinized bovine bone/recombinant human erythropoietin scaffold on bone defect repair. Journal of Materials Science-Materials in Medicine 27(6):101, 2016 Jun. PubMed PMID: 27091043.

15. Meimandi Parizi A, Oryan A, Haddadi S, Bigham Sadegh A. Histopathological and biomechanical evaluation of bone healing properties of DBM and DBM-G90 in a rabbit model. Acta orthopaedica et traumatologica turcica 49 (6) (pp 683-689), 2015 Date of Publication: 2015. PubMed PMID: 611671838.

16. Park SS, Kim SG, Lim SC, Ong JL. Osteogenic activity of the mixture of chitosan and particulate dentin. Journal of Biomedical Materials Research. 2008;Part A. 87(3):618-23. PubMed PMID: 18186071.

17. Tolli H, Kujala S, Jamsa T, Jalovaara P. Reindeer bone extract can heal the critical-size rat femur defect. International Orthopaedics. 2011;35(4):615-22. PubMed PMID: 20454894.

18. Yoneme H, Hatakeyama J, Danjo A, Oida H, Yoshinari M, Aijima R, et al. Milk basic protein supplementation enhances fracture healing in mice. Nutrition 31 (2) (pp 399-405), 2015 Date of Publication: 01 Feb 2015. 2015. PubMed PMID: 2015669669.
