# Supplementary material for: Preclinical therapies to prevent or treat fracture non-union: A systematic review
Source: PLoS One. 2018 Aug 1;13(8):e0201077. doi: 10.1371/journal.pone.0201077 (PMC6070249; doi:10.1371/journal.pone.0201077)
Supplement: S5 Table — (DOCX) [file pone.0201077.s005.docx]

**S5 Table:** Defect repair data for studies evaluating therapies based on plant extracts (23 therapies, 23 studies)

| **Study** | **Therapy** | **Species** | **Maximum length of survival (days)** | **Outcome** | **Overall effect** |
| --- | --- | --- | --- | --- | --- |
| Burim 2016[1] | Icariin | Rats | 42 | Significantly greater area of new bone formation in therapeutic group compared to control group | ↑ |
| Casarin 2014[2] | Resveratrol | Rats | 30 | Significant reduction in remaining defect in therapeutic group compared to control group | ↑ |
| Chen 2013a[3] | Naringin | Rabbits | 56 | A greater amount of bone ingrowth in the therapeutic group than in the control group | → |
| Chen 2013b[4] | Icaritin | Rabbits | 56 | Significantly greater bone volume in the therapeutic group at all concentrations, with the highest being seen in the medium concentration group compared to control | ↑ |
| De Abreu 2016[5] | Sugarcane biopolymer | Rats | 120 | Bone repair seen in control group only, with none seen in therapeutic group | ↓ |
| De Mendonca 2006[6] | Castor oil polyurethane | Rabbits | 90 | Bone repair seen in the control group only, which was complete at 90 days | ↓ |
| Dosier 2015[7] | Alginate hydrogel to deliver BMPs | Rats | 84 | Significantly more bone observed in BMP2 positive hydrogels than in control group | ↑ |
| Ereno 2010[8] | Natural latex | Rabbits | 120 | No significant difference in radiological density between therapeutic and control groups | = |
| Giaversi 2010[9] | Soybean | Rabbits | 168 | No significant difference in bone healing rate between therapeutic and control groups at week 24 | = |
| Gunay 2013[10] | Ankaferd blood stopper | Rats | 45 | No significant difference between therapeutic and control groups | = |
| Huh 2009[11] | Formononetin | Rats | 21 | Formononetin plays important role in bone formation through angiogenic and osteogenic effects | ? |
| Issa 2012[12] | P-1 (latex protein) | Rats | 28 | Statistically significant increase in bone formation in group treated with 10μg of P-1 in combination with monoolein gel with or without BMP2, but no increase in bone formation with 5μg or 10μg of pure P-1 | ↑ |
| Khedgikar 2017[13] | Flavonoids from *Dalbergia Sissoo* | Rats | 14 | Administration of ethanol extract of *Dalbergia Sissoo* induced a significantly higher mineral deposition over the control | ↑ |
| Li 2017[14] | Icariin | Mice | 56 | Defects implanted with icariin exhibited higher new bone formation than control defects at 8 weeks | → |
| Medeiros 2013[15] | Sugarcane biopolymer hydrogel | Rats | 180 | Significantly greater bone tissue formation in therapeutic group compared to control | ↑ |
| Merolli 2010[16] | Soybean filler | Rabbits | 56 | Significantly greater bone in-growth in therapeutic group compared to control group | ↑ |
| Silva 2015[17] | Copaiba oil | Rats | 40 | No significant difference in bone matrix mineralisation between groups | = |
| Wang 2013b[18] | Icaritin | Rabbits | 84 | Higher bone formation in therapeutic group compared to control group | → |
| Wang 2014a[19] | Herb epimedium | Rabbits | 84 | No significant difference in mineralised bone area between control and therapeutic groups | = |
| Wang 2014b[20] | Cervus and cucumis peptides | Rats | 84 | Significantly higher bone formation in herb epimedium group versus comparator | ↑ |
| Wong 2006[21] | Naringin | Rabbits | 14 | Significantly greater area of newly formed bone in therapeutic group compared to control group | ↑ |
| Wong 2008a[22] | Salvia miltiorrhiza | Rabbits | 14 | Significantly greater area of newly formed bone in therapeutic group compared to control group | ↑ |
| Wong 2008b[23] | Quercetin | Rabbits | 14 | No difference between therapeutic and control groups | = |

↑ indicates statistically significant effect on bone formation in trial therapy compared to control

→ indicates greater bone formation in trial therapy compared to control, but the effect did not reach statistical significance

= indicates no difference in bone formation rates between the therapeutic or control groups

↓ indicates less effect on bone formation in trial therapy compared to control

? indicates results are unclear, and no effect size could be determined

1. Burim RA, Sendyk DI, Hernandes LS, de Souza DF, Correa L, Deboni MC. Repair of Critical Calvarias Defects With Systemic Epimedium sagittatum Extract. Journal of Craniofacial Surgery 27(3):799-804, 2016 May. PubMed PMID: 26982112.

2. Casarin RC, Casati MZ, Pimentel SP, Cirano FR, Algayer M, Pires PR, et al. Resveratrol improves bone repair by modulation of bone morphogenetic proteins and osteopontin gene expression in rats. International Journal of Oral & Maxillofacial Surgery. 2014;43(7):900-6. PubMed PMID: 24530035.

3. Chen KY, Lin KC, Chen YS, Yao CH. A novel porous gelatin composite containing naringin for bone repair. Evidence-Based Complementary & Alternative Medicine: eCAM. 2013;2013:283941. PubMed PMID: 23431335.

4. Chen SH, Lei M, Xie XH, Zheng LZ, Yao D, Wang XL, et al. PLGA/TCP composite scaffold incorporating bioactive phytomolecule icaritin for enhancement of bone defect repair in rabbits. Acta Biomaterialia. 2013;9(5):6711-22. PubMed PMID: 23376238.

5. De Abreu TC, De Lima RP, De Souza VSB, Junior OC, De Albuquerque AV, Aguiar JLA, et al. The biopolymer sugarcane as filling material of critical defects in rats. Acta Cirurgica Brasileira 31 (1) (pp 53-58), 2016 Date of Publication: January 2016. PubMed PMID: 608139565.

6. De Mendonca JCG, De Rossi R, Inouye CM, Bazan DRP, Monteiro JCC, De Mendonca JP. Morphology of autogenous bone graft and castor oil polyurethane in the infraorbital rim of rabbits: A comparative study. Acta Cirurgica Brasileira 21 (5) (pp 341-347), 2006 Date of Publication: September/October 2006. 2006. PubMed PMID: 2006499857.

7. Dosier CR, Uhrig BA, Willett NJ, Krishnan L, Li MT, Stevens HY, et al. Effect of cell origin and timing of delivery for stem cell-based bone tissue engineering using biologically functionalized hydrogels. Tissue engineering. 2015;Part A.. 21(1-2):156-65. PubMed PMID: 25010532.

8. Ereno C, Guimaraes SAC, Pasetto S, Herculano RD, Silva CP, Graeff CFO, et al. Latex use as an occlusive membrane for guided bone regeneration. Journal of Biomedical Materials Research - Part A 95 (3 A) (pp 932-939), 2010 Date of Publication: 01 Dec 2010. 2010. PubMed PMID: 2010622042.

9. Giavaresi G, Fini M, Salvage J, Nicoli Aldini N, Giardino R, Ambrosio L, et al. Bone regeneration potential of a soybean-based filler: experimental study in a rabbit cancellous bone defects. Journal of Materials Science-Materials in Medicine. 2010;21(2):615-26. PubMed PMID: 19771493.

10. Gunay M, Amanvermez R, Keles G. Ankaferd Blood Stopper: Does it have a role in fracture healing? Turkish Journal of Medical Sciences 43 (5) (pp 733-738), 2013 Date of Publication: 2013. 2013. PubMed PMID: 2013536190.

11. Huh JE, Kwon NH, Baek YH, Lee JD, Choi DY, Jingushi S, et al. Formononetin promotes early fracture healing through stimulating angiogenesis by up-regulating VEGFR-2/Flk-1 in a rat fracture model. International Immunopharmacology. 2009;9(12):1357-65. PubMed PMID: 19695348.

12. Issa JP, Defino HL, Pereira YC, Netto JC, Sebald W, Bentley MV, et al. Bone repair investigation using rhBMP-2 and angiogenic protein extracted from latex. Microscopy Research & Technique. 2012;75(2):145-52. PubMed PMID: 21761496.

13. Khedgikar V, Kushwaha P, Ahmad N, Gautam J, Kumar P, Maurya R, et al. Ethanolic extract of Dalbergia sissoo promotes rapid regeneration of cortical bone in drill-hole defect model of rat. Biomedicine & Pharmacotherapy 86:16-22, 2017 Feb. PubMed PMID: 27936389.

14. Li M, Gu Q, Chen M, Zhang C, Chen S, Zhao J. Controlled delivery of icariin on small intestine submucosa for bone tissue engineering. Materials Science & Engineering C, Materials for Biological Applications 71:260-267, 2017 Feb 01. PubMed PMID: 27987707.

15. Medeiros Junior MD, Carvalho EJ, Catunda IS, Bernardino-Araujo S, Aguiar JL. Hydrogel of polysaccharide of sugarcane molasses as carrier of bone morphogenetic protein in the reconstruction of critical bone defects in rats. Acta Cirurgica Brasileira. 2013;28(4):233-8. PubMed PMID: 23568229.

16. Merolli A, Nicolais L, Ambrosio L, Santin M. A degradable soybean-based biomaterial used effectively as a bone filler in vivo in a rabbit. Biomedical Materials. 2010;5(1):15008. PubMed PMID: 20124667.

17. Silva PF, Brito MV, Pontes FS, Ramos SR, Mendes LC, Oliveira LC. Copaiba oil effect on experimental jaw defect in Wistar rats. Acta Cirurgica Brasileira. 2015;30(2):120-6. PubMed PMID: 25714691.

18. Wang XL, Xie XH, Zhang G, Chen SH, Yao D, He K, et al. Exogenous phytoestrogenic molecule icaritin incorporated into a porous scaffold for enhancing bone defect repair. Journal of Orthopaedic Research. 2013;31(1):164-72. PubMed PMID: 22807243.

19. Wang J, Tian XF, Wu SY, Meng XC, Wen GW. Accelerated healing by composites containing herb epimedium for osteoinductive regeneration. Biomedical Materials. 2014;9(3):035013. PubMed PMID: 24846988.

20. Wang AY, Tian Y, Yuan M, Zhang L, Chen JF, Xu WJ, et al. Effect of cervus and cucumis peptides on osteoblast activity and fracture healing in osteoporotic bone. Evidence-Based Complementary & Alternative Medicine: eCAM. 2014;2014:958908. PubMed PMID: 25525453.

21. Wong RWK, Rabie ABM. Effect of naringin collagen graft on bone formation. Biomaterials 27 (9) (pp 1824-1831), 2006 Date of Publication: March 2006. 2006. PubMed PMID: 2005561760.

22. Wong RWK, Rabie ABM. Effect of Salvia miltiorrhiza extract on bone formation. Journal of Biomedical Materials Research - Part A 85 (2) (pp 506-512), 2008 Date of Publication: May 2008. 2008. PubMed PMID: 2008184935.

23. Wong RW, Rabie AB. Effect of quercetin on preosteoblasts and bone defects. The open orthopaedics journal. 2008;2:27-32. PubMed PMID: 19461927.
