# Supplementary material for: Preclinical therapies to prevent or treat fracture non-union: A systematic review
Source: PLoS One. 2018 Aug 1;13(8):e0201077. doi: 10.1371/journal.pone.0201077 (PMC6070249; doi:10.1371/journal.pone.0201077)
Supplement: S6 Table — (DOCX) [file pone.0201077.s006.docx]

**S6 Table:** Defect repair data for studies evaluating therapies based on minerals, elements or chemicals (25 therapies, 21 studies)

| **Study** | **Therapy** | **Species** | **Maximum length of survival (days)** | **Outcome** | **Overall effect** |
| --- | --- | --- | --- | --- | --- |
| Azavedo 2014[1] | Chitosan + beta-TCP | Rabbits | 90 | Significantly greater bone formation in therapeutic group compared to control group | ↑ |
| Azavedo 2014[1] | Beta-TCP | Rabbits | 90 | Significantly greater bone formation in therapeutic group compared to control group | ↑ |
| Bae 2016[2] | Sodium triphosphate | Mice | 42 | Significantly greater bone formation than sodium hexametaphosphate group on micro-CT | ↑ |
| Bae 2016[2] | Sodium hexametaphosphate | Mice | 42 | Scant bone regeneration on micro CT | = |
| Cai 2008[3] | OIC-A006 | Rabbits | 28 | Significant increase in bone formation in therapeutic group | ↑ |
| Chen 2015[4] | Boron | Rats | 56 | Bone islands were observed in the centre of defects with abundant bone formation peripherally; boron containing scaffolds had significantly higher new bone tissue | ? |
| Cheng 2016[5] | Strontium + silver | Rats | 42 | Strontium and silver nanotubule groups showed enhanced healing and a higher extent of new bone formation, with almost complete healing 6 weeks after surgery | → |
| Donigan 2012[6] | Nicotine | Rabbits | 21 | No difference between control and therapeutic groups | = |
| Ge 2011[7] | Diplen-Gam | Rats | 84 | Significantly more bone formation in the control (BioGlide) group compared to the Diplen-Gam group at week 12 | ↓ |
| He 2008[8] | Calcium alginate | Rabbits | 42 | Significantly greater bone area regeneration in therapeutic group compared to control group | ↑ |
| Hokugo 2016[9] | 1mg oxysterol | Rabbits | 42 | Increase in percentage of bone volume/total volume ratio but not significant | → |
| Hokugo 2016[9] | 10mg oxysterol | Rabbits | 42 | Significant increase in bone volume/total volume ratio percentage compared to control | ↑ |
| Hreha 2015[10] | Manganese chloride | Rats | 14 | No significant difference between control and therapeutic groups in female rats; greater percentage of mineralised tissue in male rats treated with therapy than in control groups | =/→ |
| Hu 2016[11] | Silk fibroin | Rats | 21 | Histological examination demonstrated rats treated with silk fibroin composite exhibited extensive osteogenesis in and nearby the defect site | ? |
| Lu 2005[12] | Octyl-a-cyanoacrylate | Rabbits | 84 | At the 12th week the woven bone density got close to that of bone tissues and bridged the broken ends. Bone union was nearly complete. Bone intensity of the control group became close to that of normal bone tissues | ? |
| Morse 2016[13] | RAP-011 | Rats | 42 | Significantly larger callus with an increase in bone volume in therapeutic group compared to control group | ↑ |
| Nacer 2015[14] | Silicon | Rats | 60 | After 60 days increase of bone growth with abundant presence of mature osseous tissue | ? |
| Nacer 2015[14] | Barium | Rats | 60 | Bone defect partially occupied by lamellar bone; great proximity of mature osseous tissue with the biomaterial was noticed | ? |
| Ortolan 2014[15] | Chalcone | Rats | 45 | Significantly less wound area remaining in therapeutic group compared to control group | ↑ |
| Oryan 2014[16] | Osteogenic medium | Rabbits | 56 | Significantly greater bone formation in therapeutic group compared to control group | ↑ |
| Park 2008[17] | Dentin + plaster of Paris | Rats | 56 | Significantly greater bone formation in therapeutic group compared to control group | ↑ |
| Saghiri 2015[18] | White mineral trioxide aggregate | Rabbits | 60 | Statistically significant increase in bone regeneration in therapeutic groups | ↑ |
| Werkman 2006[19] | Calcarea phosphorica | Rats | 28 | No significant difference between therapeutic and control groups | = |
| Wong 2013[20] | SVAK-12 | Rats | 35 | Significantly higher radiographic healing scores in therapeutic group compared to control | ↑ |
| Woo 2015[21] | Butyrate | Rats | 56 | Significant increase in volume of radio-opaque matter in experimental group | ↑ |

↑ indicates statistically significant effect on bone formation in trial therapy compared to control

→ indicates greater bone formation in trial therapy compared to control, but the effect did not reach statistical significance

= indicates no difference in bone formation rates between the therapeutic or control groups

↓ indicates less effect on bone formation in trial therapy compared to control

? indicates results are unclear, and no effect size could be determined

1. Azevedo AS, Sa MJ, Fook MV, Neto PI, Sousa OB, Azevedo SS, et al. Use of chitosan and beta-tricalcium phosphate, alone and in combination, for bone healing in rabbits. Journal of Materials Science-Materials in Medicine. 2014;25(2):481-6. PubMed PMID: 24243224.

2. Bae WJ, Auh QS, Kim GT, Moon JH, Kim EC. Effects of sodium tri- and hexameta-phosphate in vitro osteoblastic differentiation in Periodontal Ligament and Osteoblasts, and in vivo bone regeneration. Differentiation. 2016;92(5):257-69. doi: 10.1016/j.diff.2016.04.004. PubMed PMID: 27160630.

3. Cai M, Liu X, Shao J, Qi J, Wang J, Zhu Y, et al. OIC-A006 promotes osteogenesis in vitro and in vivo. Pharmazie. 2008;63(10):751-6. PubMed PMID: 18972839.

4. Chen X, Zhao Y, Geng S, Miron RJ, Zhang Q, Wu C, et al. In vivo experimental study on bone regeneration in critical bone defects using PIB nanogels/boron-containing mesoporous bioactive glass composite scaffold. International Journal of Nanomedicine 10:839-46, 2015. PubMed PMID: 25653525.

5. Cheng H, Xiong W, Fang Z, Guan H, Wu W, Li Y, et al. Strontium (Sr) and silver (Ag) loaded nanotubular structures with combined osteoinductive and antimicrobial activities. Acta Biomaterialia 31:388-400, 2016 Feb. PubMed PMID: 26612413.

6. Donigan JA, Fredericks DC, Nepola JV, Smucker JD. The effect of transdermal nicotine on fracture healing in a rabbit model. Journal of Orthopaedic Trauma. 2012;26(12):724-7. PubMed PMID: 22955337.

7. Ge Y, Feng H, Wang L. Application of a novel resorbable membrane in the treatment of calvarial defects in rats. Journal of Biomaterials Science, Polymer Edition 22 (18) (pp 2417-2429), 2011 Date of Publication: 2011. 2011. PubMed PMID: 2011616926.

8. He H, Yan W, Chen G, Lu Z. Acceleration of de novo bone formation with a novel bioabsorbable film: a histomorphometric study in vivo. Journal of Oral Pathology & Medicine. 2008;37(6):378-82. PubMed PMID: 18355176.

9. Hokugo A, Sorice S, Parhami F, Yalom A, Li A, Zuk P, et al. A novel oxysterol promotes bone regeneration in rabbit cranial bone defects. Journal Of Tissue Engineering & Regenerative Medicine 10(7):591-9, 2016 Jul. PubMed PMID: 23997014.

10. Hreha J, Wey A, Cunningham C, Krell ES, Brietbart EA, Paglia DN, et al. Local manganese chloride treatment accelerates fracture healing in a rat model. Journal of Orthopaedic Research 33(1):122-30, 2015 Jan. PubMed PMID: 25231276.

11. Hu JX, Ran JB, Chen S, Jiang P, Shen XY, Tong H. Carboxylated Agarose (CA)-Silk Fibroin (SF) Dual Confluent Matrices Containing Oriented Hydroxyapatite (HA) Crystals: Biomimetic Organic/Inorganic Composites for Tibia Repair. Biomacromolecules 17 (7) (pp 2437-2447), 2016 Date of Publication: 11 Jul 2016. PubMed PMID: 611180645.

12. Lu B, Tu ZQ, Pei FX, Liu L. Octyl-a-cyanoacrylate adhesive in the treatment of tibial transverse fracture in rabbits. Chinese Journal of Traumatology. 2005;8(4):240-4. PubMed PMID: 16042872.

13. Morse A, Cheng TL, Peacock L, Mikulec K, Little DG, Schindeler A. RAP‐011 augments callus formation in closed fractures in rats. Journal of Orthopaedic Research. 2016;34(2):320-30.

14. Nacer RS, Silva BA, Poppi RR, Silva DK, Cardoso VS, Delben JR, et al. Biocompatibility and osteogenesis of the castor bean polymer doped with silica (SiO2) or barium titanate (BaTiO3) nanoparticles. Acta Cirurgica Brasileira 30(4):255-63, 2015 Apr. PubMed PMID: 25923258.

15. Ortolan XR, Fenner BP, Mezadri TJ, Tames DR, Correa R, de Campos Buzzi F. Osteogenic potential of a chalcone in a critical-size defect in rat calvaria bone. Journal of Cranio-Maxillo-Facial Surgery. 2014;42(5):520-4. PubMed PMID: 24041609.

16. Oryan A, Bigham-Sadegh A, Abbasi-Teshnizi F. Effects of osteogenic medium on healing of the experimental critical bone defect in a rabbit model. Bone. 2014;63:53-60. PubMed PMID: 24582803.

17. Park SS, Kim SG, Lim SC, Ong JL. Osteogenic activity of the mixture of chitosan and particulate dentin. Journal of Biomedical Materials Research. 2008;Part A. 87(3):618-23. PubMed PMID: 18186071.

18. Saghiri MA, Orangi J, Tanideh N, Asatourian A, Janghorban K, Garcia-Godoy F, et al. Repair of bone defect by nano-modified white mineral trioxide aggregates in rabbit: A histopathological study. Medicina Oral, Patologia Oral y Cirugia Bucal 20(5):e525-31, 2015 Sep 01. PubMed PMID: 26034924.

19. Werkman C, Senra GS, da Rocha RF, Brandao AA. Comparative therapeutic use of Risedronate and Calcarea phosphorica--allopathy versus homeopathy--in bone repair in castrated rats. Pesquisa Odontologica Brasileira = Brazilian Oral Research. 2006;20(3):196-201. PubMed PMID: 17119700.

20. Wong E, Sangadala S, Boden SD, Yoshioka K, Hutton WC, Oliver C, et al. A novel low-molecular-weight compound enhances ectopic bone formation and fracture repair. Journal of Bone & Joint Surgery - American Volume. 2013;95(5):454-61. PubMed PMID: 23467869.

21. Woo KM, Jung HM, Oh JH, Rahman SU, Kim SM, Baek JH, et al. Synergistic effects of dimethyloxalylglycine and butyrate incorporated into alpha-calcium sulfate on bone regeneration. Biomaterials 39:1-14, 2015 Jan. PubMed PMID: 25477166.
