# Supplementary material for: Preclinical therapies to prevent or treat fracture non-union: A systematic review
Source: PLoS One. 2018 Aug 1;13(8):e0201077. doi: 10.1371/journal.pone.0201077 (PMC6070249; doi:10.1371/journal.pone.0201077)
Supplement: S7 Table — (DOCX) [file pone.0201077.s007.docx]

**S7 Table:** Defect repair data for studies evaluating therapies based on cells and tissues (32 therapies, 27 studies)

| **Study** | **Therapy** | **Species** | **Maximum length of survival (days)** | **Outcome** | **Overall effect** |
| --- | --- | --- | --- | --- | --- |
| Ayanoglu 2015[1] | Hyaluronic acid | Rabbits | 42 | No difference in histological healing | = |
| Bahney 2016[2] | Cartilage (human osteoarthritic) | Mice | 28 | Integration between grafts and host bone occurred at 15/16 junctions: the amount of bony repair tissue was an average of 48%. In half of the animals the woven bone bridged the defects; in contrast, none of four control defects exhibited bridging with bone or cartilage | ? |
| Bardlsey 2017[3] | Cartilage | Rats | 84 | By week 12, micro-CT data showed that 38.48% of the defect was mineralised compared to the untreated defect (7.56%) and further remodelling of the hypertrophic cartilage into bone was also observed histologically. Little bone formation was observed with the calvarial defects that had remained empty | ? |
| Cakmak 2015[4] | Pentoxyfylline + iliac crest autograft | Rats | 56 | Radiological bone union was observed in the iliac crest autograft and systemic pentoxyfylline group compared to no new bone growth in the control group | ? |
| Cheung 2010[5] | Adipose stromal cells | Rats | 84 | Increased bone volume fraction within defects treated in the therapeutic group | → |
| Fan 2016[6] | Bone marrow-derived mesenchymal stem cells (BMSCs) | Rats | 56 | Increased bone formation in SHP-2 gene transduced bone marrow-derived mesenchymal stem cells group compared to standard BMSCs | → |
| Fukui 2015[7] | Human granulocyte colony stimulating factor mobilised CD34 positive cells | Rats | 84 | Frequency of bone union was significantly greater in the CD34 positive group than the control at week 12 | ↑ |
| Fukui 2015[7] | Human granulocyte colony stimulating factor mobilised mononuclear cells | Rats | 84 | Non-union healing was observed in 30% of rats at week 12 in the MNC group, compared to no bridging callus formation during 12 weeks of observation in the control group | ? |
| Furuta 2016[8] | Mesenchymal stem cell exosomes | Mice | 10 | The timing of bone union was significantly shorter in mice treated with conditioned medium and exosomes compared with control | ↑ |
| Grassmann 2015[9] | Bone marrow concentrate + hyperbaric oxygen therapy | Rabbits | 42 | Significantly higher bone defect consolidation in hyperbaric oxygen + bone marrow concentrate group compared to bone marrow concentrate group alone | ↑ |
| Hao 2016[10] | Bone mesenchymal stem cells seeded fibrin glue | Rats | 56 | No statistical difference between control and intervention group based on subjective histological outcome measures | = |
| He 2017[11] | Low frequency vibration + bone marrow stromal cells (BMSCs) | Rabbits | 28 | Low frequency vibrations ideally at 50 Hz promote the differentiation of BMSCs into osteoblasts | ? |
| Heo 2015[12] | Periostin + human adipose stromal cells | Mice | 56 | Significant reduction in bone gap compared to control | ↑ |
| Heo 2015[12] | Human adipose stromal cells | Mice | 56 | No significant reduction in gap of defect compared to control | = |
| Jensen 2016[13] | Bone marrow stromal cells (BMSCs) | Pigs | 35 | Increased regenerative osteogenic potential of dental pulp stromal cells (DPSCs) compared to BMSCs | ? |
| Jensen 2016[13] | Dental pulp stromal cells (DPSCs) | Pigs | 35 | Increased regenerative osteogenic potential of DPSCs compared to BMSCs | ? |
| Kim 2015a[14] | Fibrin | Rabbits | 56 | Fibrin administration resulted in greater percentage of bone volume formation when compared to control at 8 weeks | → |
| Kim 2015b[15] | Fibrinogen | Rabbits | 56 | Addition of fibrinogen to biphasic calcium phosphate scaffolds increased bone formation at 8 weeks, both above biphasic calcium phosphate alone, and empty controls | → |
| Kim 2017a[16] | Atelocollagen | Rabbits | 56 | The percentage of new bone growth in the Atelocollagen group was significantly higher than in the control group | ↑ |
| Lattanzi 2008[17] | Skin fibroblasts upregulating LMP-3 | Rats | 84 | Improved bone formation in therapeutic group compared to control | → |
| Levy 2016[18] | Immature myeloid cells | Mice | 21 | Transfer of activated immature myeloid cells significantly improved fracture healing by inducing more bone growth by 6 weeks post fracture, although this was not sufficient to heal the fracture completely | ↑ |
| Lin 2007[19] | Adipose stromal cells | Rats | 168 | Significantly greater bone formation in therapeutic group compared to control group | ↑ |
| Park 2008[20] | Dentin + chitosan | Rats | 56 | Significantly greater bone formation in therapeutic group compared to control group | ↑ |
| Park 2008[20] | Dentin + plaster of Paris | Rats | 56 | Significantly greater bone formation in therapeutic group compared to control group | ↑ |
| Park 2008[20] | Dentin | Rats | 56 | Significantly greater bone formation in therapeutic group compared to control group | ↑ |
| Peled 2007[21] | Hydrogel plug of fibrinogen and polyethylene glycol | Rats | 35 | Large amount of new bone formation in therapeutic group compared to control group | → |
| Santo 2015[22] | Dexamethasone micelles for bone marrow-derived mesenchymal stem cell regulation | Rats | 28 | Significant increase in new bone volume in treatment group compared to control | ↑ |
| Serrano 2013[23] | Cementum protein 1 | Rats | 112 | Greater filling of defect with mineralised tissue in therapeutic group compared to control group | → |
| Song 2011[24] | Co-transduction of bone marrow-derived mesenchymal stem cells by basic fibroblast growth factor | Rats | 28 | Significantly higher bone area density in genetically modified therapeutic group compared to control | ↑ |
| Zanchetta 2012[25] | Hyaluronic acid + chondroitin sulphate | Rats | 21 | All treatment groups showed almost complete bone healing, while the control hole also healed completely | ? |
| Zhang 2013[26] | Human monoosteophils | Mice | 24 | Greater rate of union in therapeutic group compared to control group | → |
| Zhang 2016[27] | Osteogain (enamel matrix derivative) | Rats | 56 | Significantly greater new bone formation in experimental group | ↑ |

↑ indicates statistically significant effect on bone formation in trial therapy compared to control

→ indicates greater bone formation in trial therapy compared to control, but the effect did not reach statistical significance

= indicates no difference in bone formation rates between the therapeutic or control groups

? indicates results are unclear, and no effect size could be determined

1. Ayanoglu S, Esenyel CZ, Adanir O, Dedeoglu S, Imren Y, Esen T. Effects of hyaluronic acid (Hyalonect) on callus formation in rabbits. Acta Orthop Traumatol Turc. 2015;49(3):319-25. doi: 10.3944/AOTT.2015.14.0231. PubMed PMID: 26200413.

2. Bahney CS, Jacobs L, Tamai R, Hu D, Luan TF, Wang M, et al. Promoting Endochondral Bone Repair Using Human Osteoarthritic Articular Chondrocytes. Tissue Engineering - Part A 22 (5-6) (pp 427-435), 2016 Date of Publication: 01 Mar 2016. PubMed PMID: 609233860.

3. Bardsley K, Kwarciak A, Freeman C, Brook I, Hatton P, Crawford A. Repair of bone defects in vivo using tissue engineered hypertrophic cartilage grafts produced from nasal chondrocytes. Biomaterials 112:313-323, 2017 Jan. PubMed PMID: 27770634.

4. Cakmak G, Sahin MS, OzdemIr BH, KaradenIz E. Effect of pentoxifylline on healing of segmental bone defects and angiogenesis. Acta orthopaedica et traumatologica turcica 49 (6) (pp 676-682), 2015 Date of Publication: 2015. PubMed PMID: 611671810.

5. Cheung WK, Working DM, Galuppo LD, Leach JK. Osteogenic comparison of expanded and uncultured adipose stromal cells. Cytotherapy. 2010;12(4):554-62. PubMed PMID: 20370353.

6. Fan D, Liu S, Jiang S, Li Z, Mo X, Ruan H, et al. The use of SHP-2 gene transduced bone marrow mesenchymal stem cells to promote osteogenic differentiation and bone defect repair in rat. Journal of Biomedical Materials Research - Part A (no pagination), 2016 Date of Publication: 2016. PubMed PMID: 609701094.

7. Fukui T, Mifune Y, Matsumoto T, Shoji T, Kawakami Y, Kawamoto A, et al. Superior Potential of CD34-Positive Cells Compared to Total Mononuclear Cells for Healing of Nonunion Following Bone Fracture. Cell Transplantation 24(7):1379-93, 2015. PubMed PMID: 24800622.

8. Furuta T, Miyaki S, Ishitobi H, Ogura T, Kato Y, Kamei N, et al. Mesenchymal stem cell-derived exosomes promote fracture healing in a mouse model. Stem Cells Translational Medicine 5 (12) (pp 1620-1630), 2016 Date of Publication: 01 Dec 2016. PubMed PMID: 613345525.

9. Grassmann JP, Schneppendahl J, Sager M, Hakimi AR, Herten M, Loegters TT, et al. The effect of bone marrow concentrate and hyperbaric oxygen therapy on bone repair. Journal of Materials Science-Materials in Medicine. 2015;26(1):5331. PubMed PMID: 25577213.

10. Hao C, Wang Y, Shao L, Liu J, Chen L, Zhao Z. Local Injection of Bone Mesenchymal Stem Cells and Fibrin Glue Promotes the Repair of Bone Atrophic Nonunion In Vivo. Advances in Therapy 33(5):824-33, 2016 May. PubMed PMID: 27098172.

11. He S, Zhao W, Zhang L, Mi L, Du G, Sun C, et al. Low-frequency vibration treatment of bone marrow stromal cells induces bone repair in vivo. Iranian Journal of Basic Medical Sciences 20 (1) (pp 23-28), 2017 Date of Publication: Janaury 2017. PubMed PMID: 613969184.

12. Heo SC, Shin WC, Lee MJ, Kim BR, Jang IH, Choi EJ, et al. Periostin accelerates bone healing mediated by human mesenchymal stem cell-embedded hydroxyapatite/tricalcium phosphate scaffold. PLoS ONE 10 (3) (no pagination), 2015 Article Number: e0116698 Date of Publication: 16 Mar 2015. PubMed PMID: 603024238.

13. Jensen J, Tvedesoe C, Rolfing JH, Foldager CB, Lysdahl H, Kraft DC, et al. Dental pulp-derived stromal cells exhibit a higher osteogenic potency than bone marrow-derived stromal cells in vitro and in a porcine critical-size bone defect model. Sicotj 2:16, 2016 Apr 20. PubMed PMID: 27163105.

14. Kim BS, Lee J. Enhanced bone healing by improved fibrin-clot formation via fibrinogen adsorption on biphasic calcium phosphate granules. Clinical Oral Implants Research 26(10):1203-10, 2015 Oct. PubMed PMID: 24888232.

15. Kim BS, Kim HJ, Choi JG, You HK, Lee J. The effects of fibrinogen concentration on fibrin/atelocollagen composite gel: an in vitro and in vivo study in rabbit calvarial bone defect. Clinical Oral Implants Research 26(11):1302-8, 2015 Nov. PubMed PMID: 25039258.

16. Kim BS, Yang SS, Lee J. Precoating of biphasic calcium phosphate bone substitute with atelocollagen enhances bone regeneration through stimulation of osteoclast activation and angiogenesis. Journal of Biomedical Materials Research Part A 105(5):1446-1456, 2017 May. PubMed PMID: 28177580.

17. Lattanzi W, Parrilla C, Fetoni A, Logroscino G, Straface G, Pecorini G, et al. Ex vivo-transduced autologous skin fibroblasts expressing human Lim mineralization protein-3 efficiently form new bone in animal models. Gene Therapy. 2008;15(19):1330-43. PubMed PMID: 18633445.

18. Levy S, Feduska JM, Sawant A, Gilbert SR, Hensel JA, Ponnazhagan S. Immature myeloid cells are critical for enhancing bone fracture healing through angiogenic cascade. Bone 93 (pp 113-124), 2016 Date of Publication: 01 Dec 2016. PubMed PMID: 612349583.

19. Lin Y, Wang T, Wu L, Jing W, Chen X, Li Z, et al. Ectopic and in situ bone formation of adipose tissue-derived stromal cells in biphasic calcium phosphate nanocomposite. Journal of Biomedical Materials Research. 2007;Part A. 81(4):900-10. PubMed PMID: 17236222.

20. Park SS, Kim SG, Lim SC, Ong JL. Osteogenic activity of the mixture of chitosan and particulate dentin. Journal of Biomedical Materials Research. 2008;Part A. 87(3):618-23. PubMed PMID: 18186071.

21. Peled E, Boss J, Bejar J, Zinman C, Seliktar D. A novel poly(ethylene glycol)-fibrinogen hydrogel for tibial segmental defect repair in a rat model. Journal of Biomedical Materials Research. 2007;Part A. 80(4):874-84. PubMed PMID: 17072852.

22. Santo VE, Ratanavaraporn J, Sato K, Gomes ME, Mano JF, Reis RL, et al. Cell engineering by the internalization of bioinstructive micelles for enhanced bone regeneration. Nanomedicine. 2015;10(11):1707-21.

23. Serrano J, Romo E, Bermudez M, Narayanan AS, Zeichner-David M, Santos L, et al. Bone regeneration in rat cranium critical-size defects induced by Cementum Protein 1 (CEMP1). PLoS ONE [Electronic Resource]. 2013;8(11):e78807. PubMed PMID: 24265720.

24. Song K, Rao NJ, Chen ML, Huang ZJ, Cao YG. Enhanced bone regeneration with sequential delivery of basic fibroblast growth factor and sonic hedgehog. Injury. 2011;42(8):796-802. PubMed PMID: 21367413.

25. Zanchetta P, Lagarde N, Uguen A, Marcorelles P. Mixture of hyaluronic acid, chondroitin 6 sulphate and dermatan sulphate used to completely regenerate bone in rat critical size defect model. Journal of Cranio-Maxillo-Facial Surgery. 2012;40(8):783-7. PubMed PMID: 22464550.

26. Zhang Z, Shively JE. Acceleration of Bone Repair in NOD/SCID Mice by Human Monoosteophils, Novel LL-37-Activated Monocytes. PLoS ONE 8 (7) , 2013 Article Number: e67649 Date of Publication: 03 Jul 2013. 2013. PubMed PMID: 2013422276.

27. Zhang Y, Jing D, Buser D, Sculean A, Chandad F, Miron RJ. Bone grafting material in combination with Osteogain for bone repair: a rat histomorphometric study. Clinical Oral Investigations 20(3):589-95, 2016 Apr. PubMed PMID: 26174082.
