# Supplementary material for: Preclinical therapies to prevent or treat fracture non-union: A systematic review
Source: PLoS One. 2018 Aug 1;13(8):e0201077. doi: 10.1371/journal.pone.0201077 (PMC6070249; doi:10.1371/journal.pone.0201077)
Supplement: S8 Table — (DOCX) [file pone.0201077.s008.docx]

**S8 Table:** Defect repair data for studies evaluating therapies based on vibration or motion (2 therapies, 2 studies)

| **Study** | **Therapy** | **Species** | **Maximum length of survival (days)** | **Outcome** | **Overall effect** |
| --- | --- | --- | --- | --- | --- |
| He 2017[1] | Low frequency vibration + bone marrow stromal cells (BMSCs) | Rabbits | 28 | Low frequency vibrations ideally at 50 Hz promote the differentiation of BMSCs into osteoblasts | ? |
| Puhar 2016[2] | Low magnitude high-frequency vibration | Rabbits | 7 | No significant difference between groups observed | = |

= indicates no difference in bone formation rates between the therapeutic or control groups

? indicates results are unclear, and no effect size could be determined

1. He S, Zhao W, Zhang L, Mi L, Du G, Sun C, et al. Low-frequency vibration treatment of bone marrow stromal cells induces bone repair in vivo. Iranian Journal of Basic Medical Sciences 20 (1) (pp 23-28), 2017 Date of Publication: Janaury 2017. PubMed PMID: 613969184.

2. Puhar I, Ma L, Suleimenova D, Chronopoulos V, Mattheos N. The effect of local application of low-magnitude high-frequency vibration on the bone healing of rabbit calvarial defects-a pilot study. Journal of Orthopaedic Surgery 11(1):159, 2016 Dec 08. PubMed PMID: 27931261.
