# Supplementary material for: Preclinical therapies to prevent or treat fracture non-union: A systematic review
Source: PLoS One. 2018 Aug 1;13(8):e0201077. doi: 10.1371/journal.pone.0201077 (PMC6070249; doi:10.1371/journal.pone.0201077)
Supplement: S9 Table — (DOCX) [file pone.0201077.s009.docx]

**S9 Table:** Defect repair data for studies evaluating therapies based on lights or lasers (3 therapies, 2 studies)

| **Study** | **Therapy** | **Species** | **Maximum length of survival (days)** | **Outcome** | **Overall effect** |
| --- | --- | --- | --- | --- | --- |
| Dereci 2016[1] | Blue LED light | Rats | 21 | Statistically significant increase between control and blue LED light | ↑ |
| Dereci 2016[1] | Low level diode laser light | Rats | 21 | Statistically significant increase between control and low level diode laser light group | ↑ |
| Nascimento 2010[2] | Calcitonin + low level laser therapy | Rats | 21 | Significantly greater bone mineral density compared to the control group and compared to calcitonin alone | ↑ |

↑ indicates statistically significant effect on bone formation in trial therapy compared to control

1. Dereci O, Sindel A, Serap Toru H, Yuce E, Ay S, Tozoglu S. The Comparison of the Efficacy of Blue Light-Emitting Diode Light and 980-nm Low-Level Laser Light on Bone Regeneration. Journal of Craniofacial Surgery 27(8):2185-2189, 2016 Nov. PubMed PMID: 28005786.

2. Nascimento SB, Cardoso CA, Ribeiro TP, Almeida JD, Albertini R, Munin E, et al. Effect of low-level laser therapy and calcitonin on bone repair in castrated rats: a densitometric study. Photomedicine and Laser Surgery. 2010;28(1):45-9. PubMed PMID: 19712023.
