# Supplementary material for: Preclinical therapies to prevent or treat fracture non-union: A systematic review
Source: PLoS One. 2018 Aug 1;13(8):e0201077. doi: 10.1371/journal.pone.0201077 (PMC6070249; doi:10.1371/journal.pone.0201077)
Supplement: S10 Table — (DOCX) [file pone.0201077.s010.docx]

**S10 Table:** Defect repair data for studies evaluating therapies based on gases (3 therapies, 3 studies)

| **Study** | **Therapy** | **Species** | **Maximum length of survival (days)** | **Outcome** | **Overall effect** |
| --- | --- | --- | --- | --- | --- |
| Grassmann 2015[1] | Bone marrow concentrate + hyperbaric oxygen therapy | Rabbits | 42 | Significantly higher bone defect consolidation in hyperbaric oxygen + bone marrow concentrate group compared to bone marrow concentrate group alone | ↑ |
| Koga 2014[2] | Cutaneous CO2 application | Rats | 28 | No significant difference between control and therapeutic groups at 28 days | = |
| Rocha 2015[3] | Hyperbaric oxygen | Rats | 7 | No significant difference in percentage of bone neoformation between control and therapeutic groups at day 7 | = |

↑ indicates statistically significant effect on bone formation in trial therapy compared to control

= indicates no difference in bone formation rates between the therapeutic or control groups

1. Grassmann JP, Schneppendahl J, Sager M, Hakimi AR, Herten M, Loegters TT, et al. The effect of bone marrow concentrate and hyperbaric oxygen therapy on bone repair. Journal of Materials Science-Materials in Medicine. 2015;26(1):5331. PubMed PMID: 25577213.

2. Koga T, Niikura T, Lee SY, Okumachi E, Ueha T, Iwakura T, et al. Topical cutaneous CO2 application by means of a novel hydrogel accelerates fracture repair in rats. Journal of Bone & Joint Surgery - American Volume. 2014;96(24):2077-84. PubMed PMID: 25520342.

3. Rocha FS, Gomes Moura CC, Rocha Rodrigues DB, Zanetta-Barbosa D, Nakamura Hiraki KR, Dechichi P. Influence of hyperbaric oxygen on the initial stages of bone healing. Oral surgery, oral medicine, oral pathology and oral radiology 120 (5) (pp 581-587), 2015 Date of Publication: 01 Nov 2015. PubMed PMID: 612427975.
