# Supplementary material for: Preclinical therapies to prevent or treat fracture non-union: A systematic review
Source: PLoS One. 2018 Aug 1;13(8):e0201077. doi: 10.1371/journal.pone.0201077 (PMC6070249; doi:10.1371/journal.pone.0201077)
Supplement: S11 Table — (DOCX) [file pone.0201077.s011.docx]

**S11 Table:** Defect repair data for studies evaluating therapies based on human proteins or hormones (59 therapies, 42 studies)

| **Study** | **Therapy** | **Species** | **Maximum length of survival (days)** | **Outcome** | **Overall effect** |
| --- | --- | --- | --- | --- | --- |
| Ackerson 2014[1] | Liver X receptor agonist | Mice | 35 | No qualitative differences found between control and therapeutic groups | = |
| Canter 2010[2] | Chitosan + TGF-beta-2 | Rats | 98 | Less effect on defect repair when compared to autograft alone | ↓ |
| Canter 2010[2] | Chitosan + BMP-2 | Rats | 98 | No difference between autograft and chitosan/BMP group | = |
| Canter 2010[2] | Chitosan + TGF-beta-2 + BMP-2 | Rats | 98 | No difference between autograft and TGF/BMP group | = |
| Chen 2017[3] | Calcitonin gene related peptide (1μM) | Rabbits | 168 | Compared to sham surgery group and gelatin alone group all CGRP groups had a significantly higher bone volume density: the bone volume density of the group implanted with 1μM or 10nM was significantly higher than the group implanted with 0.1nM | ↑ |
| Chen 2017[3] | Calcitonin gene related peptide (10nM) | Rabbits | 168 | Compared to sham surgery group and gelatin alone group all CGRP groups had a significantly higher bone volume density | ↑ |
| Chen 2017[3] | Calcitonin gene related peptide (0.1nM) | Rabbits | 168 | Compared to sham surgery group and gelatin alone group all CGRP groups had a significantly higher bone volume density | ↑ |
| Chen 2017[3] | Substance P (10nM) | Rabbits | 168 | Compared to the sham surgery group and the gelatin alone group, the ratio of bone volume / total volume in the groups implanted with 1 μM or 10 nM of substance P was superior to that of the group implanted with 0.1 nM substance P | → |
| Chen 2017[3] | Substance P (0.1nM) | Rabbits | 168 | Compared to the sham surgery group and the gelatin alone group, the ratio of bone volume / total volume in the groups implanted with 1 μM or 10 nM of substance P was superior to that of the group implanted with 0.1 nM substance P | ? |
| Chen 2017[3] | Substance P (1μM) | Rabbits | 168 | Bone defect partially healed after 6 months | ? |
| Choi 2015[4] | Cartilage oligomeric matrix protein-angiopoietin 1 (COMP-Ang1) | Mice | 21 | COMP-Ang1 significantly induced bone formation | ↑ |
| Choi 2015[4] | Cartilage oligomeric matrix protein-angiopoietin 1 (COMP-Ang1) + BMP2 | Mice | 21 | Combined delivery of COMP-Ang1 and BMP2 produced greater bone by approximately 1.5 times | → |
| Dosier 2015[5] | Alginate hydrogel to deliver BMPs | Rats | 84 | Significantly more bone observed in BMP2 positive hydrogels than in control group | ↑ |
| Fan 2017[6] | Phenamil + BMP | Rats | 86 | Addition of BMP to phenamil synergistically augmented bone healing, resulting in almost complete bone healing | → |
| Fukui 2015[7] | Human granulocyte colony stimulating factor mobilised CD34 positive cells | Rats | 84 | Frequency of bone union was significantly greater in the CD34 positive group than the control at week 12 | ↑ |
| Fukui 2015[7] | Human granulocyte colony stimulating factor mobilised mononuclear cells | Rats | 84 | Non-union healing was observed in 30% of rats at week 12 in the MNC group, compared to no bridging callus formation during 12 weeks of observation in the control group | → |
| Glass 2011[8] | TNF-alpha | Mice | 28 | Significantly greater callus mineralisation in therapeutic group compared to control group | = |
| Gordjestani 2006[9] | Osteopontin | Rabbits | 210 | No statistical difference in bone formation between therapeutic and control groups | = |
| Gordjestani 2007[10] | Osteopontin | Rabbits | 210 | Significantly higher bone formation in therapeutic group than in control group | ↑ |
| Heo 2015[11] | Periostin | Mice | 56 | No significant reduction in gap of defect compared to control | = |
| Heo 2015[11] | Periostin + human adipose stromal cells | Mice | 56 | Significant reduction in bone gap compared to control | ↑ |
| Hu 2013[12] | Glycoprotein non-metastatic melanoma protein B | Rats | 28 | Therapeutic intervention had a pro-bone regeneration activity | → |
| Jia 2014[13] | Casein kinase 2 interacting protein 1 | Rats | 90 | Increase in bone formation in therapeutic group compared to control | → |
| Kamolratanakul 2011[14] | BMP with EP4A receptor agonist | Mice | 28 | Greater bone formation in therapeutic group compared to control group | → |
| Kramer 2008[15] | Tissue inhibitor of metalloproteinases (TIMP) 1μg | Rats | 56 | Tissue density was higher in therapeutic group than in the control group | ↑ |
| Kramer 2008[15] | Tissue inhibitor of metalloproteinases (TIMP) 10μg | Rats | 56 | Tissue density was higher than control, but lower than groups treated with BMP-2 | → |
| Lau 2013[16] | COX-2 | Mice | 21 | Improved bone healing in therapeutic group compared to control group | → |
| Lee 2015[17] | Heparan mediated fibroblast growth factor | Mice | 60 | No evidence of bone formation in either group | = |
| Li 2011[18] | Nell-1 0.6mg/ml | Rats | 84 | Significantly greater defect mineralisation compared to control group | ↑ |
| Li 2011[18] | Nell-1 1.5mg/ml | Rats | 84 | Significantly greater defect mineralisation compared to control group, and compared to lower dose treatment group | ↑ |
| Li 2016[19] | Erythropoietin on deproteinised bovine bone (DBB) scaffold | Goats | 84 | Significantly higher grey values in therapeutic group compared to DBB scaffold alone group at 12 weeks, but no difference between therapeutic group and autogenous cancellous bone graft | ↑ |
| Lipinsky 2015[20] | Prostaglandin | Rabbits | 30 | Significant difference in bone callus thickening in experimental group compared to control | ↑ |
| Liu 2007[21] | FGF-2 and TNF-r1 | Rats | 14 | New bone formation significantly higher in treatment group compared to control group | ↑ |
| Liu 2015[22] | Hepatocyte growth factor | Rabbits | 56 | Bone tissue repair in the experimental side was better than the control side | → |
| Montoya 2014[23] | Cementum attachment protein | Rats | 56 | Significantly greater new bone formation in therapeutic group compared to control group | ↑ |
| Morishita 2010[24] | 5μg BMP + 1000μg BBP | Rats | 56 | Significantly greater bone volume in therapeutic group compared to control group | ↑ |
| Morishita 2010[24] | 2μg BMP + 1000μg BBP | Rats | 56 | No significant increase in bone volume in therapeutic group compared to control group | = |
| Morishita 2010[24] | 1000μg BBP | Rats | 56 | Significantly greater bone volume in therapeutic group compared to control group | ↑ |
| Nascimento 2010[25] | Calcitonin | Rats | 21 | Significantly greater bone mineral density in therapeutic group compared to the control group | ↑ |
| Nascimento 2010[25] | Calcitonin + low level laser therapy | Rats | 21 | Significantly greater bone mineral density compared to the control group and compared to calcitonin alone | ↑ |
| Ogilvie 2012[26] | Vascular endothelial growth factor | Mice | 27 | No significant difference in amount of new bone formation between therapeutic and control groups | = |
| Omlor 2016[27] | Erythropoietin (local) | Rabbits | 84 | Increased bone formation compared to control | → |
| Omlor 2016[27] | Erythropoietin (systemic) | Rabbits | 84 | Increased bone formation compared to control | → |
| Park 2013[28] | Insulin | Rats | 28 | No statistical difference seen in histological scoring | = |
| Patel 2008[29] | Vascular endothelial growth factor | Rats | 84 | No statistical difference seen between groups | = |
| Patel 2008[29] | Vascular endothelial growth factor + BMP2 | Rats | 84 | Significantly greater rates of union in therapeutic group compared to control group | ↑ |
| Rozen 2007[30] | Interleukin + parathyroid hormone | Rats | 42 | Full radiological healing in therapeutic group; no mention of radiological healing in control group. Increased mechanical resistance in therapeutic group compared to control group. | ? |
| Servin-Trujillo 2011[31] | Transforming growth factor-beta1 | Dogs | 35 | No differences found in bone callus formation between therapeutic and control groups | = |
| Sinha 2009[32] | Arginine and lysine amino acids | Rabbits | 140 | Improved histological bone healing in therapeutic group compared to control group | → |
| Song 2011[33] | Co-transduction of bone marrow-derived mesenchymal stem cells by basic fibroblast growth factor | Rats | 28 | Significantly higher bone area density in genetically modified therapeutic group compared to control | ↑ |
| Tang 2017[34] | Prostaglandin | Rats | 28 | No significant difference between groups | = |
| Trejo 2010[35] | Parathyroid hormone related protein (osteostatin) | Rabbits | 56 | Greater bone growth in therapeutic group compared to control group | → |
| Ugras 2013[36] | Glucosamine sulphate | Rats | 28 | No significant difference in new bone between therapeutic and control groups | = |
| Wan 2014[37] | Erythropoietin | Mice | 28 | Enhanced bone consolidation in therapeutic group compared to control group | → |
| Wixted 2009[38] | Leukotriene antagonist | Mice | 28 | Net increase in callus size relative to control | → |
| Woodruff 2007[39] | Heparan sulphate doped fibrin glue | Rats | 90 | Significantly higher mineralised bone present in therapeutic group when compared to control group | ↑ |
| Xu 2011[40] | Vascular endothelial growth factor | Rats | 56 | Significantly greater bone mineral density in therapeutic group when compared to control group | ↑ |
| Young 2009[41] | Vascular endothelial growth factor + BMP2 | Rats | 84 | No increase in bone formation in therapeutic group compared to BMP alone | = |
| Zhao 2007[42] | Vascular endothelial growth factor plasmid | Rabbits | 84 | No significant difference between therapeutic and control groups | = |

↑ indicates statistically significant effect on bone formation in trial therapy compared to control

→ indicates greater bone formation in trial therapy compared to control, but the effect did not reach statistical significance

= indicates no difference in bone formation rates between the therapeutic or control groups

↓ indicates less effect on bone formation in trial therapy compared to control

? indicates results are unclear, and no effect size could be determined

1. Ackerson RM, Shum LC, Berry AR, Bucknell AL, King KB. In vivo model to measure bone repair efficacy of nanoparticle-based drug delivery. Orthopedics. 2014;37(8):e707-11. PubMed PMID: 25102506.

2. Canter HI, Vargel I, Korkusuz P, Oner F, Gungorduk DB, Cil B, et al. Effect of use of slow release of bone morphogenetic protein-2 and transforming growth factor-Beta-2 in a chitosan gel matrix on cranial bone graft survival in experimental cranial critical size defect model. Annals of Plastic Surgery. 2010;64(3):342-50. PubMed PMID: 20179488.

3. Chen J, Liu W, Zhao J, Sun C, Chen J, Hu K, et al. Gelatin microspheres containing calcitonin gene-related peptide or substance P repair bone defects in osteoporotic rabbits. Biotechnology Letters 39(3):465-472, 2017 Mar. PubMed PMID: 27909823.

4. Choi H, Jeong BC, Hur SW, Kim JW, Lee KB, Koh JT. The angiopoietin-1 variant COMP-Ang1 enhances BMP2-induced bone regeneration with recruiting pericytes in critical sized calvarial defects. PLoS ONE 10 (10) (no pagination), 2015 Article Number: e0140502 Date of Publication: 14 Oct 2015. PubMed PMID: 607111800.

5. Dosier CR, Uhrig BA, Willett NJ, Krishnan L, Li MT, Stevens HY, et al. Effect of cell origin and timing of delivery for stem cell-based bone tissue engineering using biologically functionalized hydrogels. Tissue engineering. 2015;Part A.. 21(1-2):156-65. PubMed PMID: 25010532.

6. Fan J, Guo M, Im CS, Pi-Anfruns J, Cui ZK, Kim S, et al. Enhanced Mandibular Bone Repair by Combined Treatment of Bone Morphogenetic Protein 2 and Small-Molecule Phenamil. Tissue Engineering - Part A 23 (5-6) (pp 195-207), 2017 Date of Publication: March 2017. PubMed PMID: 614720791.

7. Fukui T, Mifune Y, Matsumoto T, Shoji T, Kawakami Y, Kawamoto A, et al. Superior Potential of CD34-Positive Cells Compared to Total Mononuclear Cells for Healing of Nonunion Following Bone Fracture. Cell Transplantation 24(7):1379-93, 2015. PubMed PMID: 24800622.

8. Glass GE, Chan JK, Freidin A, Feldmann M, Horwood NJ, Nanchahal J. TNF-alpha promotes fracture repair by augmenting the recruitment and differentiation of muscle-derived stromal cells. Proceedings of the National Academy of Sciences of the United States of America. 2011;108(4):1585-90. PubMed PMID: 21209334.

9. Gordjestani M, Dermaut L, De Ridder L, De Waele P, De Leersnijder W, Bosman F. Osteopontin and bone metabolism in healing cranial defects in rabbits. International Journal of Oral and Maxillofacial Surgery 35 (12) (pp 1127-1132), 2006 Date of Publication: December 2006. 2006. PubMed PMID: 2006551786.

10. Gordjestani M, Dermaut L, De Ridder L, De Waele P. Osteopontin and bone repair in rabbit tibial defect. European Journal of Orthopaedic Surgery and Traumatology 17 (2) (pp 139-145), 2007 Date of Publication: March 2007. 2007. PubMed PMID: 2007117353.

11. Heo SC, Shin WC, Lee MJ, Kim BR, Jang IH, Choi EJ, et al. Periostin accelerates bone healing mediated by human mesenchymal stem cell-embedded hydroxyapatite/tricalcium phosphate scaffold. PLoS ONE 10 (3) (no pagination), 2015 Article Number: e0116698 Date of Publication: 16 Mar 2015. PubMed PMID: 603024238.

12. Hu X, Zhang P, Xu Z, Chen H, Xie X. GPNMB enhances bone regeneration by promoting angiogenesis and osteogenesis: potential role for tissue engineering bone. Journal of Cellular Biochemistry. 2013;114(12):2729-37. PubMed PMID: 23794283.

13. Jia S, Yang X, Song W, Wang L, Fang K, Hu Z, et al. Incorporation of osteogenic and angiogenic small interfering RNAs into chitosan sponge for bone tissue engineering. International Journal of Nanomedicine. 2014;9:5307-16. PubMed PMID: 25429217.

14. Kamolratanakul P, Hayata T, Ezura Y, Kawamata A, Hayashi C, Yamamoto Y, et al. Nanogel-based scaffold delivery of prostaglandin E(2) receptor-specific agonist in combination with a low dose of growth factor heals critical-size bone defects in mice. Arthritis & Rheumatism. 2011;63(4):1021-33. PubMed PMID: 21190246.

15. Kramer FJ, Meyer M, Morgan D, Forssmann WG, Standker L, Schliephake H, et al. Tissue inhibitor of metalloproteinases II (TIMP-2) is an osteoanabolic factor in vitro and in vivo. European Journal of Medical Research 13 (6) (pp 292-298), 2008 Date of Publication: 24 Jun 2008. 2008. PubMed PMID: 2008340676.

16. Lau KH, Kothari V, Das A, Zhang XB, Baylink DJ. Cellular and molecular mechanisms of accelerated fracture healing by COX2 gene therapy: studies in a mouse model of multiple fractures. Bone. 2013;53(2):369-81. PubMed PMID: 23314071.

17. Lee JH, Lee YJ, Cho HJ, Kim DW, Shin H. The incorporation of bFGF mediated by heparin into PCL/gelatin composite fiber meshes for guided bone regeneration. Drug Delivery & Translational Research. 2015;5(2):146-59. PubMed PMID: 25787740.

18. Li W, Zara JN, Siu RK, Lee M, Aghaloo T, Zhang X, et al. Nell-1 enhances bone regeneration in a rat critical-sized femoral segmental defect model. Plastic & Reconstructive Surgery. 2011;127(2):580-7. PubMed PMID: 21285762.

19. Li D, Deng L, Xie X, Yang Z, Kang P. Evaluation of the osteogenesis and angiogenesis effects of erythropoietin and the efficacy of deproteinized bovine bone/recombinant human erythropoietin scaffold on bone defect repair. Journal of Materials Science-Materials in Medicine 27(6):101, 2016 Jun. PubMed PMID: 27091043.

20. Lipinsky PV, Sirotin IV, Skoroglyadov AV, Ivkov AV, Oettinger AP, Krynetskiy EE, et al. Effects of prostaglandin E1 on callus formation in rabbits. BMC Musculoskeletal Disorders 16:247, 2015 Sep 10. PubMed PMID: 26359236.

21. Liu ZD, Zhong JL, Xu Y, Miao J. Recombinant human fibroblastic growth factor-2 with soluble tumor necrosis factor receptor-1 facilitates fracture repair in rats with type 2 diabetes mellitus. Journal of Clinical Rehabilitative Tissue Engineering Research 11 (32) (pp 6505-6508), 2007 Date of Publication: 12 Aug 2007. 2007. PubMed PMID: 2007458058.

22. Liu P, Guo L, Huang L, Zhao D, Zhen R, Hu X, et al. Effect of semisynthetic extracellular matrix-like hydrogel containing hepatocyte growth factor on repair of femoral neck defect in rabbits. International journal of clinical and experimental medicine 8(5):7374-80, 2015. PubMed PMID: 26221278.

23. Montoya G, Arenas J, Romo E, Zeichner-David M, Alvarez M, Narayanan AS, et al. Human recombinant cementum attachment protein (hrPTPLa/CAP) promotes hydroxyapatite crystal formation in vitro and bone healing in vivo. Bone. 2014;69 (pp 154-164), 2014. Date of Publication:December 01. PubMed PMID: 2014850110.

24. Morishita Y, Naito M, Miyazaki M, He W, Wu G, Wei F, et al. Enhanced effects of BMP-binding peptide combined with recombinant human BMP-2 on the healing of a rodent segmental femoral defect. Journal of Orthopaedic Research. 2010;28(2):258-64. PubMed PMID: 19639633.

25. Nascimento SB, Cardoso CA, Ribeiro TP, Almeida JD, Albertini R, Munin E, et al. Effect of low-level laser therapy and calcitonin on bone repair in castrated rats: a densitometric study. Photomedicine and Laser Surgery. 2010;28(1):45-9. PubMed PMID: 19712023.

26. Ogilvie CM, Lu C, Marcucio R, Lee M, Thompson Z, Hu D, et al. Vascular endothelial growth factor improves bone repair in a murine nonunion model. Iowa Orthopaedic Journal. 2012;32:90-4. PubMed PMID: 23576927.

27. Omlor GW, Kleinschmidt K, Gantz S, Speicher A, Guehring T, Richter W. Increased bone formation in a rabbit long-bone defect model after single local and single systemic application of erythropoietin. Acta Orthopaedica 87 (4) (pp 425-431), 2016 Date of Publication: 03 Jul 2016. PubMed PMID: 610983853.

28. Park AG, Paglia DN, Al-Zube L, Hreha J, Vaidya S, Breitbart E, et al. Local insulin therapy affects fracture healing in a rat model. Journal of Orthopaedic Research. 2013;31(5):776-82. PubMed PMID: 23238765.

29. Patel ZS, Young S, Tabata Y, Jansen JA, Wong ME, Mikos AG. Dual delivery of an angiogenic and an osteogenic growth factor for bone regeneration in a critical size defect model. Bone. 2008;43(5):931-40. PubMed PMID: 18675385.

30. Rozen N, Lewinson D, Bick T, Jacob ZC, Stein H, Soudry M. Fracture repair: modulation of fracture-callus and mechanical properties by sequential application of IL-6 following PTH 1-34 or PTH 28-48. Bone. 2007;41(3):437-45. PubMed PMID: 17599848.

31. Servin-Trujillo MA, Reyes-Esparza JA, Garrido-Farina G, Flores-Gazca E, Osuna-Martinez U, Rodriguez-Fragoso L. Use of a graft of demineralized bone matrix along with TGF-beta1 leads to an early bone repair in dogs. Journal of Veterinary Medical Science. 2011;73(9):1151-61. PubMed PMID: 21566397.

32. Sinha S, Goel SC. Effect of amino acids lysine and arginine on fracture healing in rabbits: A radiological and histomorphological analysis. Indian Journal of Orthopaedics. 2009;43(4):328-34. PubMed PMID: 19838381.

33. Song K, Rao NJ, Chen ML, Huang ZJ, Cao YG. Enhanced bone regeneration with sequential delivery of basic fibroblast growth factor and sonic hedgehog. Injury. 2011;42(8):796-802. PubMed PMID: 21367413.

34. Tang Q, Chen LL, Wei F, Sun WL, Lei LH, Ding PH, et al. Effect of 15-Deoxy-delta-prostaglandin J2 Nanocapsules on Inflammation and Bone Regeneration in a Rat Bone Defect Model. Chinese Medical Journal 130(3):347-356, 2017 5th Feb. PubMed PMID: 28139520.

35. Trejo CG, Lozano D, Manzano M, Doadrio JC, Salinas AJ, Dapia S, et al. The osteoinductive properties of mesoporous silicate coated with osteostatin in a rabbit femur cavity defect model. Biomaterials. 2010;31(33):8564-73. PubMed PMID: 20727584.

36. Ugras A, Guzel E, Korkusuz P, Kaya I, Dikici F, Demirbas E, et al. Glucosamine-sulfate on fracture healing. Ulusal Travma ve Acil Cerrahi Dergisi 19 (1) (pp 8-12), 2013 Date of Publication: 2013. 2013. PubMed PMID: 2013171007.

37. Wan L, Zhang F, He Q, Tsang WP, Lu L, Li Q, et al. EPO promotes bone repair through enhanced cartilaginous callus formation and angiogenesis.[Erratum appears in PLoS One. 2014;9(10):e111830]. PLoS ONE [Electronic Resource]. 2014;9(7):e102010. PubMed PMID: 25003898.

38. Wixted JJ, Fanning PJ, Gaur T, O'Connell SL, Silva J, Mason-Savas A, et al. Enhanced fracture repair by leukotriene antagonism is characterized by increased chondrocyte proliferation and early bone formation: a novel role of the cysteinyl LT-1 receptor. Journal of Cellular Physiology. 2009;221(1):31-9. PubMed PMID: 19544365.

39. Woodruff MA, Rath SN, Susanto E, Haupt LM, Hutmacher DW, Nurcombe V, et al. Sustained release and osteogenic potential of heparan sulfate-doped fibrin glue scaffolds within a rat cranial model. Journal of Molecular Histology. 2007;38(5):425-33. PubMed PMID: 17849224.

40. Xu Cz, Yang Wg, He Xf, Zhou Lt, Han Xk, Xu Xf. Vascular endothelial growth factor and nano-hydroxyapatite/collagen composite in the repair of femoral defect in rats. Journal of Clinical Rehabilitative Tissue Engineering Research 15 (38) (pp 7118-7122), 2011 Date of Publication: 2011. 2011. PubMed PMID: 2012467917.

41. Young S, Patel ZS, Kretlow JD, Murphy MB, Mountziaris PM, Baggett LS, et al. Dose effect of dual delivery of vascular endothelial growth factor and bone morphogenetic protein-2 on bone regeneration in a rat critical-size defect model. Tissue engineering. 2009;Part A.. 15(9):2347-62. PubMed PMID: 19249918.

42. Zhao DM, Yang JF, Wu SQ, Qiu LP, Liu JL, Wang HB, et al. [Effect of vascular endothelial growth factor 165 gene transfection on repair of bone defect: experiment with rabbits]. Chung-Hua i Hsueh Tsa Chih [Chinese Medical Journal]. 2007;87(25):1778-82. PubMed PMID: 17919388.
